# Isolation, Characterization and Antiproliferative Activity of New Metabolites from the South African Endemic Red Algal Species *Laurencia alfredensis*

**DOI:** 10.3390/molecules22040513

**Published:** 2017-03-23

**Authors:** Godwin A. Dziwornu, Mino R. Caira, Jo-Anne de la Mare, Adrienne L. Edkins, John J. Bolton, Denzil R. Beukes, Suthananda N. Sunassee

**Affiliations:** 1 Department of Chemistry, University of Cape Town, Rondebosch 7701, South Africa; dzwgod001@myuct.ac.za (G.A.D.); mino.caira@uct.ac.za (M.R.C.); 2 Biomedical Biotechnology Research Unit, Department of Biochemistry and Microbiology, Rhodes University, Grahamstown 6140, South Africa; j.delamare@ru.ac.za (J.-A.d.l.M.); a.edkins@ru.ac.za (A.L.E.); 3 Department of Biological Sciences, University of Cape Town, Rondebosch 7701, South Africa; john.bolton@uct.ac.za; 4 Marine Research (Ma-Re) Institute, University of Cape Town, Rondebosch 7701, South Africa; 5 School of Pharmacy Department of Pharmaceutical Chemistry, University of the Western Cape, Bellville 7535, South Africa; dbeukes@uwc.ac.za; 6 South African Medical Research Council Drug Discovery and Development Research Unit, University of Cape Town, Rondebosch 7701, South Africa

**Keywords:** labdane-type diterpenes, polyether triterpenes, cholestane-type ecdysteroids, glycolipid, antiproliferative activity, Rhodomelaceae

## Abstract

The marine red algae of the genus *Laurencia* have been widely studied for their structurally diverse and biologically active secondary metabolites. We report here the natural product investigation of the organic extract of a newly identified South African endemic species, *Laurencia alfredensis*. A sequence of column chromatography, preparative TLC and normal phase HPLC resulted in the isolation of eleven compounds comprising three labdane-type diterpenes (**1**–**3**), four polyether triterpenes (**4**–**7**), three cholestane-type ecdysteroids (**8**–**10**) and a glycolipid (**11**). Compounds **1**–**3**, **5**–**8** and **10** have not previously been reported, while compound **9** is reported here for the first time from a natural source and the known compound **11** isolated for the first time from the genus *Laurencia*. The structural elucidation and the relative configuration assignments of the compounds were accomplished by extensive use of 1D- and 2D-NMR, HR-ESI-MS, UV and IR spectroscopic techniques, while the absolute configuration of compound **1** was determined by single-crystal X-ray diffraction analysis. All compounds were evaluated against the MDA-MB-231 breast and HeLa cervical cancer cell lines. Compound **2** exhibited low micromolar antiproliferative activity (IC_50_ = 9.3 µM) against the triple negative breast carcinoma and compound **7** was similarly active (IC_50_ = 8.8 µM) against the cervical cancer cell line.

## 1. Introduction

The family of red algae (Rhodomelaceae) comprises over 1000 recognized species globally. The genus *Laurencia* (Order Ceramiales) in this family has about 144 identified species [1]. It is an abundant source of structurally diverse halogenated and non-halogenated secondary metabolites and thus the most investigated of the Rhodomelaceae [2,3,4]. Halogenated compounds are involved in chemical defense from grazers and, in the genus *Laurencia*, they are contained in special cell vacuoles known as corps-en-cerise (“cherry bodies”, in French), which can be seen using a compound microscope in surface cells in live material [5]. These secondary metabolites include terpenoids, non-terpenoid C_15_ acetogenins and indoles. The terpenoids comprise about nine different major carbon backbones of sesquiterpenes, six of diterpenes and, to date, three of polyether triterpenes [3]. Isolated C_15_ acetogenins, which arise from fatty acid metabolism [6], are grouped into 12 different classes as either linear molecules or cyclic ethers with varying size of the oxygenated rings present in the latter [3]. Several brominated indoles have been reported from the *L. brongniartii* [7,8,9,10,11], *L. decumbens* [12] and *L. similis* [13,14,15] species only.

**Figure 1:**
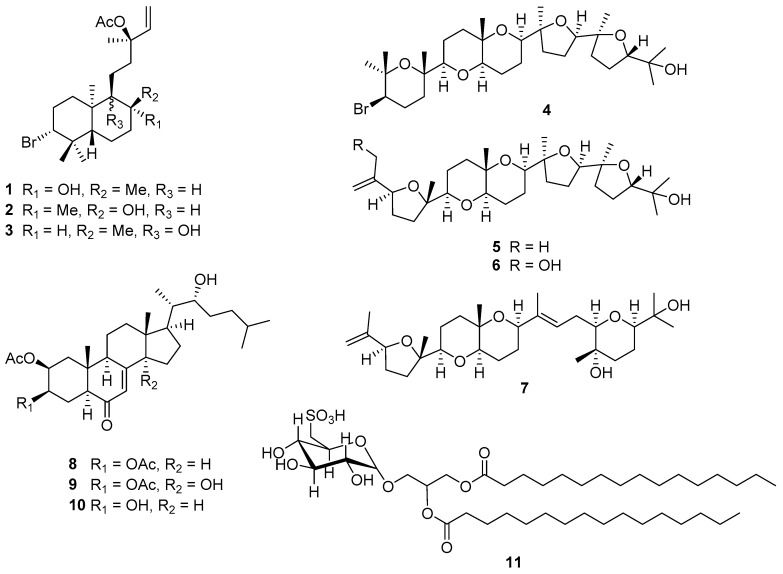
The secondary metabolites (**1**–**11**) isolated from *Laurencia alfredensis*.

*Laurencia alfredensis* is a newly described species of the genus and is endemic to South Africa. The material investigated here was collected on a rocky intertidal seashore at Three Sisters, north of Port Alfred in the Eastern Cape Province of the Republic of South Africa in 2011 [16]. Based on the interesting chemical diversity of secondary metabolites reported from the genus, we undertook a chemical investigation of the constituents of this new species and evaluated their antiproliferative effects against the MDA-MB-231 and HeLa cancer cell lines. Our study has led to the isolation and structural elucidation of 11 compounds, comprising eight newly reported compounds along with the known polyether triterpene saiyacenol B (**4**) [17], the synthetic compound **9** [18], and the glycolipid, 1,2-di-*O*-palmitoyl-3-*O*-(6-sulfo-α-d-quinovopyranosyl)-glycerol (**11**) [19] (Figure 1). Except for compound **4**, none of the compounds isolated have previously been reported from the genus *Laurencia*.

## 2. Results and Discussion

### 2.1. Structural Elucidation of Labdane-Type Diterpenes *(**1**–**3**)*

Compound **1** (Figure 1) was obtained as a white crystalline solid after recrystallization from methanol. HR-ESI-MS (*m*/*z* 451.1824, 453.1792 [M + Na]^+^, calcd. 451.1824 for C_22_H_37_^79^BrO_3_Na) data established a molecular formula of C_22_H_37_BrO_3_, signifying the presence of four degrees of unsaturation. The ^1^H-NMR spectrum exhibited signals of six sharp methyl groups (δ_H_ 0.95, s; 1.06, s; 1.20, s; 1.26, s; 1.52, s) including a characteristic acetate methyl singlet at δ_H_ 2.00 (H_3_-22), several overlapping multiplets at δ_H_ 1.00–2.35 characteristic of a terpenoid backbone, three methine signals with a coupling pattern indicative of the presence of a vinyl group (δ_H_ 5.13, dd, *J* = 0.9, 17.5 Hz, H-15a; 5.15, dd, *J* = 0.9, 11.0 Hz, H-15b; 5.94, dd, *J* = 11.0, 17.5 Hz, H-14) and a methine proton (δ_H_ 3.92, dd, *J* = 4.1, 12.8 Hz, H-3) (Table 1).

The ^13^C-NMR spectrum for compound **1** indicated the presence of 22 non-equivalent carbons including characteristic signals which supported the presence of an ester group (δ_C_ 169.8) and two olefinic carbons (δ_C_ 113.4 and 141.6). The coupling constants recorded for the vicinal coupling between H-3 (δ_H_ 3.92, dd, *J* = 4.1, 12.8 Hz) and H_2_-2, and H-5 (δ_H_ 1.08, dd, *J* = 2.5, 10.9 Hz) and H_2_-6 were consistent with the ^3^*J* diaxial coupling (*J* = 9–12 Hz) and ^3^*J* axial-equatorial coupling (*J* = 2–4 Hz) observed in substituted cyclohexane rings. The four degrees of unsaturation were therefore accounted for by one carbonyl group, one olefinic double bond and two substituted cyclohexane rings. Compound **1** was therefore deduced to be a bicyclic diterpenoid molecule.

**Figure 2:**
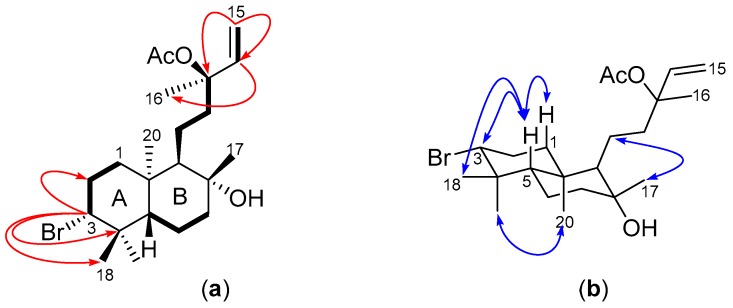
(**a**) COSY (**bold bonds**) and key HMBC (red arrows) of **1**; and (**b**) key ROESY (blue arrows) for compound **1**.

The HSQC-DEPT spectrum showed cross-peaks attributed to six methyl, seven diastereotopic methylene, four methine and five quaternary carbons. Four ^1^H-^1^H COSY spin systems (Figure 2a) were observed, comprising three similar methine proton→methylene protons→methylene protons coupling patterns and the ABX coupling pattern of the vinyl protons. Key HMBC correlations included the long range coupling of H-3 to C-4 (δ_C_ 39.5), C-18 (δ_C_ 17.7) and C-19 (δ_C_ 30.6), and H-14 to C-13 (δ_C_ 82.9) and C-16 (δ_C_ 23.5) (Figure 2a). The chemical shifts of the methine carbon signals at δ_C_ 69.7 and δ_C_ 82.9 suggested the presence of the bromine atom and the acetate group at C-3 and C-13, respectively. Hence, the backbone structure of compound **1** was found to be consistent with that of a brominated labdane-type diterpene, similar to the known compounds Isoconncindiol and Pinnatol A that have both been previously isolated from the *Laurencia* genus [20,21,22].

The presence of ROE enhancements between H_2_-1b, H-3 and H_3_-18 signified they occupied the same face of the cyclohexane ring, as did H_3_-19 and H_3_-20 (Figure 2b). Moreover, a ROESY correlation present between H_3_-20 and H-9 but not H-5 and H_3_-17 was indicative of the *syn*-*ent* configuration about the cyclohexane ring junction and the equatorial orientation of H_3_-17 in ring B. The structure of **1** was successfully assigned as the 13-acetyl derivative of the known C-3 brominated labdane diterpene Pinnatol A [22].

**Figure 3:**
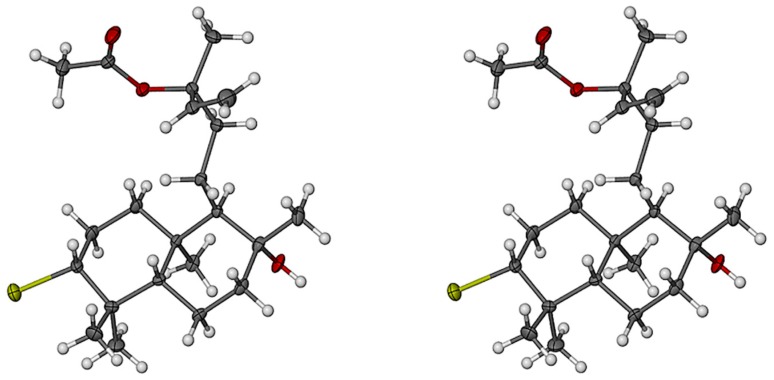
Stereoscopic view of **1** (absolute configuration). Non-H atoms are drawn as thermal ellipsoids at the 40% probability level and H atoms as spheres of arbitrary size. (Color code: C gray, H white, O red, Br yellow.)

X-ray diffraction analysis of **1** confirmed the relative stereochemistry predicted from the ROESY data. Furthermore, the absolute configuration of **1** (Figure 3) was unequivocally determined via the anomalous X-ray scattering of the bromine atom. Both cyclohexane rings assume the more stable chair conformation with a *syn*-*ent* configuration at the ring junction. Protons H-1b, H-3, H-5 and H_3_-18 occupy the same face of ring A whilst H_3_-19 and H_3_-20 occupy the opposite face. The equatorial orientation of H_3_-17 compared to H_3_-20 supports the lack of ROESY enhancement between the two methyl groups.

**Figure 4:**
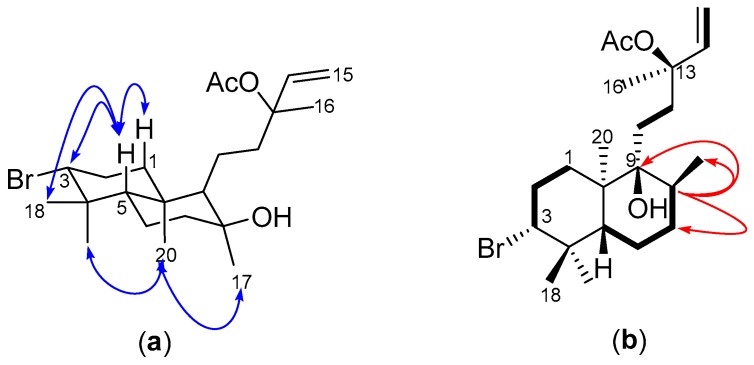
(**a**) Key ROESY (blue arrows) of compound **2**; and (**b**) COSY (bold bonds) and key HMBC (red arrows) of compound **3**.

Compounds **2** and **3** gave similar HR-ESI-MS ion peaks (*m*/*z* 451.1821, 453.1809 [M + Na]^+^, calcd. 451.1824 for C_22_H_37_^79^BrO_3_Na) to **1**, suggesting they were isomeric and this was indeed supported by the high degree of similarity in their NMR data. In fact, both 1D- and 2D-NMR data acquired for compound **2**, except for the ROESY data, were congruent with the NMR spectra for **1**. However, subtle differences in the proton chemical shifts of H-9 (δ_H_ 0.95, m), H-20 (δ_H_ 1.09, s), H-17 (δ_H_ 1.43, s) and H_2_-11 (δ_H_ 1.28, m; 1.71, m) (Table 1) were observed in the ^1^H-NMR spectrum of **2** compared to **1**. The presence of ROESY cross-peaks from H_3_-20 to H-9 and H_3_-17 confirmed a similar spatial orientation of these protons and the absence of such a correlation between H_3_-20 and H-5, implied a similar configuration about the ring junction as observed in **1** (Figure 4a). The exact same chemical shift observed for H_3_-16 (δ_H_ 1.52, s) in compounds **1**–**3** indicated that all three labdane diterpenes have the same 13*S* configuration, consistent with the absolute configuration reported for the structurally related marine natural product, isoconncindiol [20,21]. The structure of **2** was therefore elucidated as the 8*S*-diastereomer of **1**, that is with the only difference observed at the stereogenic center C-8, thus confirming the structure of **2** as the 13-acetyl derivative of isoconcinndiol.

Compound **3** showed signals for five methyl singlets (δ_H_ 0.95; 0.96; 1.05; 1.52; 2.00), a methyl doublet (δ_H_ 0.86, d, 6.6 Hz, H_3_-17), several overlapping multiplets at δ_H_ 1.00–2.15, the vinyl protons double doublets (δ_H_ 5.12, dd, *J* = 0.7, 6.6 Hz, H-15a; 5.14, dd, *J* = 0.7, 13.1 Hz, H-15b; 5.90, dd, *J* = 11.0, 17.5 Hz, H-14) and the “halo-methine” proton H-3 at δ_H_ 4.00 (dd, *J* = 4.3, 12.4 Hz) in its ^1^H-NMR spectrum (Table 1). Four different ^1^H-^1^H COSY spin systems were observed (Figure 4b), with two of them (H_2_-1→H-3 and H-14→H_2_-15) similar to those also observed in compounds **1** and **2**. However, the presence of the significant H-5→H_3_-17 (Figure 4b), concomitant with the absence of the H-9→H_2_-12 COSY spin system and the splitting of H_3_-17 into a doublet, suggested that **3** was a positional isomer of **1** and **2**, with its hydroxyl group present at C-9 (δ_C_ 76.8) rather than C-8 (δ_C_ 35.8). The relative configuration of the groups on the cyclohexane ring A was retained whilst no ROE cross-peak was observed between H_3_-17 and H_3_-20. The structure of **3** was therefore elucidated as the 13-acetyl derivative of concinndiol [23].

### 2.2. Structural Elucidation of Polyether Triterpenes *(**4**–**7**)*

Compound **4** was isolated as a white amorphous solid. The ^1^H-NMR spectrum showed signals of eight methyl groups (δ_H_ 1.07, s; 1.10, s; 1.13, s; 1.16, s; 1.18, s; 1.19, s; 1.26, s; 1.38, s), several overlapping multiplicities at δ_H_ 1.3–2.3, and six methine proton groups (Table 2). The ^13^C-NMR spectrum showed 30 carbon signals and a high degree of oxygenation was inferred from 10 carbon resonances present in the downfield range δ_C_ 70–87 ppm. A signal observed at δ_C_ 59.0 suggested the presence of a bromo-methine C-3, characteristic in similar polyether triterpenes reported from the genus *Laurencia* [24,25]. This was confirmed by the presence of the mono-isotopic bromine peaks at *m*/*z* 611/609 [M + Na]^+^, 571/569 [M − OH]^+^ recorded in the LR-ESI-MS and consistent with the reported values by Cen-Pacheco and co-workers [17]. Compound **4** was deduced to be a congener of this group of secondary metabolites and the structure was found be that of the known compound saiyacenol B, previously isolated from *L. viridis* [17].

After careful examination of the 1D NMR spectra of a fraction obtained from the same HPLC run that yielded compound **2**, we identified the presence of a mixture of the two very closely related compounds **5** and **6**. However, due to the paucity of the HPLC fraction obtained (0.5 mg), we were unable to separate these two compounds any further. The HR-ESI-MS spectrum obtained for compound **5** established a molecular formula of C_30_H_50_O_6_, requiring six degrees of unsaturation. Its ^1^H-NMR spectrum exhibited signals of seven methyls (δ_H_ 1.08, s; 1.10, s; 1.13, s; 1.15, s; 1.19, s; 1.20, s; 1.68, s), several overlapping multiplicities at δ_H_ 1.2–2.2, six oxygenated methine protons double doublets and two olefinic geminal protons at δ_H_ 4.77 (m, H-1a) and 4.98 (m, H-1b) (Table 2). The ^13^C-NMR spectrum showed the presence of 30 non-equivalent carbons and the HSQC-DEPT confirmed the presence of seven methyl, 11 diastereotopic methylene, six methine and six quaternary carbons. Thorough analysis of the 1D and 2D NMR data identified compound **5** as another polyether triterpene, which lacked a C-3 bromine atom and one methyl group (δ_H_ 1.26, s, H_3_-1) compared to **4**. With evidence of only one double bond (C-1, δ_H_ 4.77/4.98, δ_C_ 110.2; C-2, δ_C_ 146.2), compound **5** was deduced to be pentacyclic.

**Figure 5:**
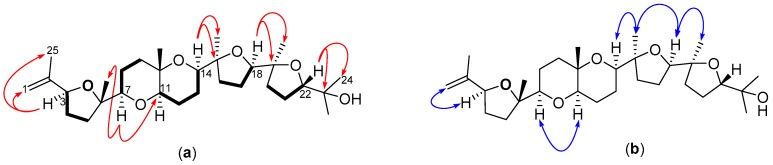
(**a**) COSY (**bold bonds**) and key HMBC (red arrows); and (**b**) key ROESY (blue arrows) of compound **5**.

Six ^1^H-^1^H COSY spin systems were observed, of which four exhibited the concomitant vicinal AA’BB’ coupling pattern involving two methylene groups, with one of the groups further involved in an ABX vicinal coupling with a methine proton (Figure 5a). The coupling constants recorded, especially for the methine protons supported the presence of both substituted oxolane and oxane moieties. After careful analysis of the long range ^1^H–^13^C correlations, the rest of the molecule was found to be similar to **4**, apart from the change in chemical shifts observed from C-1 through C-7 and C-25. An HMBC cross-peak between H-3 and C-1 and the downfield shift of C-3 to δ_C_ 83.4 suggested the absence of a bromine atom to give the exocyclic 2-methyl ethylenyl group on the oxolane ring in compound **5**, which is consistent with similar isolated compounds [26]. ROESY enhancements of H-14 and H-18 by H_3_-28 and H_3_-29, respectively, led to a similar relative configuration for **5** as reported for **4** [17] (Figure 5b). Compound **5** is reported for the first time here as Alfredensinol A.

The NMR data obtained for compound **6** were superimposable with that of **5**, signifying their structures were closely related (Figure 1), with the exception of the olefinic geminal protons of C-1 resonating downfield at δ_H_ 5.23 (m, H-1a) and δ_H_ 5.30 (m, H-1b) due to the presence of the hydroxyl group on the methylene carbon C-25 (δ_C_ 44.7). Moreover, C-1 occurred downfield at δ_C_ 114.7, while C-3 moved upfield to δ_C_ 80.7 (Table 2). The HR-ESI-MS ion peak at *m*/*z* 545.3457 [M + Na]^+^ (calcd. 545.3454) obtained for **6** established a molecular formula of C_30_H_50_O_7_, implying an additional O-atom compared to **5**. Therefore, compound **6** is reported here as the new C-25 hydroxyl derivative of compound **5**, Alfredensinol B.

**Figure 6:**
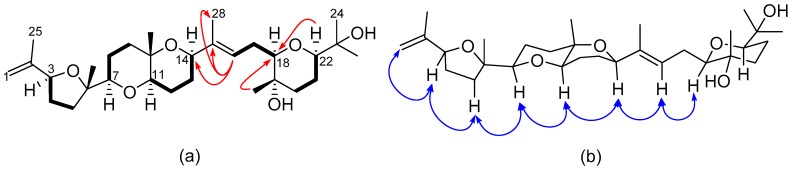
(**a**) COSY (bold bonds) and key HMBC (red arrows); and (**b**) some ROESY (blue arrows) correlations for compound **7**.

Compound **7** was isolated as a white amorphous solid. It recorded a HR-ESI-MS ion peak of *m*/*z* 507.3693 [M + H]^+^ (calcd. 507.3686) for the molecular formula C_30_H_50_O_6_ and was identified as another polyether triterpene from its ^1^H- and ^13^C-NMR spectra. Six oxygenated methine doublet of doublets including an olefinic proton (δ_H_ 5.52, dd, *J* = 2.7, 13.8 Hz, H-16) and similar olefinic geminal protons (δ_H_ 4.77, m, H-1a; 4.98, m, H-1b) to compound **5** (Table 2) were evident in the ^1^H-NMR spectrum. With six degrees of unsaturation inferred from the molecular formula, compound **7** was deduced to be a tetracyclic molecule with the presence of two double bonds. Six ^1^H-^1^H COSY spin systems were observed, including the key correlations involving the olefinic proton H-16, the diastereotopic methylene protons H_2_-17 and the oxymethine H-18 (Figure 6a). Key HMBC cross-peaks observed from H-16 to C-14, C-15, C-18, and C-28 established the Δ^15,16^ olefinic functionality. HMBC correlations from H-22 (^3^*J*_C, H_) and H_3_-29 (^3^*J*_C, H_) to C-18, along with key COSY correlations between H_2_-20, H_2_-21 and H-22, confirmed the presence of an oxane ring in the right hand side chain of **7**.

The absence of ROE enhancement of H-16 by H_3_-28 was indicative of the trans configuration about the double bond between C-15 and C-16, which was further supported by the ROESY correlation between H-14 and H-16 (Figure 6b). The relative configuration at C-18 was established as a result of the observed ROESY cross-peak from H-18 to H-16 and the absence of one to H_3_-29. The absence of an ROE correlation between H-22 and H_3_-29, implied axial orientations for both these groups on the oxane ring. Thus, the structure of **7** was elucidated and identified as a new compound which is named here as Alfredensinol C.

### 2.3. Structural Elucidation of Cholestane-Type Ecdysteroids *(**8**–**10**)*

Compound **8** was isolated as a white amorphous solid and HR-ESI-MS data obtained established the molecular formula as C_31_H_48_O_6_. The ^1^H-NMR spectrum showed the presence of seven methyl singlets of which two were acetate methyls at δ_H_ 2.03 (s, H_3_-30) and δ_H_ 2.04 (s, H_3_-31), and five oxymethine protons. Several overlapping multiplets were observed in the methylene envelope between δ_H_ 1.11–2.15 (Table 3). The ^13^C-NMR data showed 31 signals including three carbonyl peaks characteristic of a ketone at δ_C_ 199.6 (C-6) and two esters (δ_C_ 169.3, C-28; 169.5, C-29), two aromatic/olefinic carbons (δ_C_ 123.3, C-7; 162.5, C-8), and three signals at δ_C_ 68.5 (C-2), 68.3 (C-3), and 73.7 (C-22) suggesting the presence of C-O chemical environments (Table 3).

**Figure 7:**
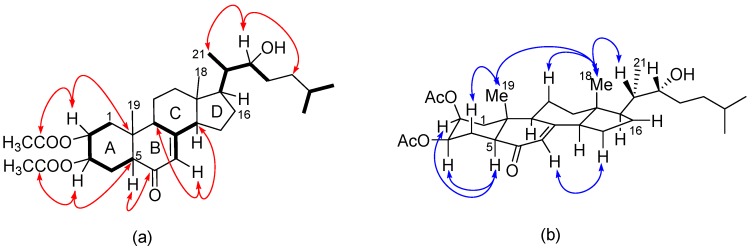
(**a**) Key COSY (bold bonds) and HMBC (red arrows); and (**b**) key NOESY (blue arrows) of **8**.

The HSQC-DEPT data indicated the presence of seven methyl, eight diastereotopic methylene, 10 methine, and six quarternary carbons. Two ^1^H-^1^H COSY spin systems were observed, from H_2_-1 to H-5 and H-7 to H_3_-27 (Figure 7a). Some characteristic aspects of a steroidal skeleton became evident from the ^1^H, ^13^C and COSY NMR data; for example, the ^1^H chemical shifts for H_3_-18 and H_3_-19, and the splitting of H_3_-21, H_3_-26 and H_3_-27 into doublets were characteristic of the side chain to C-17 for cholesterol. The chemical shifts of C-7 and C-8, together with the chemical shift of H-7 (δ_H_ 5.75, m) and its COSY correlation to H-9 (δ_H_ 2.25, ddd, *J* = 2.5, 6.9, 9.9) and H-14 (δ_H_ 2.05, m) suggested the ketone carbonyl was adjacent to the C-7-C-8 double bond at C-6 to give the endocyclic α, β-unsaturated ketone, a structural feature of ecdysteroids. HMBC cross-peaks from H_2_-1 to C-2, C-5, C-9, C-10 and C-19; H_2_-4 to C-2 and C-3; and H-5 to C-4, C-6, C-9, C-10 and C-19 confirmed the structure of ring A, and that the ketone was at C-6 (Figure 7a). Similarly, correlations from H-7 to C-9 and C-14; H-9 to C-7, C-8, C-11 and C-19; and from H-14 to C-7, C-8, C-12, C-13, C-15 and C-18 confirmed the structures of rings B, C and D, respectively. The single ^1^H-^1^H COSY spin system from H-17 to H-27 was supported by the HMBC cross-peaks to arrive at the 2-hydroxy-1, 5-dimethylhexyl side chain at C-17, and hence the position of the hydroxyl group at C-22. Finally, COSY correlation between H-2 and H-3 and HMBC correlation of H-2 and H-3 to C-28 and C-29, respectively, signified the acetates were attached to the cholestane ecdysteroid backbone at C-2 and C-3.

Key NOESY enhancements together with ^13^C comparisons enabled the assignment of the relative configuration of **8** (Figure 7b). The chair conformation and trans-fused A/B ring junction were established from two 1,3-diaxial NOESY correlations from H_3_-19β to H_3_-30β (OAc) and H-4β (δ_H_ 1.93) respectively, and a further two 1,3-diaxial NOESY correlations from H-5 to H-3 and H-9, where the latter three protons are all in the same α-orientation. The trans-ring-fusion between rings A and B was further corroborated by the absence of a NOESY cross peak between the methine proton H-5 and H_3_-19. The presence of correlation between H_3_-18 and H_3_-19 but not H_3_-21 established the β-configuration of H_3_-18 and H_3_-19 to the steroid backbone suggesting a chair-chair-chair conformation of rings A, B and C. The relative configurations at C-17, C-20, C-21 and C-22 were assigned, by comparison of the ^13^C-NMR data with that of the structurally related 22*R* hydroxyl cholesterol [27]. The structure of **8** was determined as a new compound and named Alfredensterol.

Compound **9** was isolated as a white amorphous solid and its molecular formula was found to be C_31_H_48_O_7_ from the HR-ESI-MS ion peak at *m*/*z* 533.3488 (calcd. 533.3478). The ^1^H- and ^13^C-NMR spectroscopic data recorded for compound **9** were virtually identical to compound **8** and suggested a closely related cholestane-type ecdysteroid (Table 3). The two acetate methyl singlets (δ_H_ 2.04, s, H_3_-30; δ_H_ 2.05, s, H_3_-31), together with the signals for H_3_-19, H_3_-26 and H_3_-27 for **9** were in close correspondence (±0.02 ppm) with the chemical shifts for **8**, while a downfield shift to δ_H_ 0.76 was observed for H_3_-18. The HSQC-DEPT spectrum revealed one less methine proton and one additional quaternary carbon at δ_C_ 96.1, indicating the likely presence of a tertiary alcohol functionality at C-14 and therefore accounting for the extra O-atom in the molecular formula of **9**. Moreover, the ^1^H-^1^H COSY spectrum exhibited three spin systems from H_2_-1 to H-5, H-7 to H_2_-12 and H_2_-15 to H_3_-27, thus revealing the absence of H-14. The relative configuration of compound **9** was assigned primarily using ^13^C and ROESY NMR data, and were found to be similar to those observed in **8**. To the best of our knowledge, there is only one report of **9** in the literature as a by-product in the synthesis of ecdysone related compounds, in which no extensive NMR assignments could be found, including ^13^C-NMR data [18]. Therefore, this is the first report of **9** from a natural source, which we have named 14α-hydroxy Alfredensterol.

Compound **10** was isolated as a white amorphous solid. It recorded a HR-ESI-MS ion peak of *m*/*z* 475.3432 [M + H]^+^ (calcd. 475.3423) for the molecular formula C_29_H_46_O_5_. The ^1^H- and ^13^C-NMR data for **10** were very similar to both compounds **8** and **9** described above. However, key noticeable differences in the ^1^H-NMR data of **10** were observed, including the absence of an acetate methyl singlet and the upfield chemical shifts of H-2 (δ_H_ 4.88, m) and H-3 (δ_H_ 4.01, m) compared to **8**. The ^13^C-NMR data of **10** showed corresponding changes (δ_C_ 71.3, C-2; 66.4, C-3) consistent with its ^1^H-NMR. The ^1^H-^1^H COSY spin systems were similarly consistent with those observed in compound **8**. The position of the single acetate group on ring A was assigned to C-2, on the basis of the chemical shifts of C-2 and C-3 (Table 3), and HMBC cross-peaks from H-2 to C-3, C-4, C-10 and C-28, and from H-3 to C-1, C-2 and C-5. Analysis of the ROESY data revealed the same relative configuration of **10** as observed in **8** and **9**. Compound **10** has not previously been reported and is named here as 3-deacetoxy Alfredensterol.

### 2.4. Characterization of Isolated Glycolipid *(**11**)*

**Figure 8:**
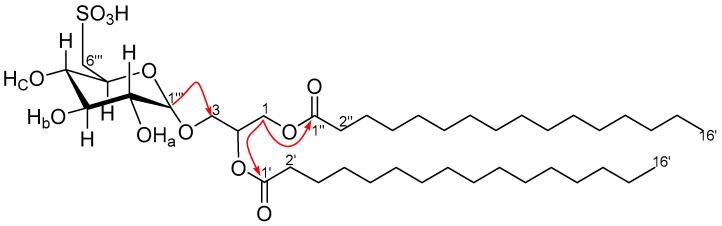
Key HMBC (red arrows) **11**.

Compound **11** (Figure 8) was isolated as a white amorphous solid. The HR-ESI-MS data of **11** suggested a molecular formula of C_41_H_78_O_12_S for *m*/*z* 793.5143 [M − H]^−^ (calcd. 793.5136). The structure was elucidated upon analyses of its NMR data and confirmed with reported data as 1, 2-di-*O*-palmitoyl-3-*O*-(6-sulfo-α-d-quinovopyranosyl)-glycerol [19]. This is the first report of **11** from the genus *Laurencia*.

### 2.5. Antiproliferative Activity Results

**Figure 9:**
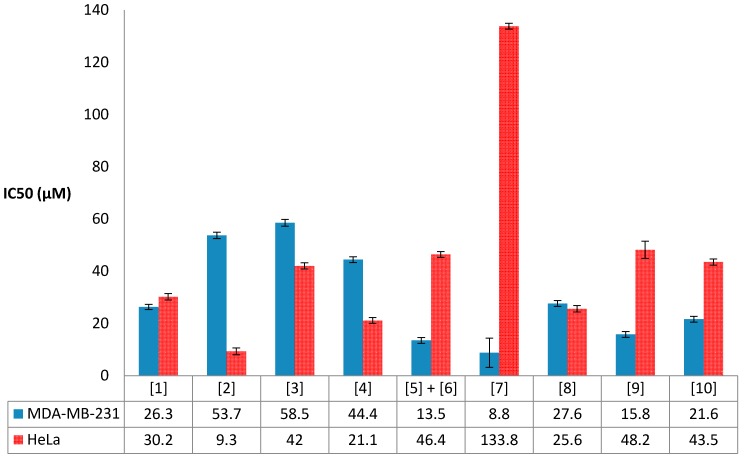
Results from the antiproliferative activity evaluation of compounds **1**–**10** against human breast (MDA-MB-231) and cervical (HeLa) cancer cell lines. (The standard errors of the mean IC_50_ values and correlation coefficients can be found in Supplementary Materials, Table S1).

Compounds **1**–**11** were evaluated for their antiproliferative activity against MDA-MB-231 triple negative human breast carcinoma and HeLa human cervical carcinoma (Figure 9). Generally, all the compounds displayed antiproliferative activity in the mid-to-low micromolar range against the two cells lines tested.

To the best of our knowledge, brominated labdane diterpenes previously isolated from the genus *Laurencia* have not been investigated for their antiproliferative activity. However, non-halogenated labdanes have been reported to exhibit moderate to weak cytotoxic activity against various cancer cell lines [28,29,30]. In this study, the brominated labdane-type diterpenes **1**–**3** were all found to be moderately active against both cancer cell lines tested, with the exception of Isoconcinndiol 13-acetate **2**, the 8*S*-diastereomer of **1**, which showed the highest antiproliferative activity against the HeLa cancer cell line (IC_50_ 9.3 ± 1.3 µM), not only in this class of compounds but amongst all ten compounds tested in this study (Figure 9), indicating that the stereochemistry at C-8 in **2** could be playing a role in the observed biological activity.

Brominated polyether triterpenes of the [4.4.0] class have been reported to have good to moderate cytotoxic activity [31]. The antiproliferative activity against the HeLa cell line recorded for the known compound **4**, saiyacenol B, in our study was found to be within the previously reported values [17]. To the best of our knowledge, this current work is the first report of the antiproliferative effects of this class of polyether triterpenes against the MDA-MB-231 breast cancer cell line, with the new compound **7**, alfredensinol C, exhibiting the highest activity (IC_50_ 8.8 ± 5.6 µM) in this particular class of compounds and also amongst all compounds tested (Figure 9).

The antiproliferative activity recorded for the ecdysteroids **8**–**10** was consistent with biological activity data previously reported for both natural and semi-synthetic congeners which have been shown to exhibit moderate antiproliferative or cytotoxic activity [32]. In this particular class of compounds, the new alfredensterol **8** was found to be the most active against the HeLa cervical cancer cell line (IC_50_ 25.6 ± 1.2 µM), whilst its 14α-hydroxyl derivative **9** exhibited the best antiproliferative effect (IC_50_ 15.8 ± 1.1 µM) against the MDA-MB-231 breast cancer cell line (Figure 9).

In summary, the new compounds **2** and **7** exhibited the best antiproliferative activity against the HeLa and MDA-MB-231 cancer cell lines, respectively, while **11** was found to be non-toxic to either cell line.

## 3. Materials and Methods

### 3.1. General Experimental Procedures

Melting points (uncorrected) were recorded on a Reichert-Jung Thermovar hot-stage microscope (Reichert Optische Werke, Vienna, Austria). Optical rotations were measured on a PerkinElmer141 polarimeter by using Na lamp (PerkinElmer Ltd., Beaconsfield, Buckinghamshire, UK. UV spectra were obtained on a CARY 60 UV-VIS 2.00 with software version 5.0.0.999 by Agilent Technologies (Santa Clara, CA, USA). PerkinElmer Spectrum Version 10.03.02 (PerkinElmer Ltd., Llantrisant, Wales, UK) was used to record the IR spectra. NMR spectra were obtained on a BRUKER Ascend 600 ((Bruker, Billeria, MA, USA) cryoprobe prodigy at 600 MHz and 150 MHz for ^1^H and ^13^C nuclei, respectively. CDCl_3_ (δ_H_ 7.25, δ_C_ 77.00) was used for **1**–**10** while DMSO-*d*_6_ (δ_H_ 2.50, δ_C_ 49.00) was used for **11**. HR-ESI-MS data were obtained via LC-TOF-MS on a Waters Synapt G2 (Waters Corp, Boston, MA, USA), ESI probe injected into a stream of acetonitrile, Cone voltage 15 V. Normal phase HPLC was carried out on Agilent 1220LC system/1260 Infinity (Santa Clara, CA, USA) equipped with a photodiode array and refractive index detectors. Column chromatography was carried out on silica gel 60 (Fluka 70–230 mesh, 63–200 μm, (Sigma-Aldrich, Buchs, Switzerland), and preparative TLC on silica gel 60 Analtech GF_254_ (20 × 20 cm, 2000 μm, Analtech Inc., Newark, DE, USA). Analytical TLC were performed on Merck silica gel 60 F_254_ (Merck KGaA, Darmstadt, Germany) and silica gel 60 RP-18 F_254_ plates and bands were visualized by heating after staining with ceric ammonium sulfate reagent.

### 3.2. Plant Material

The species studied here has only recently been described [33]. It was previously known in South Africa as *Laurencia elata* (C. Agardh) Hooker & Harvey. The latter is an Australian species, which has recently been placed in a new genus as *Coronaphycus elatus* (C. Agardh) Metti [34]. The South African material has been described as a new endemic species *Laurencia alfredensis* [33]. It is the same material previously described as “*Laurencia* cf. *elata*”, and used in ^1^H-NMR profiling of crude organic extracts as an identification tool for nine species of South African *Laurencia* [16].

### 3.3. Extraction, Isolation, and Characterization

The fresh alga (239.2 g) was extracted sequentially with MeOH (15 min) and then CH_2_Cl_2_/MeOH (2:1 *v*/*v*) (24 h) by cold maceration at room temperature. The solvents were evaporated in vacuo giving 5.1 g and 1.1 g of dark green MeOH and CH_2_Cl_2_/MeOH (2:1 *v*/*v*) crude extracts, respectively. The two extracts were combined based on close similarity of their ^1^H-NMR data. The resultant extract was triturated with CH_2_Cl_2_/MeOH (1:1 *v*/*v*) to obtain 1.5 g of organic fraction and 4.7 g of aqueous fraction. Fractionation by flash chromatography was carried out on 1 g of the organic fraction with n-hexane and EtOAc mixtures of increasing polarity and finally with EtOAc/MeOH (1:1 *v*/*v*). The fractions obtained with 80%, 70% and 40% *n*-hexane in EtOAc gave similar TLC profiles and were combined and further chromatographed on a silica column with *n*-hexane/EtOAc (9:1 *v*/*v*) with increasing polarity to *n*-hexane/EtOAc (4:6 *v*/*v*) to afford 12 sub-fractions **A**–**L**. Preparative TLC (*n*-hexane/EtOAc 8.5:1.5 *v*/*v*) of **B** gave **3** (1.1 mg) while **1** (3.7 mg) and **4** (4.8 mg) were obtained with *n*-hexane/EtOAc (3:1 *v*/*v*) from **E** and **D**, respectively. Compounds **8** (3.1 mg) and **9** (3.5 mg) were obtained from **I** with *n*-hexane/EtOAc (1:1 *v*/*v*) and **10** (1.9 mg) from **J** with the same mobile phase. Further purification on Phenomenex Luna 10 μm Prep Silica (2) 250 mm × 10 mm (Phenomenex, Torrance, CA, USA) of **F** with *n*-hexane/EtOAc (4:1 *v*/*v*) at a flow rate of 4 mL/min gave **2** (0.5 mg) and 0.5 mg of a mixture of **5** and **6**. Compound **7** (2.5 mg) was obtained on a Whatman Partisil column 10 μm, 500 mm × 10 mm (Hichrom Ltd., Reading, Berkshire, UK) from **K** and **L** with *n*-hexane/EtOAc (3:2 *v*/*v*) eluting at 4 mL/min, followed by reversed phase preparative TLC with MeOH/H_2_O (1:4 *v*/*v*). Meanwhile, the EtOAc/MeOH (1:1 *v*/*v*) fraction precipitated out a solid which on trituration with CH_2_Cl_2_ afforded **11** (49.6 mg).

*13-Acetyl Pinnatol A* (**1**): Clear crystals; m.p.: 115–117 °C; ${\left[\alpha \right]}_{D}^{20}$ +26.6 (*c* 0.15, CHCl_3_); IR (cm^−1^): 3450, 2950, 2850, 1700, 1350, 1250, 1100; ^1^H- and ^13^C-NMR data (CDCl_3_), Table 1; HR-ESI-MS *m*/*z* 451.1824 [M + Na]^+^ (calcd. for C_22_H_37_O_3_^79^BrNa, 451.1824).

*Isoconcinndiol 13-acetate* (**2**): White amorphous solid; ${\left[\alpha \right]}_{D}^{20}$ +14.4 (*c* 0.09, CHCl_3_); IR (cm^−1^): 3455, 2943, 1732, 1367, 1251, 1095; ^1^H- and ^13^C-NMR data (CDCl_3_), Table 1; HR-ESI-MS *m*/*z* 451.1821 [M + Na]^+^ (calcd. for C_22_H_37_O_3_^79^BrNa, 451.1824).

*Concinndiol 13-acetate* (**3**): White amorphous solid; ${\left[\alpha \right]}_{D}^{20}$ +16.7 (*c* 0.06, CHCl_3_); IR (cm^−1^): 3467, 2923, 1732, 1462, 1367, 1241; ^1^H- and ^13^C-NMR data (CDCl_3_), Table 1; HR-ESI-MS *m*/*z* 451.1821 [M + Na]^+^ (calcd. for C_22_H_37_O_3_^79^BrNa, 451.1824).

*Saiyacenol B* (**4**) [17]: White amorphous solid; ^1^H- and ^13^C-NMR data (CDCl_3_), Table 2; ESI-MS *m*/*z* 611, 609, 606, 604, 571, 569 and 507.

*Alfredensinol A* (**5**): Clear oil; ^1^H- and ^13^C-NMR data (CDCl_3_), Table 2; HR-ESI-MS *m*/*z* 505.3527 [M − H]^+^ (calcd. for C_30_H_49_O_6_, 505.3529).

*Alfredensinol B* (**6**): Clear oil; ^1^H- and ^13^C-NMR data (CDCl_3_), Table 2; HR-ESI-MS *m*/*z* 545.3457 [M + Na]^+^ (calcd. for C_30_H_51_O_7_Na, 545.3454).

*Alfredensinol C* (**7**): White amorphous solid; ${\left[\alpha \right]}_{D}^{20}$ +15.0 (*c* 0.25, CHCl_3_); IR (cm^−1^): 3409, 2940, 1374, 1092; ^1^H- and ^13^C-NMR data (CDCl_3_), Table 2; HR-ESI-MS *m*/*z* 507.3693 [M + H]^+^ (calcd. for C_30_H_51_O_6_, 507.3686).

*Alfredensterol* (**8**): White amorphous solid; ${\left[\alpha \right]}_{D}^{20}$ +22.0 (*c* 0.13, CHCl_3_); UV (MeOH) λ_max_(log ε), nm: 239 (3.43); IR (cm^−1^): 3425, 2925, 1737, 1643, 1350, 1234, 1035; ^1^H- and ^13^C-NMR data (CDCl_3_), Table 3; HR-ESI-MS *m*/*z* 517.3527 [M + H]^+^ (calcd. for C_31_H_49_O_6_, 517.3529).

*14α-Hydroxy Alfredensterol* (**9**): White amorphous solid; ${\left[\alpha \right]}_{D}^{20}$ +25.8 (*c* 0.26, CHCl_3_); UV (MeOH) λ_max_(log ε), nm: 222 (3.76); IR (cm^−1^): 3240, 2925, 1738, 1645, 1375, 1234, 1036; ^1^H- and ^13^C-NMR data (CDCl_3_), Table 3; HR-ESI-MS *m*/*z* 533.3488 [M + H]^+^ (calcd. for C_31_H_49_O_7_, 533.3478).

*3-Deacetoxy Alfredensterol* (**10**): White amorphous solid; ${\left[\alpha \right]}_{D}^{20}$ +24.0 (*c* 0.30, CHCl_3_); UV (MeOH) λ_max_(log ε), nm: 231 (3.27); IR (cm^−1^): 3397, 2948, 1715, 1660, 1325, 1240, 1030; ^1^H- and ^13^C-NMR data (CDCl_3_), Table 3; HR-ESI-MS *m*/*z* 475.3432 [M + H]^+^ (calcd. for C_29_H_47_O_5_, 475.3423).

*1,2-di-O-Palmitoyl-3-O-(6-sulfo-α-d-quinovopyranosyl)-glycerol* (**11**) [19]: White amorphous solid; ^1^H-NMR (600 MHz, DMSO-*d*_6_): δ 4.13 (1H, dd, *J* = 7.3, 12.0, H-1a), 4.33 (1H, dd, *J* = 2.8, 11.9, H-1b), 5.12 (1H, m, H-2), 3.40 (1H, m, H-3a), 3.88 (1H, dd, *J* = 5.9, 10.4 Hz, H-3b), 2.28 (4H, m, H-2′ and 2″), 1.45–1.57 (4H, m, H-3′ and 3″), 1.19–1.31 (48H, m, H-4′–15′ and 4″–15″), 0.85 (6H, t, *J* = 6.8 Hz, H-16′ and 16″), 4.57 (1H, d, *J* = 2.4 Hz, H-1′′′), 3.19 (1H, m, H-2′′′), 3.35 (1H, m, H-3′′′), 2.94 (1H, dt, *J* = 4.5, 9.1 Hz, H-4′′′), 3.77 (1H, dt, *J* = 6.2, 10.3 Hz, H-5′′′), 2.56 (1H, dd, *J* = 6.2, 13.9 Hz, H-6′′′a), 2.88 (1H, dd, *J* = 4.8, 13.9 Hz, H-6′′′b), 4.58 (1H, d, *J* = 5.1 Hz, OH-2′′′), 4.65 (1H, d, *J* = 4.6 Hz, OH-3′′′), 5.39 (1H, d, *J* = 3.6 Hz, OH-4′′′); ^13^C-NMR (600 MHz, DMSO-d_6_): δ 62.5 (C-1), 69.6 (C-2), 64.6 (C-3), 172.2 (C-1′), 172.4 (C-1″), 33.3 (C-2′), 33.5 (C-2″), 24.3 (C-3′ and 3″), 28.3–28.9 (C-4′–15′ and 4″–15″), 13.8 (C-16′ and 16″), 98.3 (C-1′′′), 71.5 (C-2′′′), 72.8 (C-3′′′), 74.3 (C-4′′′), 68.4 (C-5′′′), 54.5 (C-6′′′); HR-ESI-MS *m*/*z* 793.5143 [M − H]^−^ (calcd. for C_41_H_77_O_12_S, 793.5136).

### 3.4. X-ray Crystallographic Data

The crystal for compound **1** was obtained by recrystallization from methanol and its structure was solved by direct methods from intensity data collected from the crystal specimen at 173(2) K on a Bruker Apex II Duo diffractometer and refined by full-matrix least-squares. The Flack x parameter value of −0.013(6), indicating that the correct absolute configuration had been assigned, was determined using 1652 quotients [(I+) − (I−)]/[(I+) + (I−)]. Salient crystallographic data for **1** are as follows:

Crystal Data for C_22_H_37_O_3_Br (M = 429.42 g/mol): monoclinic, space group P2_1_ (no. 4), a = 11.1926(11) Å, *b* = 7.2138(7) Å, *c* = 14.8442(13) Å, β = 111.571(2)°, V = 1114.60(18) Å^3^, Z = 2, T = 173(2) K, μ(MoKα) = 1.861 mm^−1^, D_calc_ = 1.280 g cm^−3^, 20,619 reflections measured (2.95° ≤ 2θ ≤ 56.02°), 5386 unique (R_int_ = 0.0549, Rsigma = 0.0537) which were used in all calculations. The final R_1_ was 0.0378 (I > 2σ(I)) and wR_2_ was 0.0760 (all data).

Further technical details of the structure determination and molecular parameters are provided in the CIF file (Supplementary Materials).

### 3.5. Cell Culture and Antiproliferative Activity Assay

MDA-MB-231 breast cancer cells (ATCC HTB-26) were maintained in culture in phenol-red free L-15 medium supplemented with 10% (*v*/*v*) heat-inactivated fetal bovine serum (FBS), 1 mM l-glutamine, 100 U/mL penicillin, 100 μg/mL streptomycin and 12.5 μg/mL amphotericin (PSA) at 37 °C in a humidified incubator. HeLa cervical cancer cells (ATCC CCL-2) were cultured in Dulbecco’s Modified Eagle Medium (DMEM) supplemented as above at 37 °C and 9% CO_2_ in a humidified incubator.

The antiproliferative effects of the compounds were assessed using the WST-1 assay (Sigma-Aldrich, Johannesburg, South Africa) as previously described [35,36]. Briefly, cells were seeded at a density of 6000 cells per well into 96-well plates and incubated overnight, followed by treatment with a range of concentrations (0.32, 1.6, 8, 40, 200 and 1000 μM) of the compounds or dimethyl sulfoxide (DMSO) vehicle control (0.02% *v*/*v* DMSO) for 96 h. Thereafter 2.5 µL of WST-1 Cell Proliferation Reagent was added per well and the absorbance at 450 nm after 8 h recorded using a Synergy Mx spectrophotometer (BioTek). The half maximal inhibitory concentration (IC_50_) for each compound was calculated relative to the vehicle-treated control from a dose response curve (log concentration vs absorbance at 595 nm) using non-linear regression with GraphPad Prism 4 (GraphPad Inc., San Diego, CA, USA).

## Figures and Tables

**Table 1:** ^1^H (600 MHz, CDCl_3_) and ^13^C-NMR (150 MHz, CDCl_3_) data for compounds **1**–**3**.

No.	1		2		3	
	δ_C_	δ_H_ (*J*, Hz)	δ_C_	δ_H_ (*J*, Hz)	δ_C_	δ_H_ (*J*, Hz)
**1a**		1.14, dt (3.5, 13.4)		1.15, m		1.42, m
**1b**	37.4, CH_2_	1.62, td (3.9, 12.9, 13.2)	37.3, CH_2_	1.71, m	33.5, CH_2_	1.71, m
**2a**		2.06, dq (3.9, 13.2)		2.09, m		2.12, m
**2b**	30.7, CH_2_	2.30, qd (3.9, 13.2)	30.9, CH_2_	2.25, qd (3.8, 13.1)	30.7, CH_2_	2.15, m
**3**	69.7, CH	3.92, dd (4.1, 12.8)	69.2, CH	3.93, dd (4.1, 12.7)	69.6, CH	4.00, dd (4.3, 12.4)
**4**	39.5, C		39.4, C		39.6, C	
**5**	47.6, CH	1.08, dd (2.5, 10.9)	47.1, CH	1.16, m	47.0, CH	1.64, dd (2.7, 12.3)
**6a**		1.50, m		1.40, m		1.39, m
**6b**	20.2, CH_2_	1.72, m	22.0, CH_2_	1.64, m	23.1, CH_2_	1.60, m
**7a**		1.46, m		1.46, m		1.29, m
**7b**	36.7, CH_2_	1.53, m	37.2, CH_2_	1.58, m	31.3, CH	1.46, m
**8**	74.7, C		73.1, C		35.9, CH	1.73, m
**9**	59.1, CH	0.84, m	60.9, CH	0.95, m	76.8, C	
**10**	39.0, C		38.7, C		43.3, C	
**11a**		1.00, m		1.28, m		1.40, m
**11b**	23.0, CH_2_	1.38, m	20.8, CH_2_	1.71, m	28.0, CH_2_	1.55, m
**12a**		1.74, m		1.79, m		1.74, m
**12b**	43.0, CH_2_	1.80, m	42.9, CH_2_	1.82, m	35.8, CH_2_	1.95, ddd, (5.1, 12.4, 13.7)
**13**	82.9, C		83.2, C		83.3, C	
**14**	141.6, CH	5.94, dd (11.0, 17.5)	141.8, CH	6.00, dd (11.0, 17.5)	141.6, CH	5.90, dd (11.0, 17.5)
**15a**		5.13, dd (0.9, 17.5)		5.13, dd (0.9, 17.5)		5.12, dd (0.7, 6.6)
**15b**	113.4, CH_2_	5.15, dd (0.9, 11.0)	113.2, CH_2_	5.15, dd (0.9, 11.0)	113.4, CH_2_	5.14, dd (0.7, 13.1)
**16**	23.5, CH_3_	1.52, s	23.7, CH_3_	1.52, s	23.7, CH_3_	1.52, s
**17**	30.9, CH_3_	1.20, s	31.8, CH_3_	1.43, s	16.0, CH_3_	0.82, d (6.6)
**18**	17.7, CH_3_	0.95, s	17.6, CH_3_	0.91, s	18.4, CH_3_	0.96, s
**19**	30.6, CH_3_	1.06, s	30.5, CH_3_	1.07, s	30.9, CH_3_	1.05, s
**20**	24.7, CH_3_	1.28, s	24.7, CH_3_	1.09, s	16.2, CH_3_	0.95, s
**21**	169.8, C		169.9, C		169.8, C	
**22**	22.1, CH_3_	2.00, s	22.2, CH_3_	2.00, s	22.1, CH_3_	2.00, s

**Table 2:** ^1^H- (600 MHz, CDCl_3_) and ^13^C-NMR (150 MHz, CDCl_3_) data for compounds **4**–**7**.

No.	4		5		6		7	
	δ_C_	δ_H_ (*J*, Hz)	δ_C_	δ_H_ (*J*, Hz)	δ_C_	δ_H_ (*J*, Hz)	δ_C_	δ_H_ (*J*, Hz)
**1a**				4.77, m		5.23, m		4.76, m
**1b**	31.0	1.26, s	110.2	4.98, m	114.7	5.30, m	110.2	4.97, m
**2**	74.9		146.2		146.0		145.9	
**3**	59.0	3.88, dd (4.0, 12.3)	83.4	4.35, dd (6.1, 8.9	80.7	4.53, dd (5.2, 9.5)	83.3	4.34, dd (6.1, 8.7)
**4a**		2.09, dt (4.0, 13.5)		1.71, m		1.83, m		1.71, m
**4b**	28.2	2.23, qd (3.7, 13.1)	31.4	2.02, m	32.3	2.15, m	31.3	2.01, m
**5a**		1.53, m		1.64, m		1.68, m		1.62, m
**5b**	37.4	1.80, m	34.3	2.12, m	34.1	1.81, m	34.2	2.10, m
**6**	74.4		84.5		84.5		84.4	
**7**	86.5	3.03, dd (2.3, 11.4)	83.6	3.32, dd (2.6, 11.6)	83.6	3.31, dd (2.6, 11.6)	83.6	3.34, dd (2.7, 11.6)
**8a**		1.40, m		1.44, m		1.44, m		1.45, m
**8b**	23.0	1.70, m	25.0	1.64, m	25.0	1.64, m	24.8	1.65, m
**9a**		1.52, m		1.57, m		1.57, m		1.56, m
**9b**	38.7	1.73, m	38.8	1.76, m	38.7	1.76, m	38.8	1.78, m
**10**	71.4		71.3		71.2		72.1	
**11**	76.7	3.53, dd (7.3, 11.0)	76.6	3.58, dd (7.3, 11.0)	76.6	3.58, dd (7.3, 11.0)	77.8	3.51, dd (6.5, 11.2)
**12a**		1.48, m		1.52, m		1.52, m		1.57, m
**12b**	21.3	1.86, m	21.4	1.93, m	21.4	1.93, m	21.7	1.88, m
**13a**								1.70, m
**13b**	21.4	1.77	21.5	1.78, m	21.5	1.78, m	25.8	2.02, m
**14**	75.3	3.71, dd (4.3, 11.0)	75.4	3.71, dd (4.8, 11.2)	75.5	3.72, dd (4.8, 11.2)	75.1	4.19, dd (4.0, 9.5)
**15**	84.5		84.4		84.4		138.5	
**16a**		1.61, m		1.64, m		1.64, m		
**16b**	35.5	1.95, m	35.4	1.96, m	35.4	1.96, m	122.3	5.52, dd (2.7, 13.8)
**17a**		1.63, m		1.66, m		1.66, m		2.06, m
**17b**	27.6	1.84, m	27.7	1.86, m	27.7	1.86, m	27.6	2.40, ddd (3.1, 7.6, 15.1)
**18**	85.8	3.85, dd (6.1, 8.4)	85.8	3.86, dd (5.8, 8.2)	85.8	3.86, dd (5.8, 8.2)	84.0	3.16, dd (3.2, 6.7)
**19**	84.5		84.6		84.6		69.9	
**20a**		1.59, m						1.56, m
**20b**	33.9	1.96, m	34.0	1.96, m	34.0	1.96, m	39.7	1.84, m
**21a**								1.49, m
**21b**	26.5	1.79, m	26.6	1.80, m	26.6	1.80, m	24.5	1.60, m
**22**	86.8	3.76, dd (6.8, 8.6)	86.8	3.77, dd (6.7, 8.7)	86.8	3.77, dd (6.7, 8.7)	84.1	3.14, m
**23**	70.6		70.6		70.6		71.6	
**24**	24.0	1.10, s	24.0	1.10, s	24.0	1.10, s	23.9	1.12, s
**25**	23.7	1.38, s	17.7	1.68, s	44.7	4.09, d (0.9)	17.7	1.68, s
**26**	20.0	1.19, s	23.2	1.15, s	23.3	1.17, s	23.1	1.16, s
**27**	21.2	1.16, s	21.3	1.20, s	21.3	1.20, s	20.1	1.21, s
**28**	21.5	1.07, s	21.6	1.08, s	21.6	1.08, s	13.0	1.65, s
**29**	23.6	1.13, s	23.6	1.13, s	23.6	1.13, s	20.2	1.18, s
**30**	27.5	1.18, s	27.6	1.19, s	27.6	1.19, s	26.1	1.16, s

**Table 3:** ^1^H- (600 MHz, CDCl_3_) and ^13^C-NMR (150 MHz, CDCl_3_) data for compounds **8**–**10**.

No.	8		9		10	
	δ_C_	δ_H_ (*J*, Hz)	δ_C_	δ_H_ (*J*, Hz)	δ_C_	δ_H_ (*J*, Hz)
**1a**		1.72, m		1.80, dd (3.4, 15.0)		
**1b**	36.7, CH_2_	2.00, m	36.6, CH_2_	2.01, m	36.1, CH_2_	1.88, m
**2**	68.5, CH	4.93, m	68.5, CH	4.93, m	71.3, CH	4.88, m
**3**	68.5, CH	5.04, m	68.3, CH	5.05, m	66.4, CH	4.01, m
**4a**		1.93, ddd (2.8, 12.6, 15.4)		1.94, m		1.87, m
**4b**	21.2, CH_2_	2.10, m	21.1, CH_2_	2.10, m	23.6, CH_2_	2.03, m
**5**	48.7, CH	2.58, dd (3.5, 12.5)	49.0, CH	2.63, dd (3.23, 12.2)	47.8, CH	2.74, dd (3.5, 12.4)
**6**	199.6, C		199.4, C		200.6, C	
**7**	123.3, CH	5.75, m	127.0, CH	5.93, d, (2.2)	123.3, CH	5.73, m
**8**	162.5, C		157.6, C		162.6, C	
**9**	50.6, CH	2.25, ddd (2.5, 6.9, 9.9)	46.8, CH	2.68, m	50.7, CH	2.25, ddd (2.5, 6.7, 11.7)
**10**	37.7, C		38.0, C		37.9, C	
**11a**		1.62, m		1.58, m		1.61, m
**11b**	21.5, CH_2_	1.78, m	20.3, CH_2_	1.76, m	21.5, CH_2_	1.79, m
**12a**		1.40, m		1.69, m		1.41, m
**12b**	38.7, CH_2_	2.14, m	29.9, CH_2_	1.94, m	38.7, CH_2_	2.12, dd (1.9, 13.0)
**13**	44.8, C		48.2, C		44.8, C	
**14**	55.1, CH	2.05, m	96.1, C		55.1, CH	2.05, m
**15a**		1.53, m		1.72, m		1.53, m
**15b**	22.6, CH_2_	1.64, m	24.7, CH_2_	2.15, m	22.6, CH_2_	1.65, m
**16a**		1.44, m		1.52, m		1.44, m
**16b**	26.9, CH_2_	1.80, m	25.7, CH_2_	1.89, m	26.9, CH_2_	1.82, m
**17**	53.3, CH	1.33, m	47.9, CH	1.89, m	53.2, CH	1.34, m
**18**	12.3, CH_3_	0.61, s	16.4, CH_3_	0.76, s	12.3, CH_3_	0.60, s
**19**	14.9, CH_3_	0.96, s	14.7, CH_3_	0.97, s	14.8, CH_3_	0.96, s
**20**	42.4, CH	1.68, m	41.8, CH	1.74, m	42.4, CH	1.69, m
**21**	12.6, CH_3_	0.94, d (6.8)	12.8, CH_3_	0.89, d (5.1)	12.6, CH_3_	0.94, d (6.4)
**22**	73.7, CH	3.62, dd (1.6, 10.1)	74.1, CH	3.63, m	73.8, CH	3.61, m
**23a**		1.24, m		1.23, m		1.25, m
**23b**	27.8, CH_2_	1.36, m	27.2, CH_2_	1.39, m	27.7, CH_2_	1.36, m
**24a**		1.17, m		1.17, m		1.16, m
**24b**	36.0, CH_2_	1.39, m	36.0, CH_2_	1.39, m	36.0, CH_2_	1.42, m
**25**	28.1, CH	1.55, m	28.2, CH	1.56, m	28.1, CH	1.55, m
**26**	22.4, CH_3_	0.89, d (6.8)	22.4, CH_3_	0.90, d (6.4)	22.4, CH_3_	0.89, d (6.9)
**27**	22.9, CH_3_	0.90, d (6.8)	23.0, CH_3_	0.92, d (6.4)	22.9, CH_3_	0.90, d (6.9)
**28**	169.3, C		169.4, C		170.0, C	
**29**	169.5, C		169.6, C		21.2, CH_3_	2.02, s
**30**	21.1, CH_3_	2.03, s	21.2, CH_3_	2.04, s		
**31**	21.2, CH_3_	2.04, s	21.2, CH_3_	2.05, s		

## Supplementary Materials

Supplementary materials are available online. The IR, HR-MS, 1D and 2D NMR of compounds **1**–**11** are included in the Supplementary data. CCDC 1522759 contains the supplementary crystallographic data for this paper. These data can be obtained free of charge via http://www.ccdc.cam.ac.uk/conts/retrieving.html (or from the CCDC, 12 Union Road, Cambridge CB2 1EZ, UK; Fax: +44-1223-336-033; E-mail: deposit@ccdc.cam.ac.uk).
