# Supplementary material for: Isolation, Characterization and Antiproliferative Activity of New Metabolites from the South African Endemic Red Algal Species Laurencia alfredensis
Source: Molecules. 2017 Mar 23;22(4):513. doi: 10.3390/molecules22040513 (PMC6154597; doi:10.3390/molecules22040513)
Supplement: Supplementary file 1 [file molecules-22-00513-s001.pdf]

Supplementary Materials: Isolation, characterization and antiproliferative activity of new metabolites from the South African endemic red algal species *Laurencia alfredensis*

Godwin A. Dziwornu, Mino R. Caira, Jo-Anne de la Mare, Adrienne L. Edkins, John J. Bolton, Denzil R. Beukes, Suthananda N. Sunassee

Table of Contents:

Scheme S1. Isolation of compounds **1 – 11** from *Laurencia alfredensis*

Figure S1. The structures of compounds **1 – 11** isolated from *Laurencia alfredensis*.

Figure S2. ^{1}H-NMR spectrum (600MHz, $CDCl_3$, 303K) of compound **1**

Figure S3. ^{13}C-NMR spectrum (150MHz, $CDCl_3$, 303K) of compound **1**

Figure S4. ^{1}H-^{1}H COSY NMR spectrum (600MHz, $CDCl_3$, 303K) of compound **1**

Figure S5. HSQC-DEPT NMR spectrum (600MHz, $CDCl_3$, 303K) of compound **1**

Figure S6. HMBC NMR spectrum (600MHz, $CDCl_3$, 303K) of compound **1**

Figure S7. ROESY NMR spectrum (600MHz, $CDCl_3$, 303K) of compound **1**

Figure S8. ^{1}H NMR spectrum (600MHz, $CDCl_3$, 303K) of compound **2**

Figure S9. ^{13}C NMR spectrum (150MHz, $CDCl_3$, 303K) of compound **2**

Figure S10. ^{1}H-^{1}H COSY NMR spectrum (600MHz, $CDCl_3$, 303K) of compound **2**

Figure S11. HSQC-DEPT NMR spectrum (600MHz, $CDCl_3$, 303K) of compound **2**

Figure S12. HMBC NMR spectrum (600MHz, $CDCl_3$, 303K) of compound **2**

Figure S13. ROESY NMR spectrum (600MHz, $CDCl_3$, 303K) of compound **2**

Figure S14. ^{1}H NMR spectrum (600MHz, $CDCl_3$, 303K) of compound **3**

Figure S15. ^{13}C NMR spectrum (150MHz, $CDCl_3$, 303K) of compound **3**

Figure S16. ^{1}H-^{1}H COSY NMR spectrum (600MHz, $CDCl_3$, 303K) of compound **3**

Figure S17. HSQC-DEPT NMR spectrum (600MHz, $CDCl_3$, 303K) of compound **3**

Figure S18. HMBC NMR spectrum (600MHz, $CDCl_3$, 303K) of compound **3**

Figure S19. ^{1}H NMR spectrum (600MHz, $CDCl_3$, 303K) of compound **4**

Figure S20. ^{13}C NMR spectrum (150MHz, $CDCl_3$, 303K) of compound **4**

Figure S21. ^{1}H-^{1}H COSY NMR spectrum (600MHz, $CDCl_3$, 303K) of compound **4**

Figure S22. HSQC-DEPT NMR spectrum (600MHz, $CDCl_3$, 303K) of compound **4**

Figure S23. HMBC NMR spectrum (600MHz, $CDCl_3$, 303K) of compound **4**

Figure S24. ROESY NMR spectrum (600MHz, $CDCl_3$, 303K) of compound **4**

Figure S25. ^{1}H NMR spectrum (600MHz, $CDCl_3$, 303K) of compounds **5** and **6**

Figure S26. ^{13}C NMR spectrum (150MHz, $CDCl_3$, 303K) of compounds **5** and **6**

Figure S27. ^{1}H-^{1}H COSY NMR spectrum (600MHz, $CDCl_3$, 303K) of compounds **5** and **6**

Figure S28. HSQC-DEPT NMR spectrum (600MHz, $CDCl_3$, 303K) of compounds **5** and **6**

Figure S29. HMBC NMR spectrum (600MHz, $CDCl_3$, 303K) of compounds **5** and **6**

Figure S30. ROESY NMR spectrum (600MHz, $CDCl_3$, 303K) of compounds **5** and **6**

Figure S31. ^{1}H NMR spectrum (600MHz, $CDCl_3$, 303K) of compound **7**

Figure S32. ^{13}C NMR spectrum (150MHz, $CDCl_3$, 303K) of compound **7**

Figure S33. ^{1}H-^{1}H COSY NMR spectrum (600MHz, $CDCl_3$, 303K) of compound **7**

Figure S34. HSQC-DEPT NMR spectrum (600MHz, $CDCl_3$, 303K) of compound **7**

Figure S35. HMBC NMR spectrum (600MHz, $CDCl_3$, 303K) of compound **7**

Figure S36. ROESY NMR spectrum (600MHz, $CDCl_3$, 303K) of compound **7**

Figure S37. ^{1}H NMR spectrum (600MHz, $CDCl_3$, 303K) of compound **8**

Figure S38. ^{13}C NMR spectrum (150MHz, $CDCl_3$, 303K) of compound **8**

Figure S39. ^{1}H-^{1}H COSY NMR spectrum (600MHz, $CDCl_3$, 303K) of compound **8**

Figure S40. HSQC-DEPT NMR spectrum (600MHz, $CDCl_3$, 303K) of compound **8**

Figure S41. HMBC NMR spectrum (600MHz, $CDCl_3$, 303K) of compound **8**

Figure S42. NOESY NMR spectrum (600MHz, $CDCl_3$, 303K) of compound **8**

Figure S43. ^{1}H-NMR spectrum (600MHz, $CDCl_3$, 303K) of compound **9**

Figure S44. ^{13}C-NMR spectrum (150MHz, $CDCl_3$, 303K) of compound **9**

Figure S45. ^{1}H-^{1}H COSY NMR spectrum (600MHz, $CDCl_3$, 303K) of compound **9**

Figure S46. HSQC-DEPT NMR spectrum (600MHz, $CDCl_3$, 303K) of compound **9**

Figure S47. HMBC NMR spectrum (600MHz, $CDCl_3$, 303K) of compound **9**

Figure S48. ROESY NMR spectrum (600MHz, $CDCl_3$, 303K) of compound **9**

Figure S49. ^{1}H-NMR spectrum (600MHz, $CDCl_3$, 303K) of compound **10**

Figure S50. ^{13}C-NMR spectrum (150MHz, $CDCl_3$, 303K) of compound **10**

Figure S51. ^1H-^1H COSY NMR spectrum (600MHz, $CDCl_3$, 303K) of compound **10**

Figure S52. HSQC-DEPT NMR spectrum (600MHz, $CDCl_3$, 303K) of compound **10**

Figure S53. HMBC NMR spectrum (600MHz, $CDCl_3$, 303K) of compound **10**

Figure S54. ROESY NMR spectrum (600MHz, $CDCl_3$, 303K) of compound **10**

Figure S55. ^1H-NMR spectrum (600MHz, DMSO-d_6, 303K) of compound **11**

Figure S56. ^{13}C-NMR spectrum (150MHz, DMSO-d_6, 303K) of compound **11**

Figure S57. ^1H-^1H COSY NMR spectrum (600MHz, DMSO-d_6, 303K) of compound **11**

Figure S58. HSQC-DEPT NMR spectrum (600MHz, DMSO-d_6, 303K) of compound **11**

Figure S59. HMBC NMR spectrum (600MHz, DMSO-d_6, 303K) of compound **11**

Figure S60. HR-ESI-MS spectrum of compound **1**

Figure S61. HR-ESI-MS spectrum of compound **2**

Figure S62. HR-ESI-MS spectrum of compound **3**

Figure S63. HR-ESI-MS spectrum of compound **5**

Figure S64. HR-ESI-MS spectrum of compound **6**

Figure S65. HR-ESI-MS spectrum of compound **7**

Figure S66. HR-ESI-MS spectrum of compound **8**

Figure S67. HR-ESI-MS spectrum of compound **9**

Figure S68. HR-ESI-MS spectrum of compound **10**

Figure S69. HR-ESI-MS spectrum of compound **11**

Figure S70. IR spectrum of compound **1**

Figure S71. IR spectrum of compound **2**

Figure S72. IR spectrum of compound **3**

Figure S73. IR spectrum of compound **7**

Figure S74. IR spectrum of compound **8**

Figure S75. IR spectrum of compound **10**

Figure S76. UV spectrum of compound **8**

Figure S77. UV spectrum of compound **10**

Table S1. Cytotoxicity assay data for compounds **1 - 10**

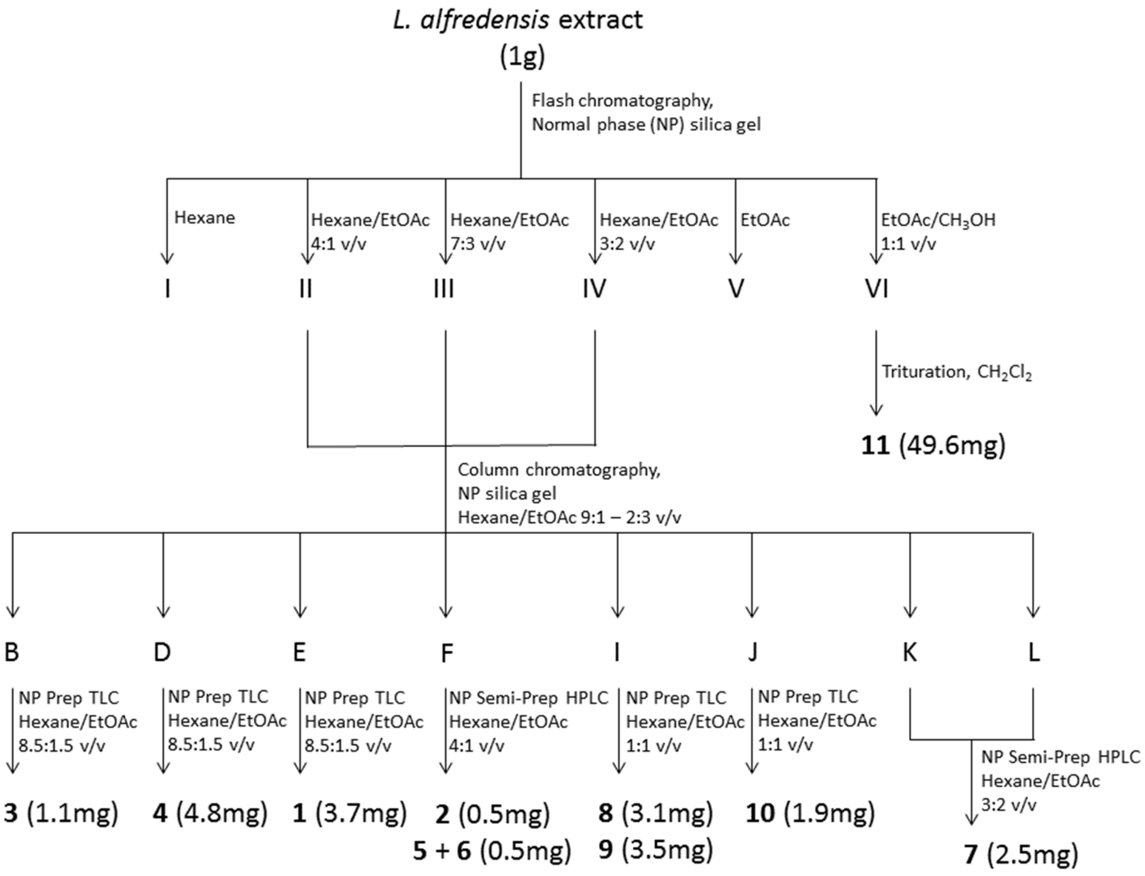

Scheme S1. Isolation of compounds **1** – **11** from *Laurencia alfredensis*

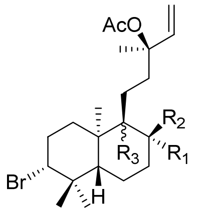

1 R_1 = OH, R_2 = Me, R_3 = H
2 R_1 = Me, R_2 = OH, R_3 = H
3 R_1 = H, R_2 = Me, R_3 = OH

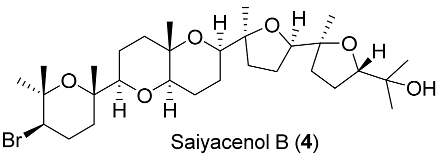

Saiyacenol B (**4**)

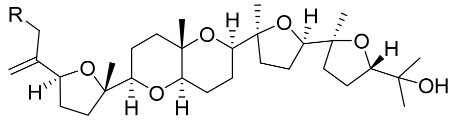

5 R = H
6 R = OH

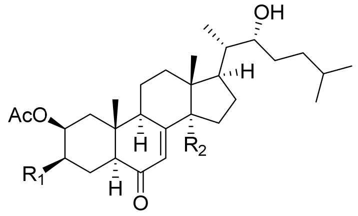

8 R_1 = OAc, R_2 = H
9 R_1 = OAc, R_2 = OH
10 R_1 = OH, R_2 = H

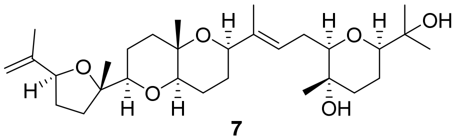

7

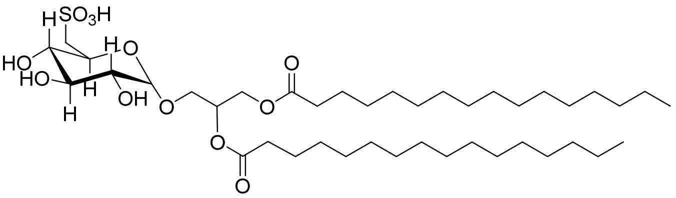

1, 2-di-*O*-palmitoyl-3-*O*-(6-sulfo-a-D-quinovopyranosyl)-glycerol
(**11**)

Figure S1. The structures of compounds **1 – 11** isolated from *Laurencia alfredensis*.

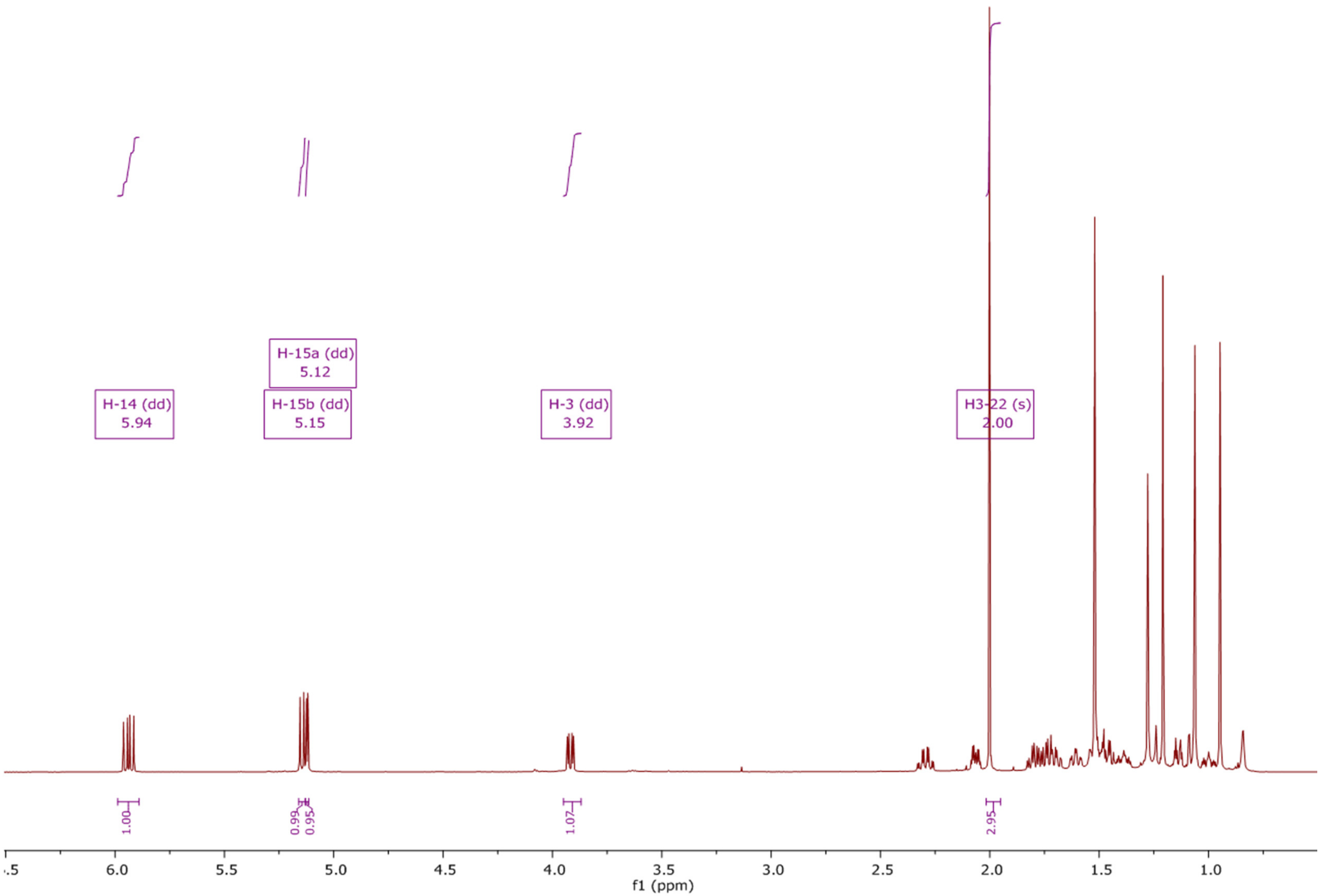

Figure S2. ^{1}H-NMR spectrum (600MHz, $CDCl_3$, 303K) of compound **1**

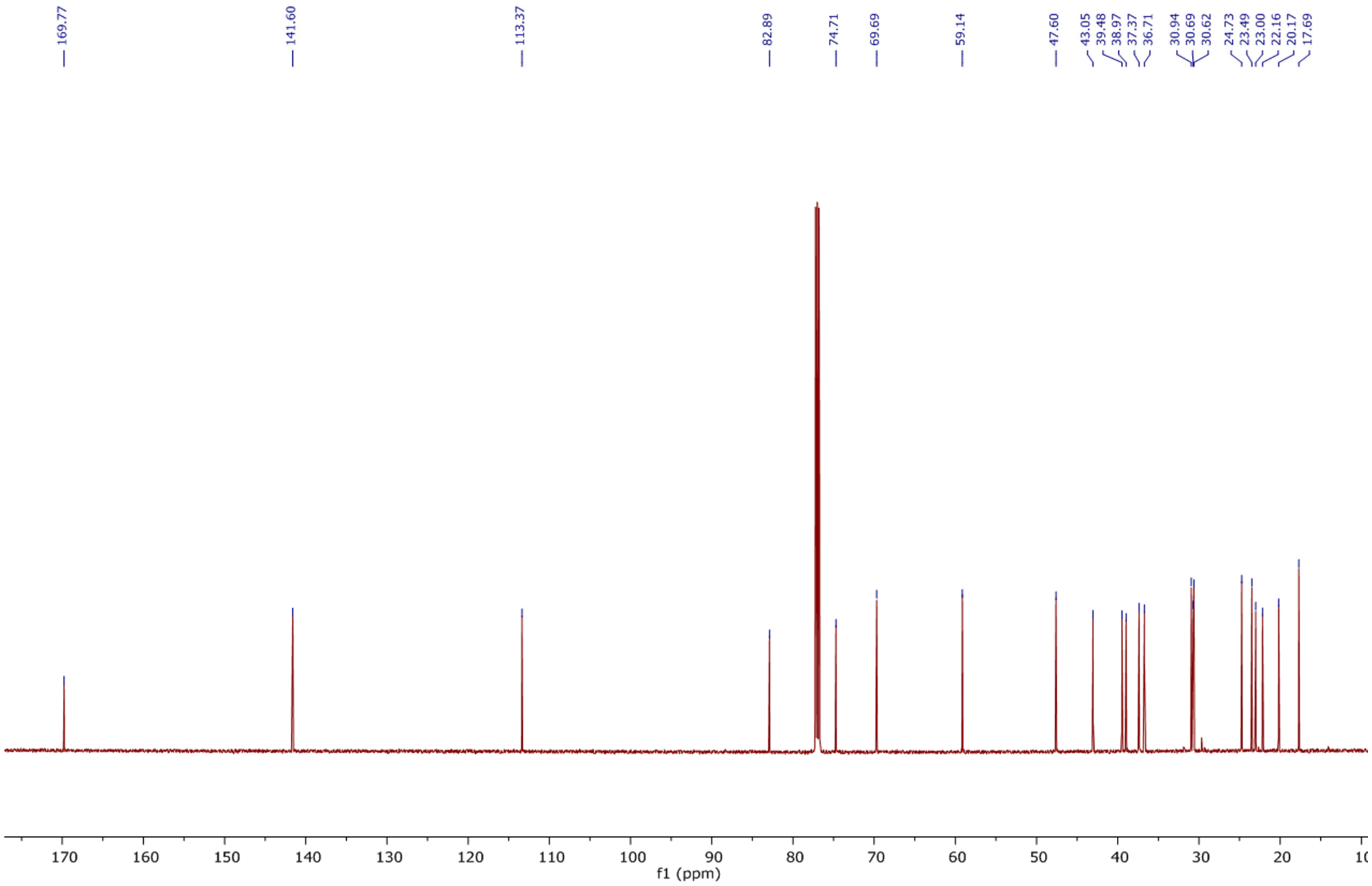

Figure S3. ^{13}C-NMR spectrum (150MHz, $CDCl_3$, 303K) of compound **1**

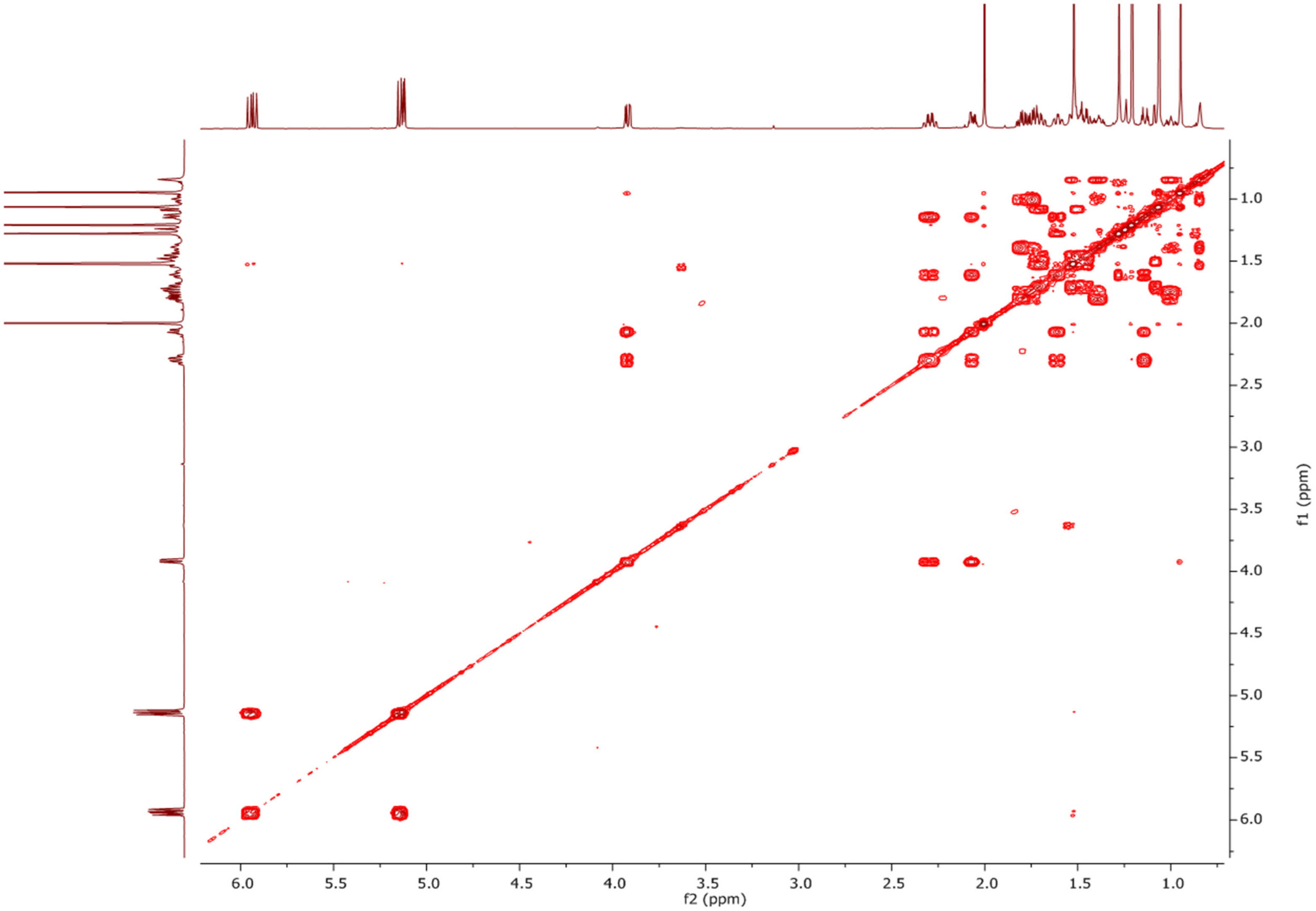

Figure S4. ¹H-¹H COSY spectrum (600MHz, $CDCl_3$, 303K) of compound **1**

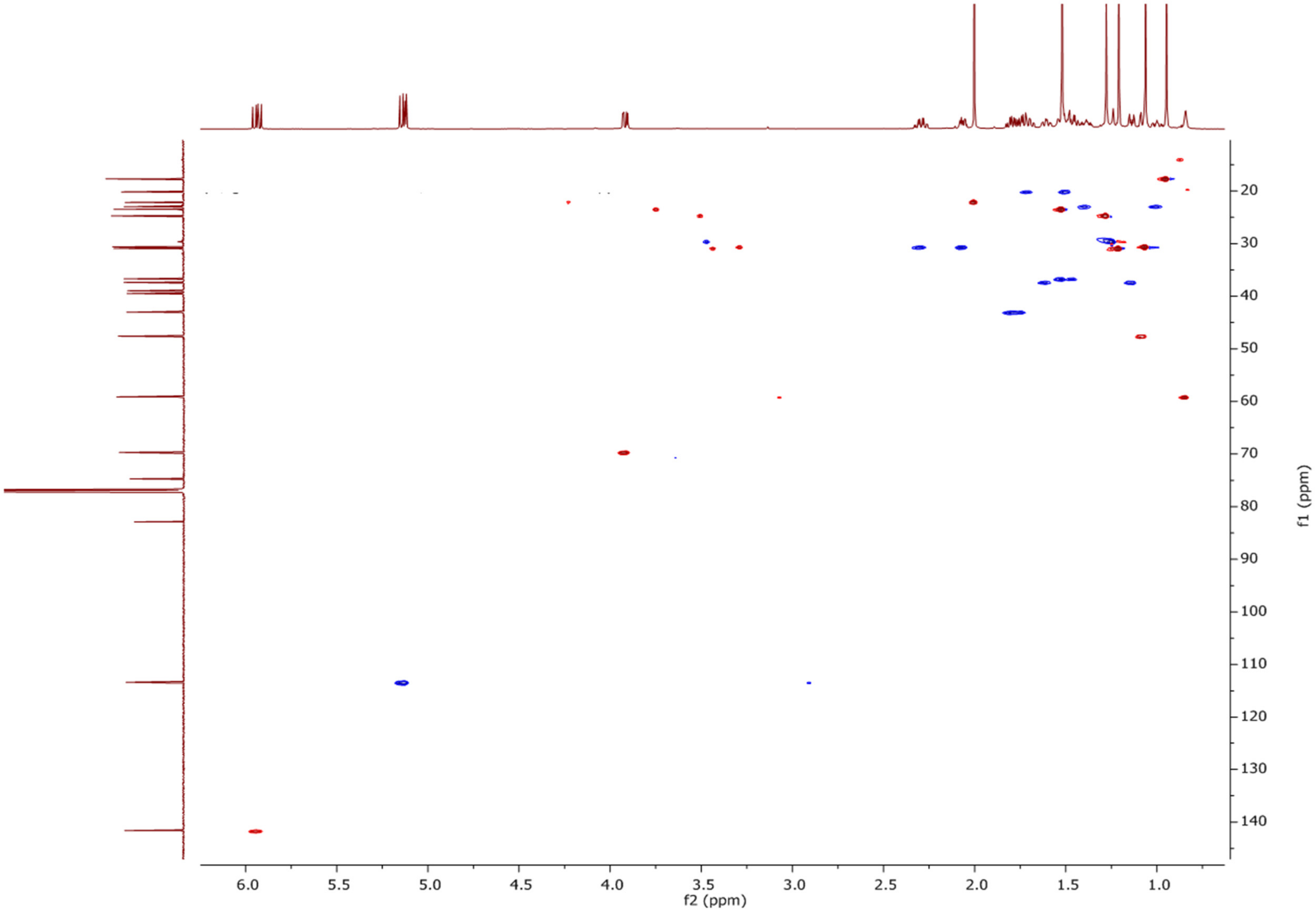

Figure S5. HSQC-DEPT NMR spectrum (600MHz, $CDCl_3$, 303K) of compound **1**

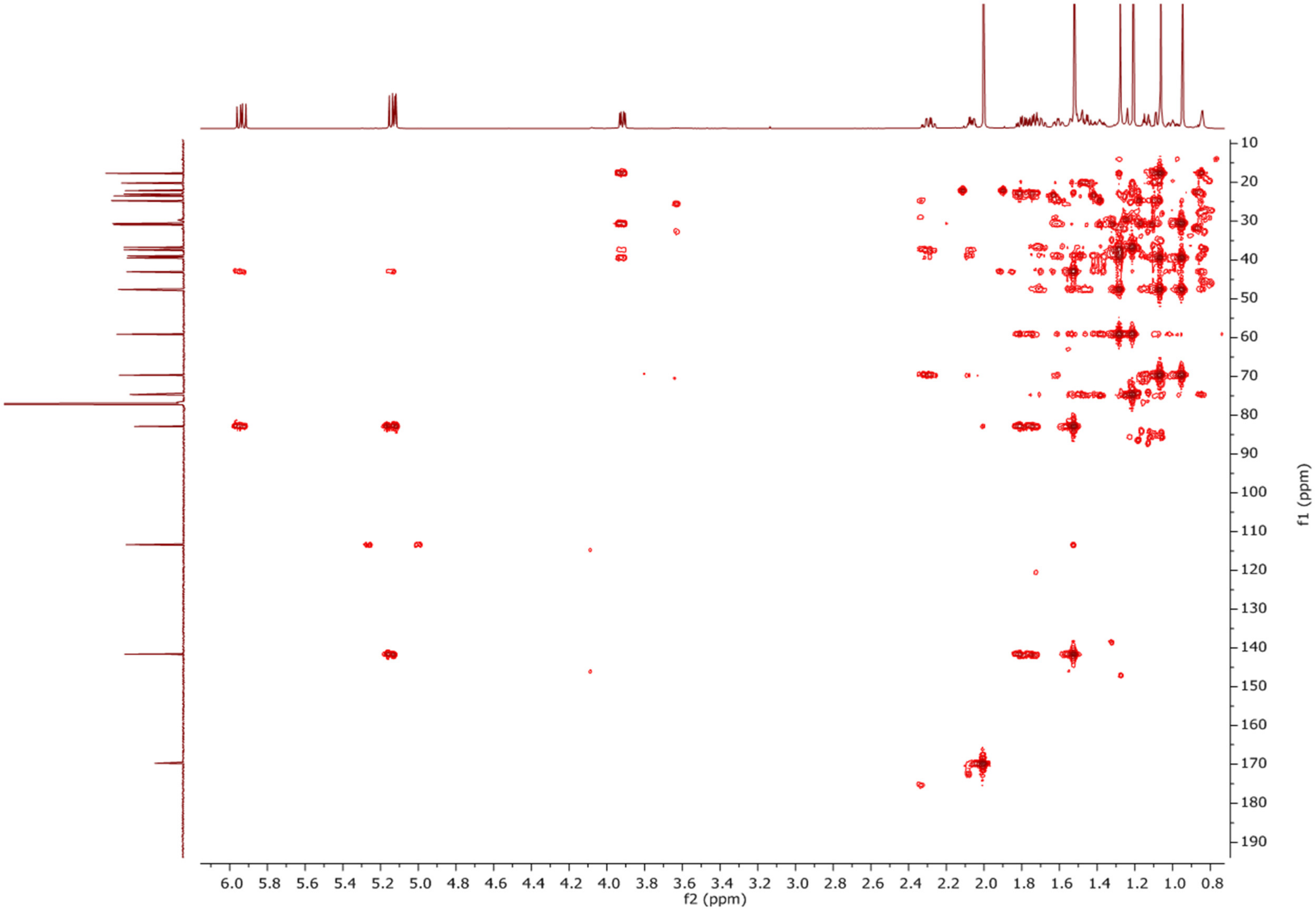

Figure S6. HMBC NMR spectrum (600MHz, $CDCl_3$, 303K) of compound **1**

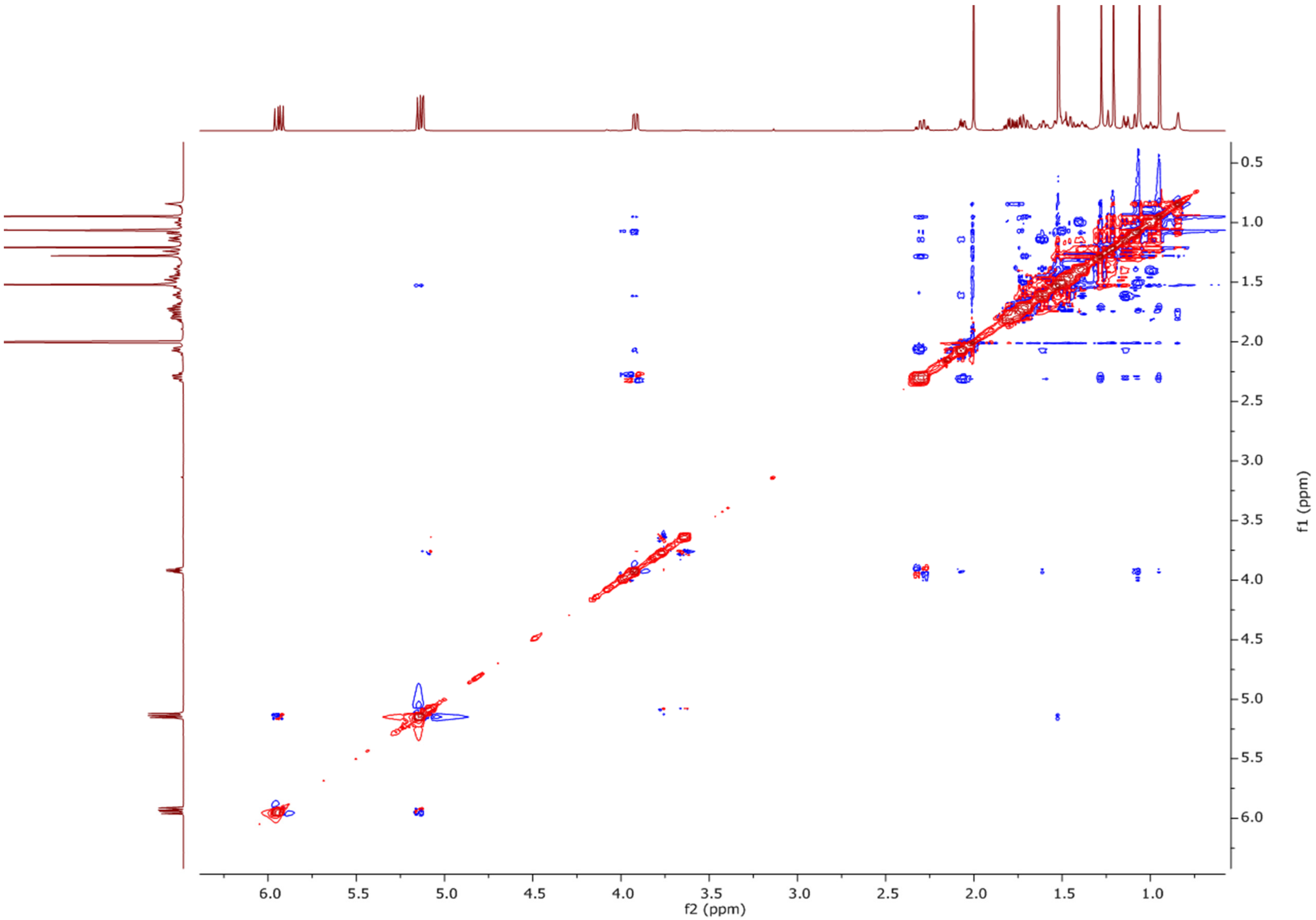

Figure S7. ROESY NMR spectrum (600MHz, $CDCl_3$, 303K) of compound **1**

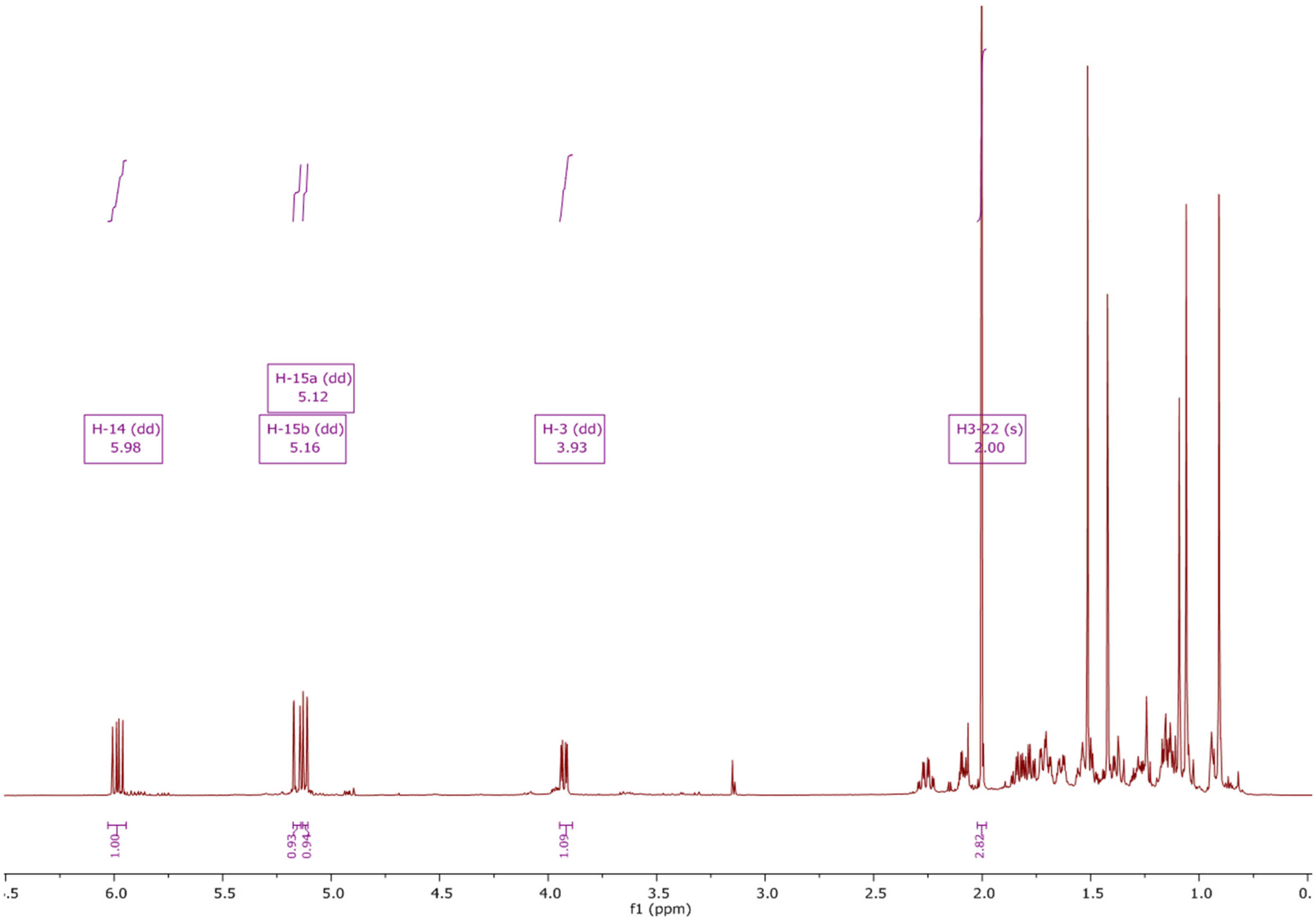

Figure S8. ¹H-NMR spectrum (600MHz, $CDCl_3$, 303K) of compound **2**

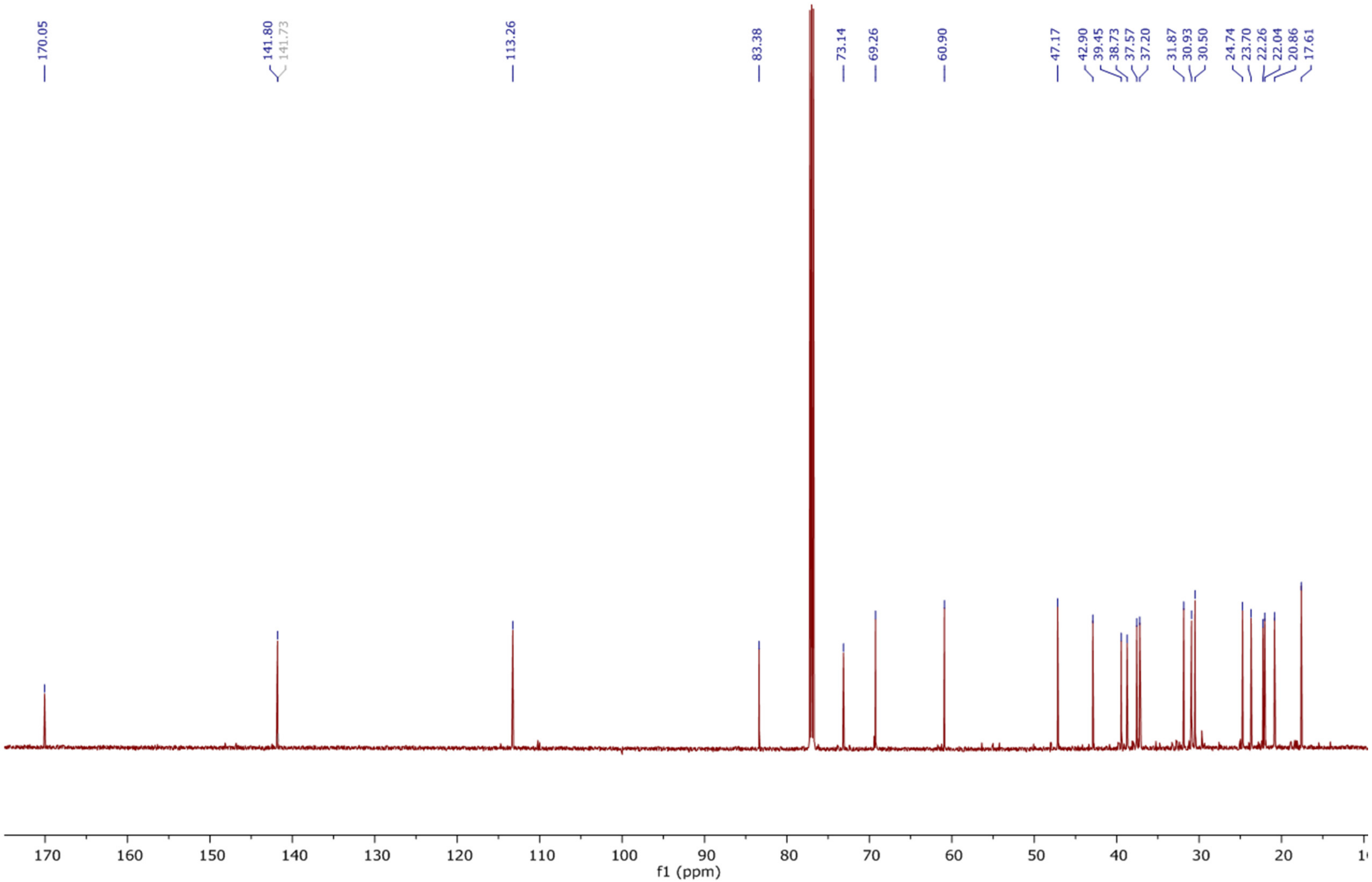

Figure S9. ^{13}C-NMR spectrum (150MHz, $CDCl_3$, 303K) of compound **2**

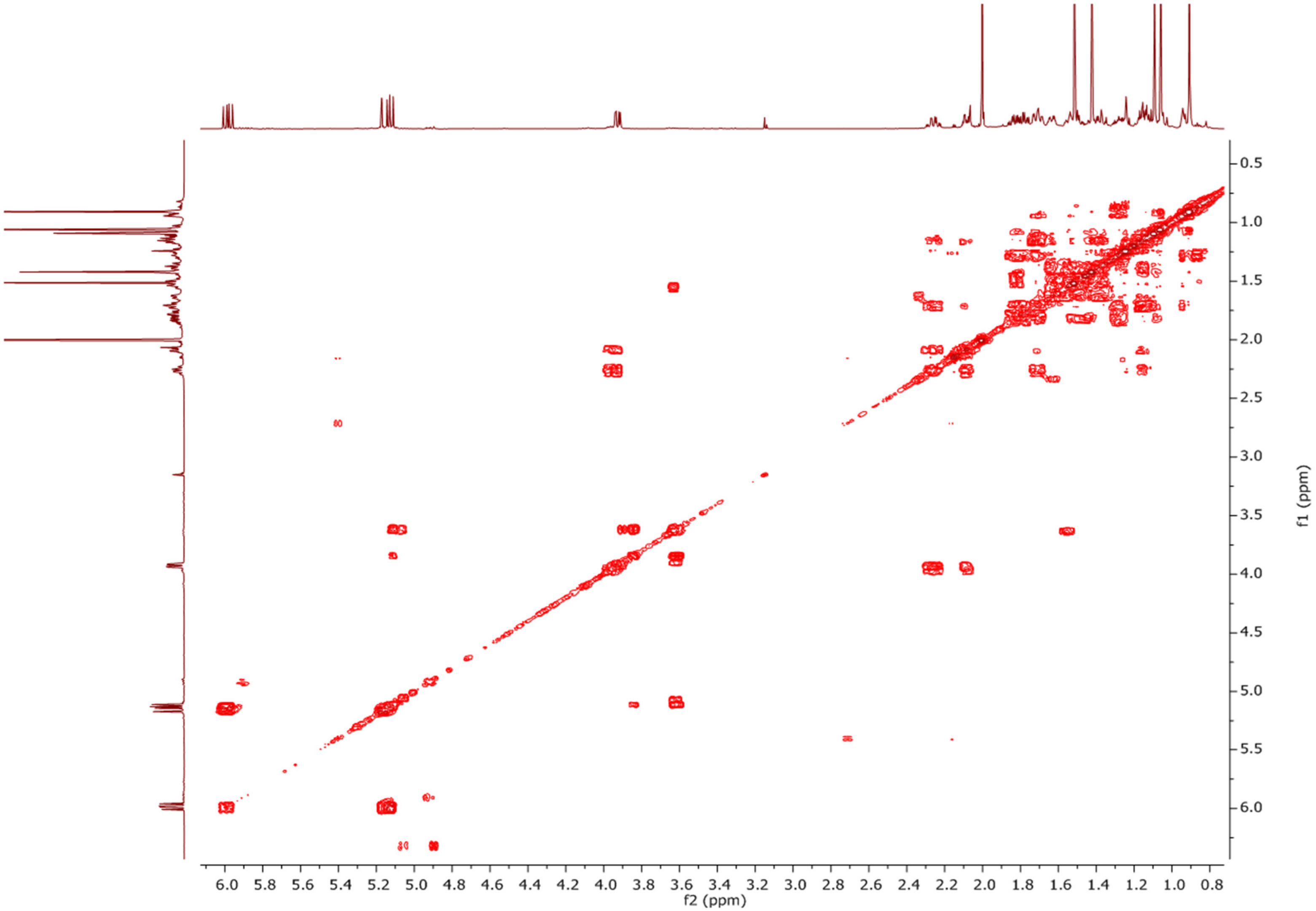

Figure S10. ^{1}H-^{1}H COSY NMR spectrum (600MHz, $CDCl_3$), 303K of compound **2**

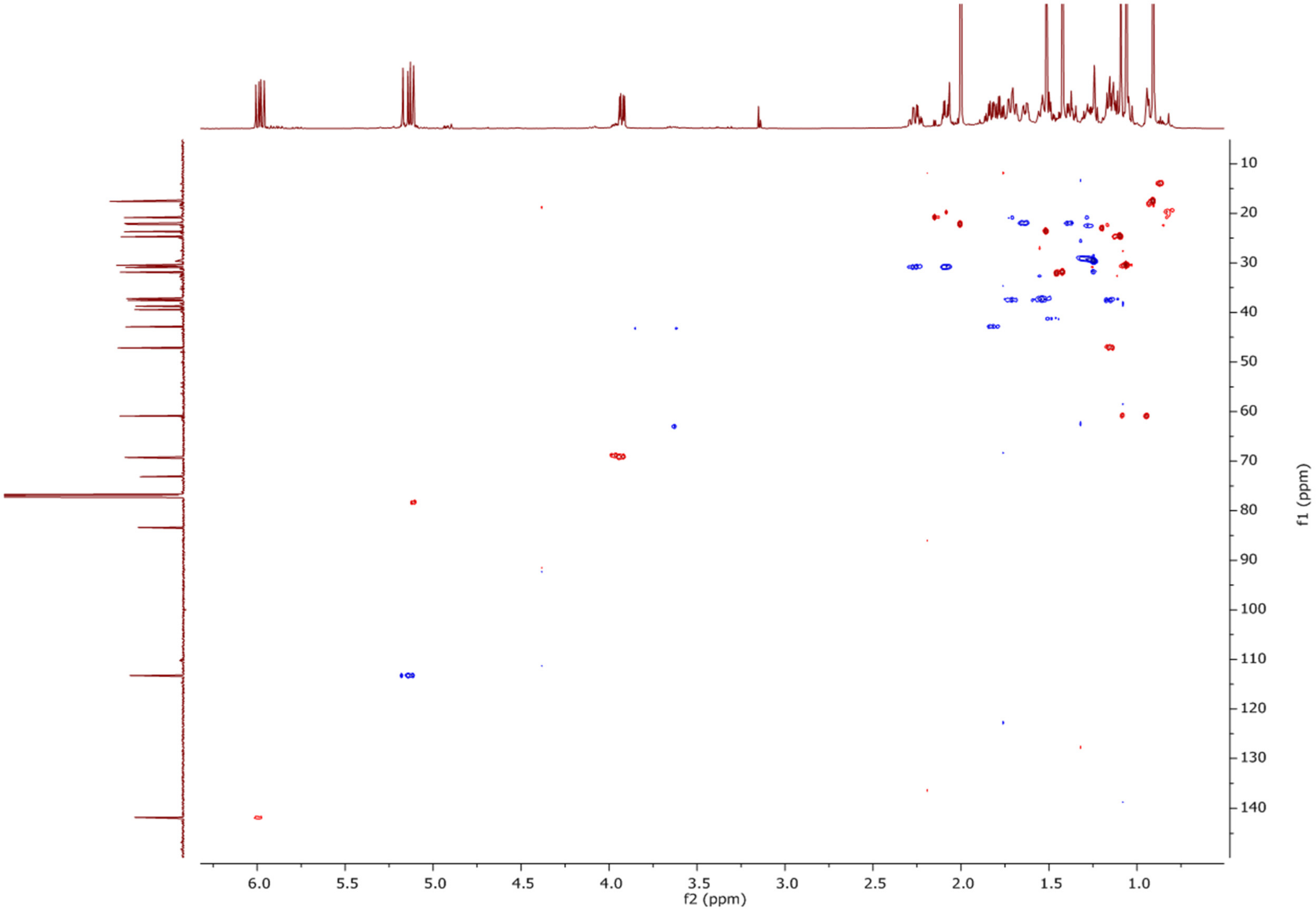

Figure S11. HSQC-DEPT NMR spectrum (600MHz, $CDCl_3$, 303K) of compound **2**

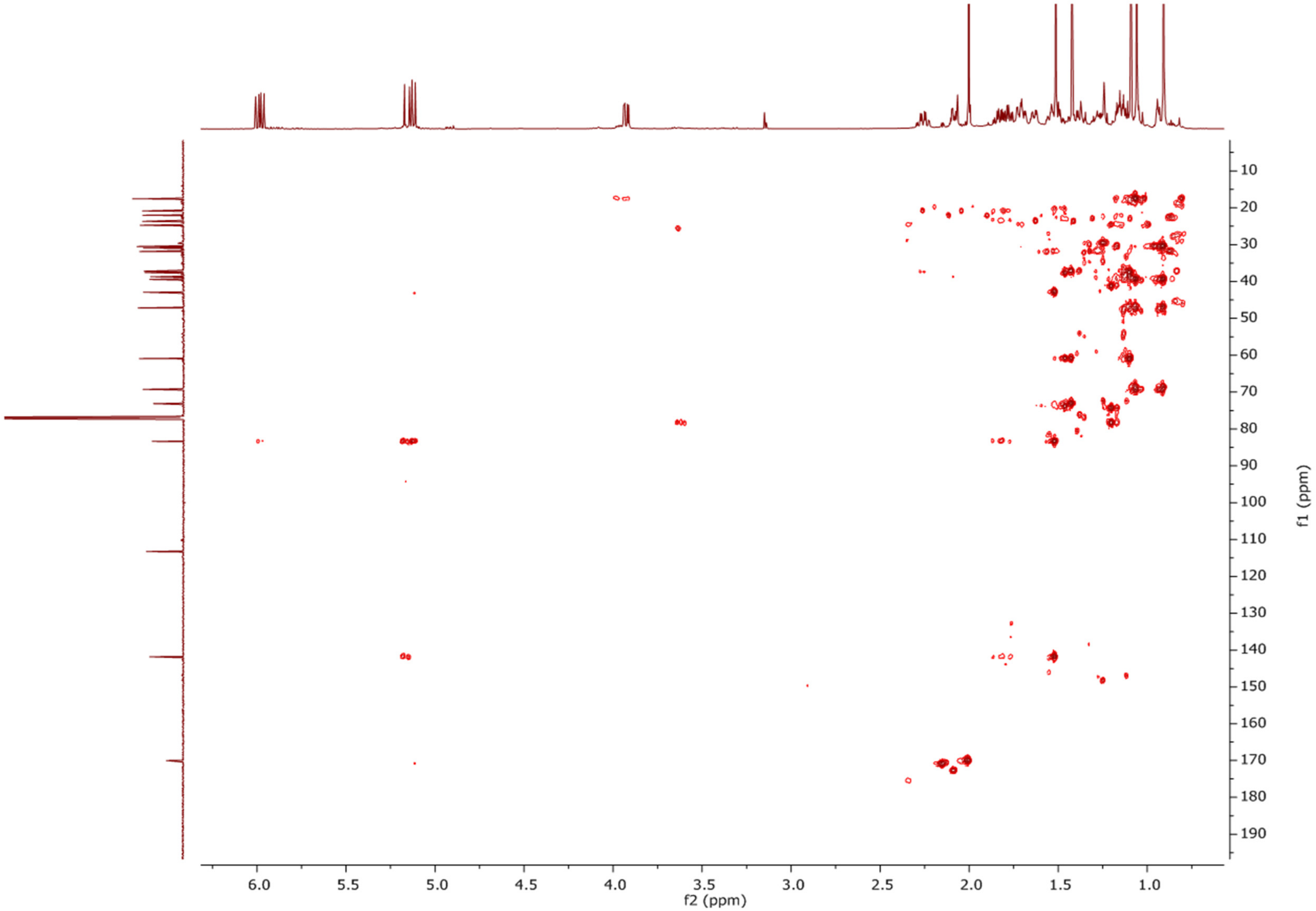

Figure S12. HMBC NMR spectrum (600MHz, $CDCl_3$, 303K) of compound **2**

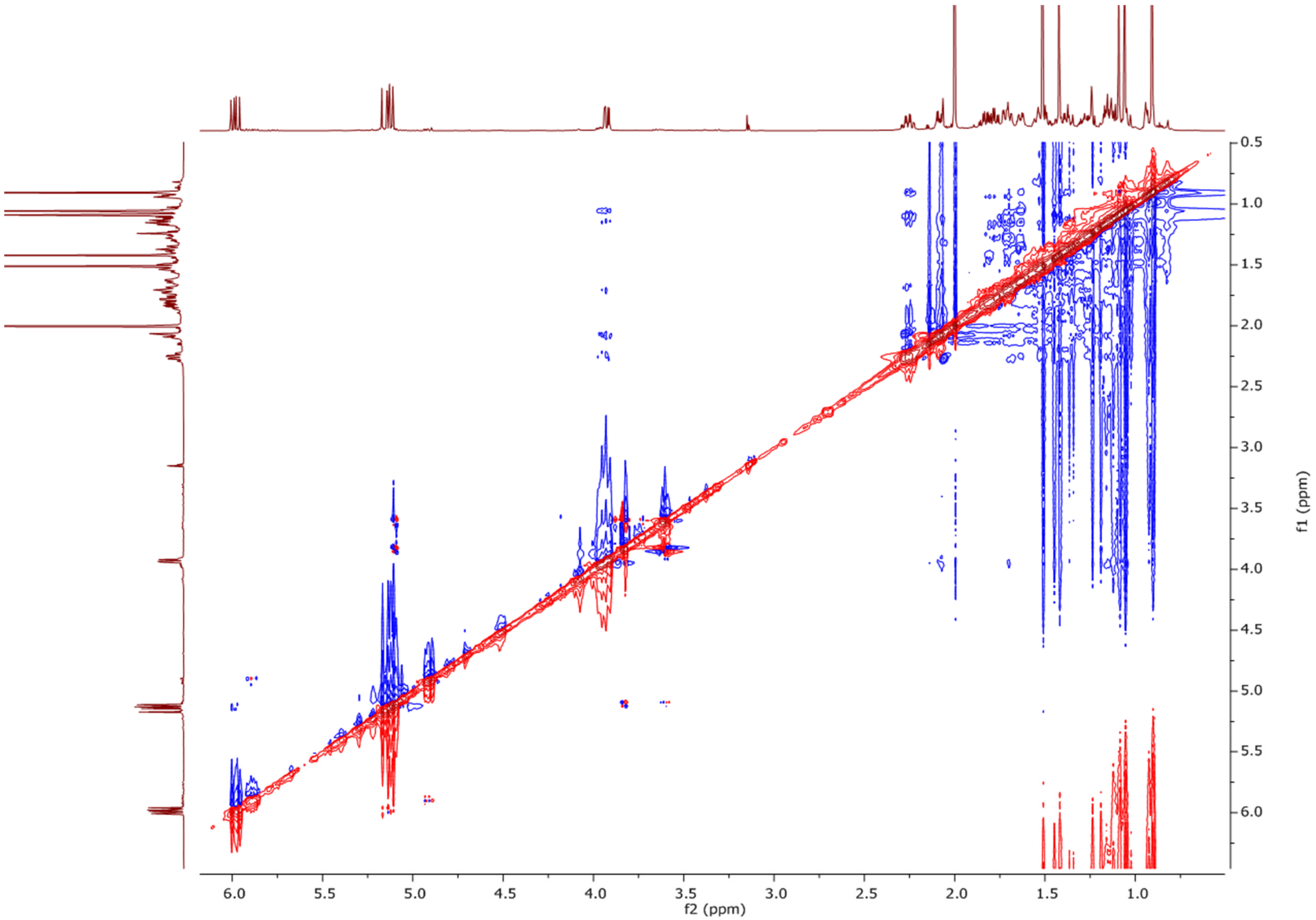

Figure S13. ROESY NMR spectrum (600MHz, $CDCl_3$, 303K) of compound **2**

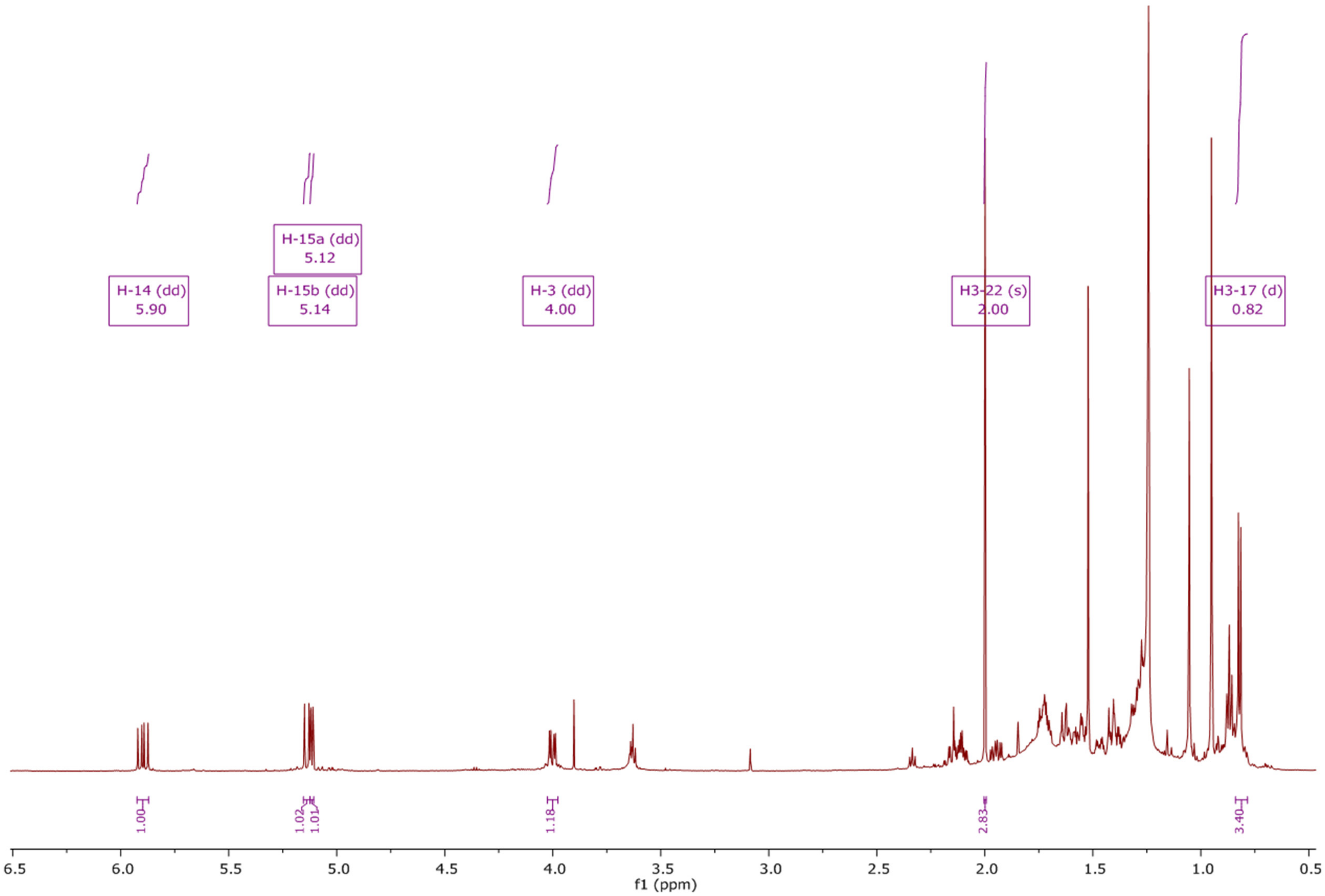

Figure S14. ^{1}H-NMR spectrum (600MHz, $CDCl_3$, 303K) of compound **3**

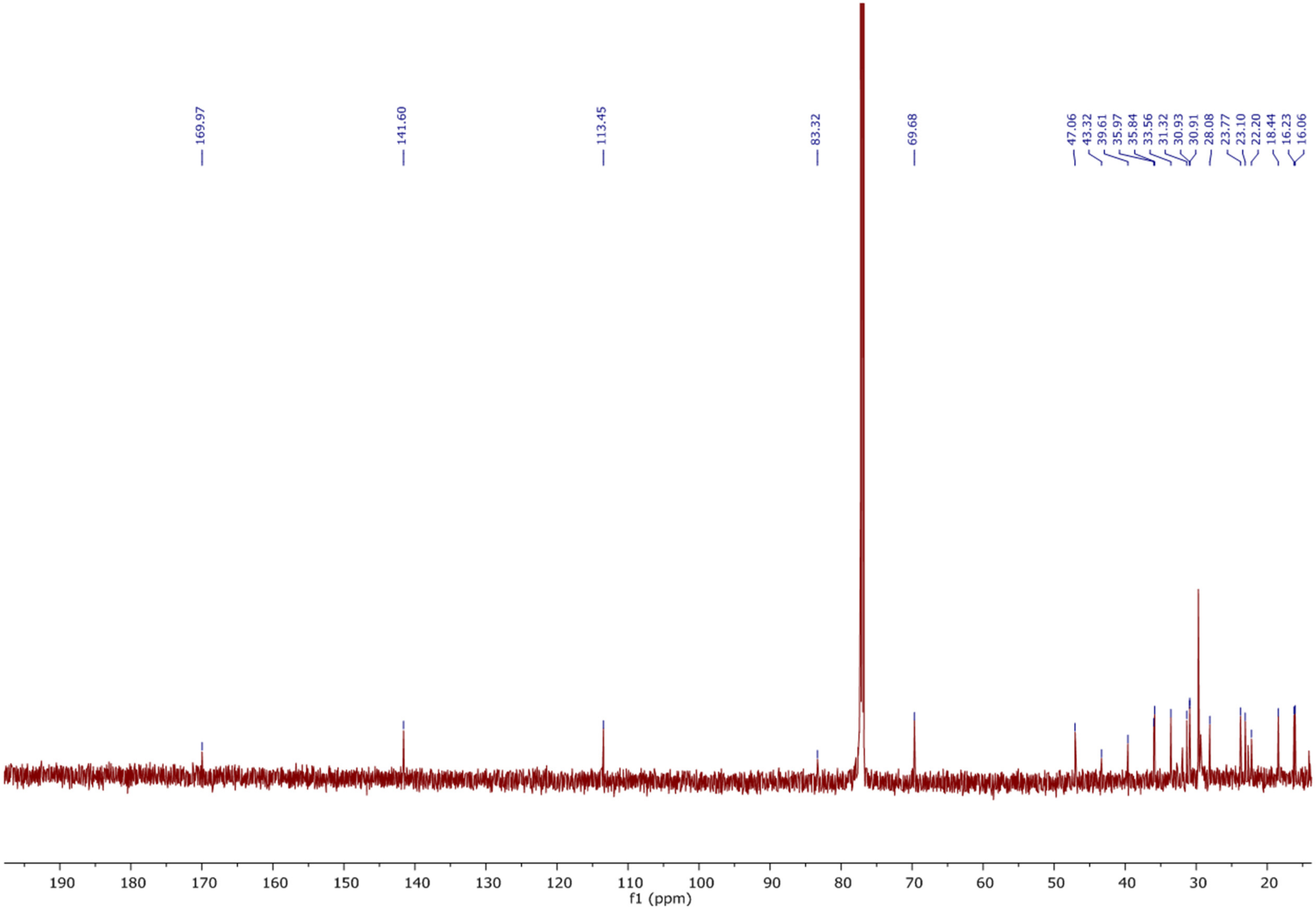

Figure S15. ^{13}C-NMR spectrum (150MHz, $CDCl_3$, 303K) of compound **3**

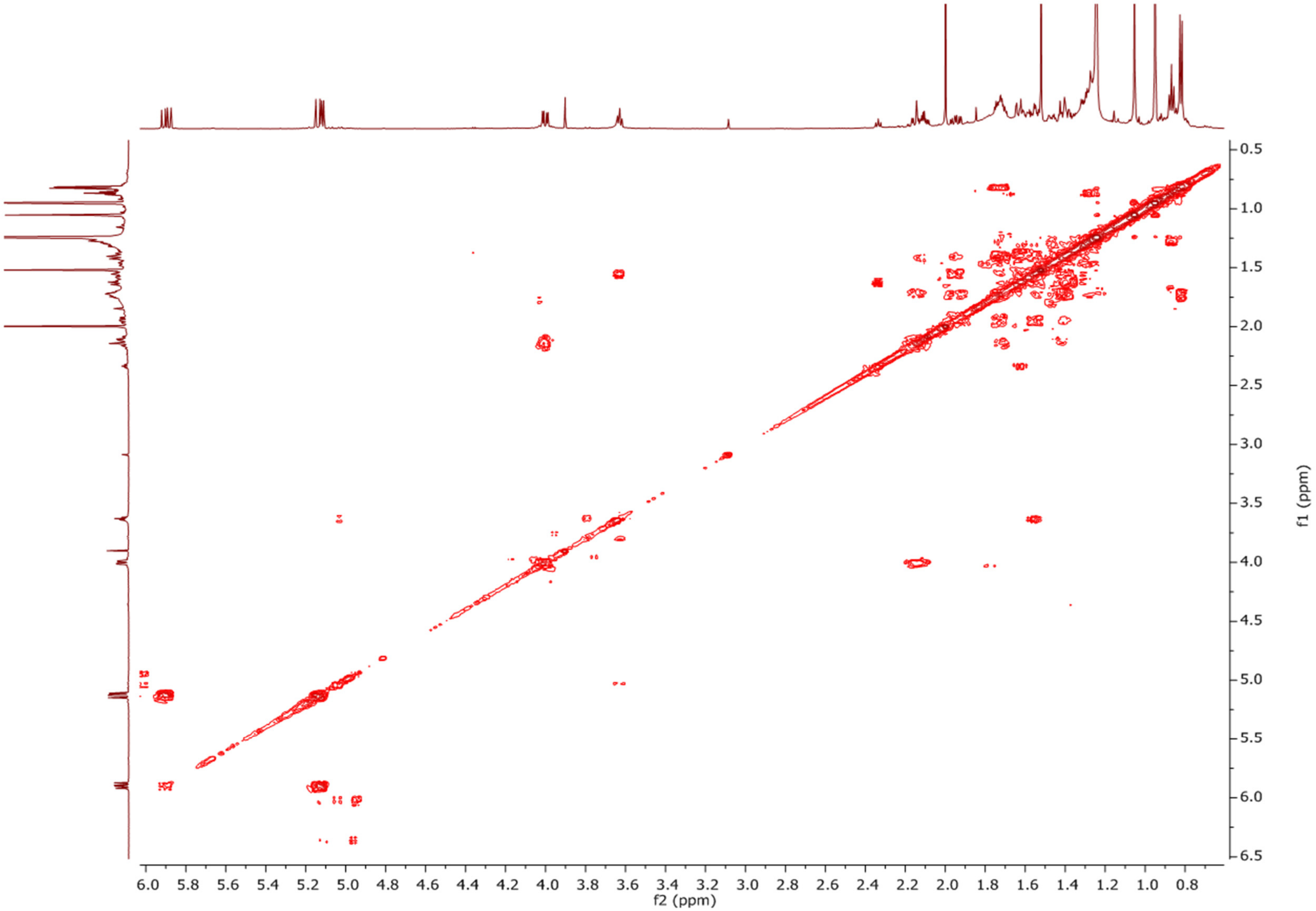

Figure S16. ^{1}H-^{1}H COSY NMR spectrum (600MHz, $CDCl_3$, 303K) of compound **3**

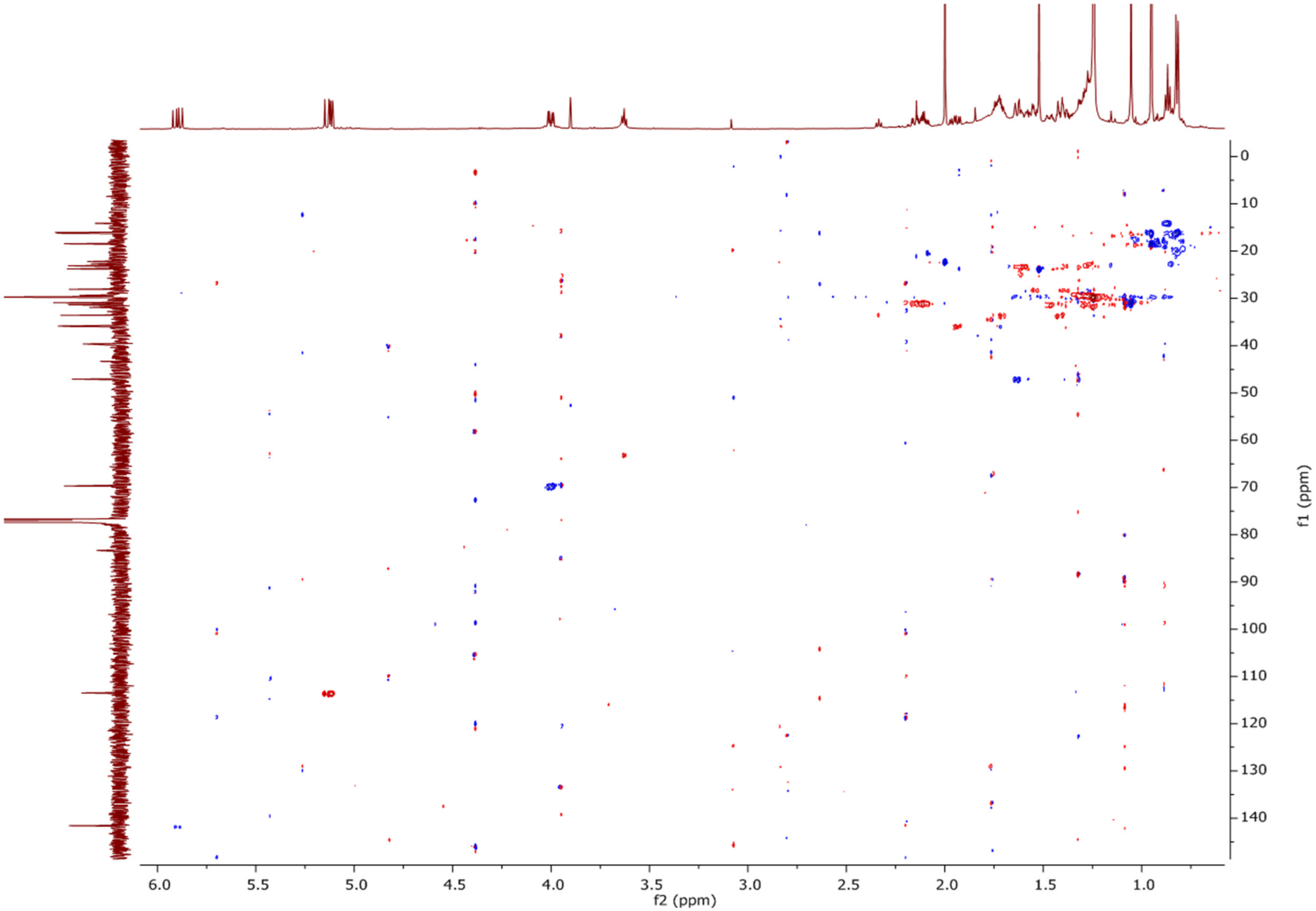

Figure S17. HSQC-DEPT NMR spectrum (600MHz, $CDCl_3$, 303K) of compound **3**

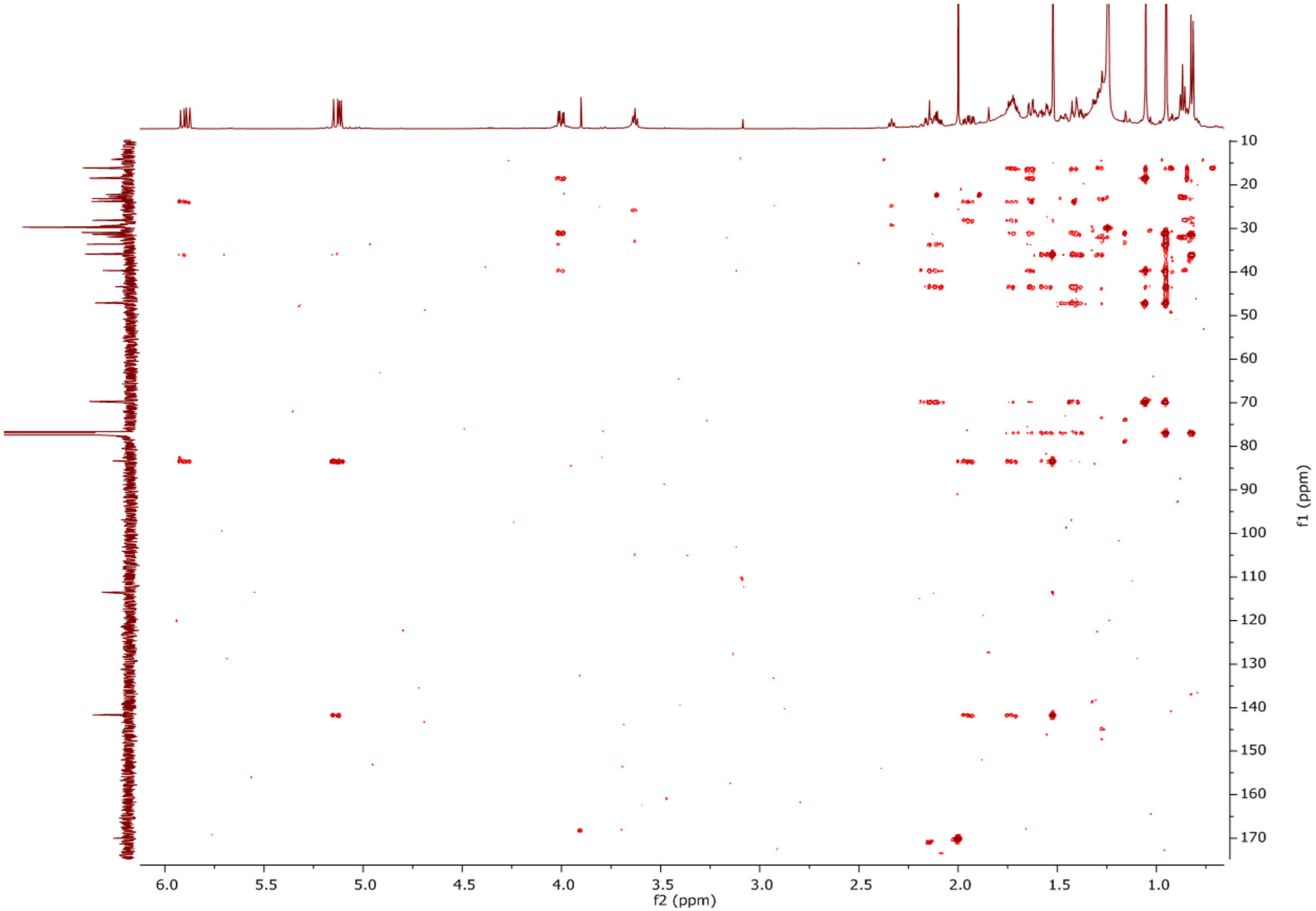

Figure S18. HMBC NMR spectrum (600MHz, $CDCl_3$, 303K) of compound **3**

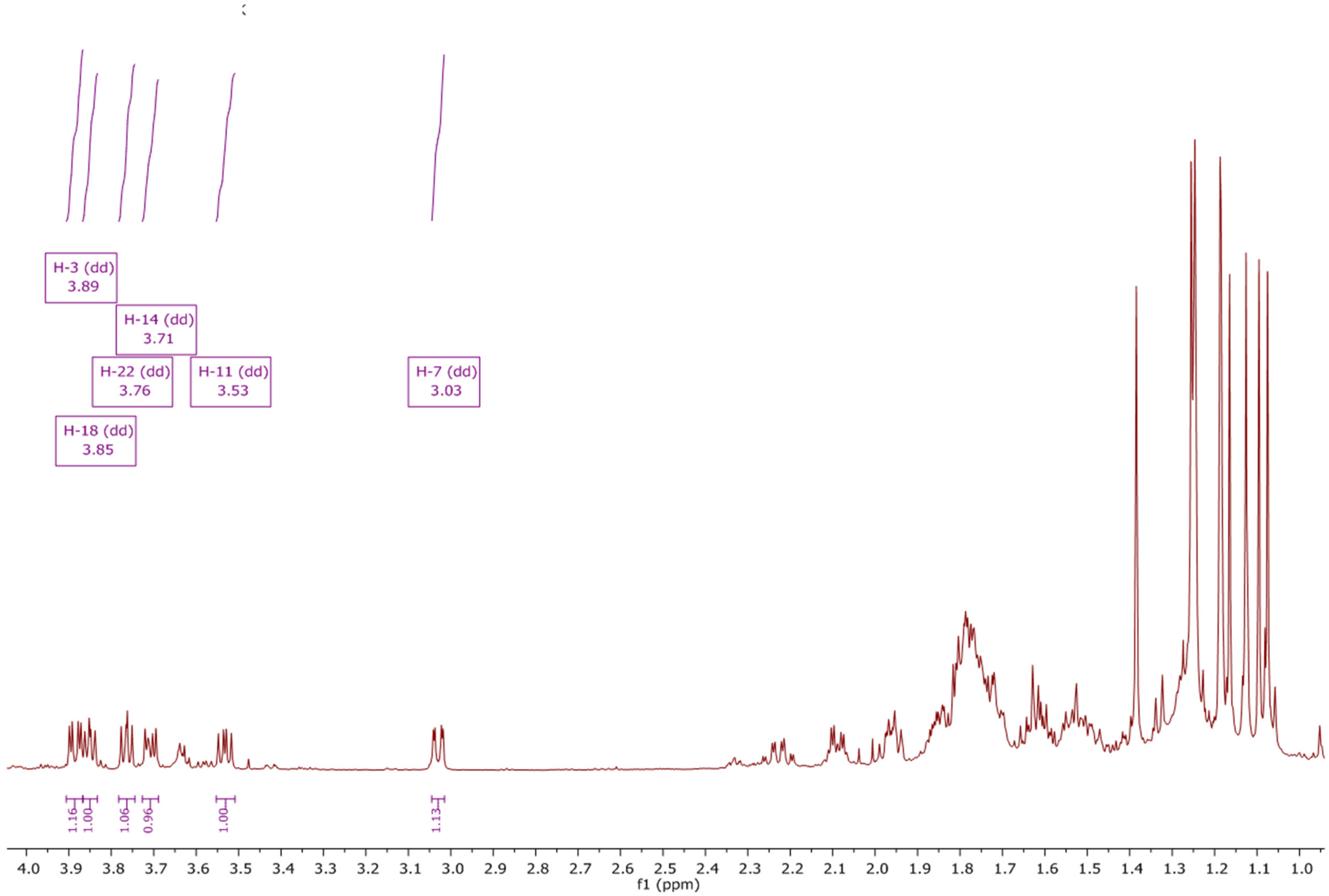

Figure S19. ^{1}H-NMR spectrum (600MHz, $CDCl_3$, 303K) of compound **4**

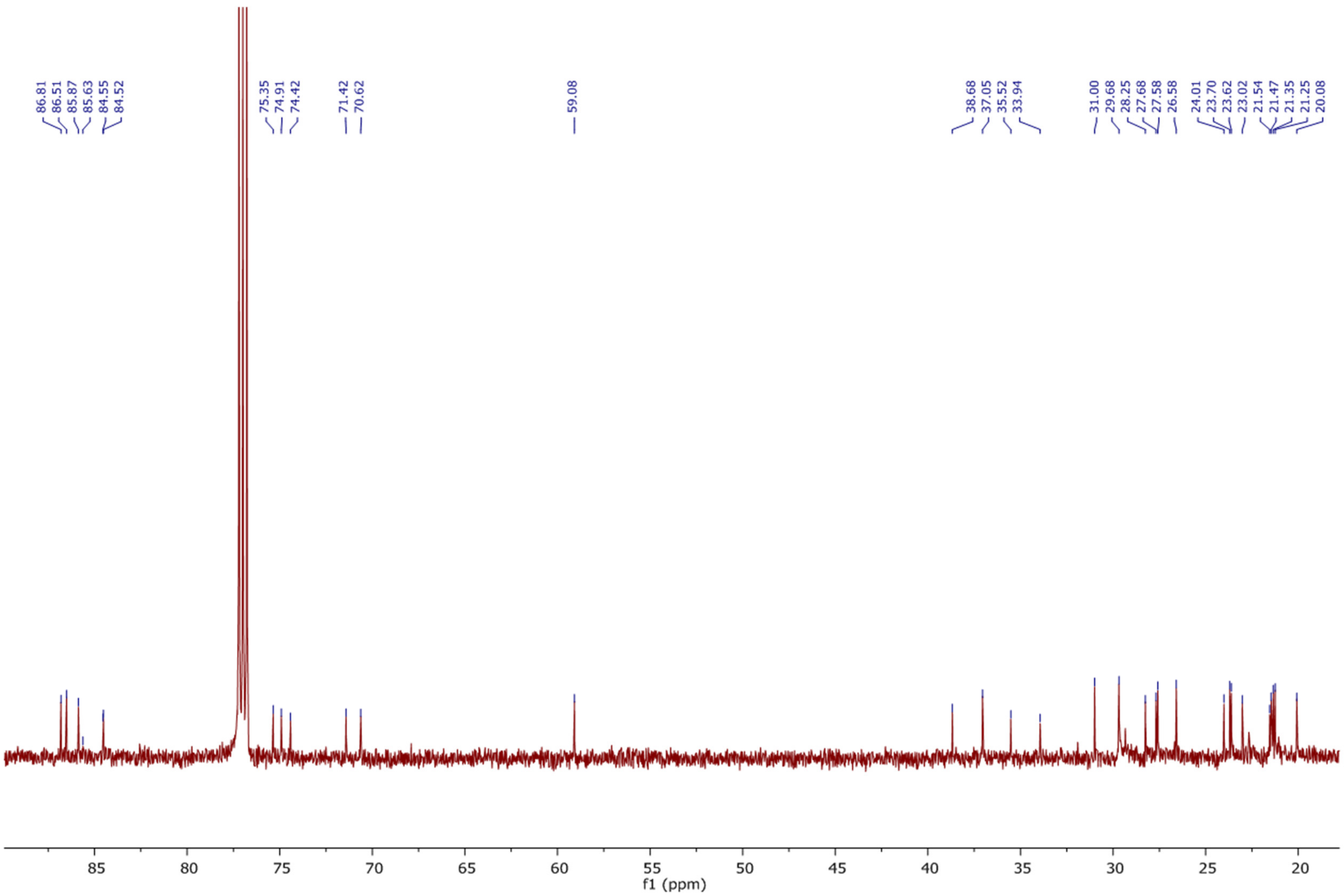

Figure S20. ^{13}C-NMR spectrum (150MHz, $CDCl_3$, 303K) of compound **4**

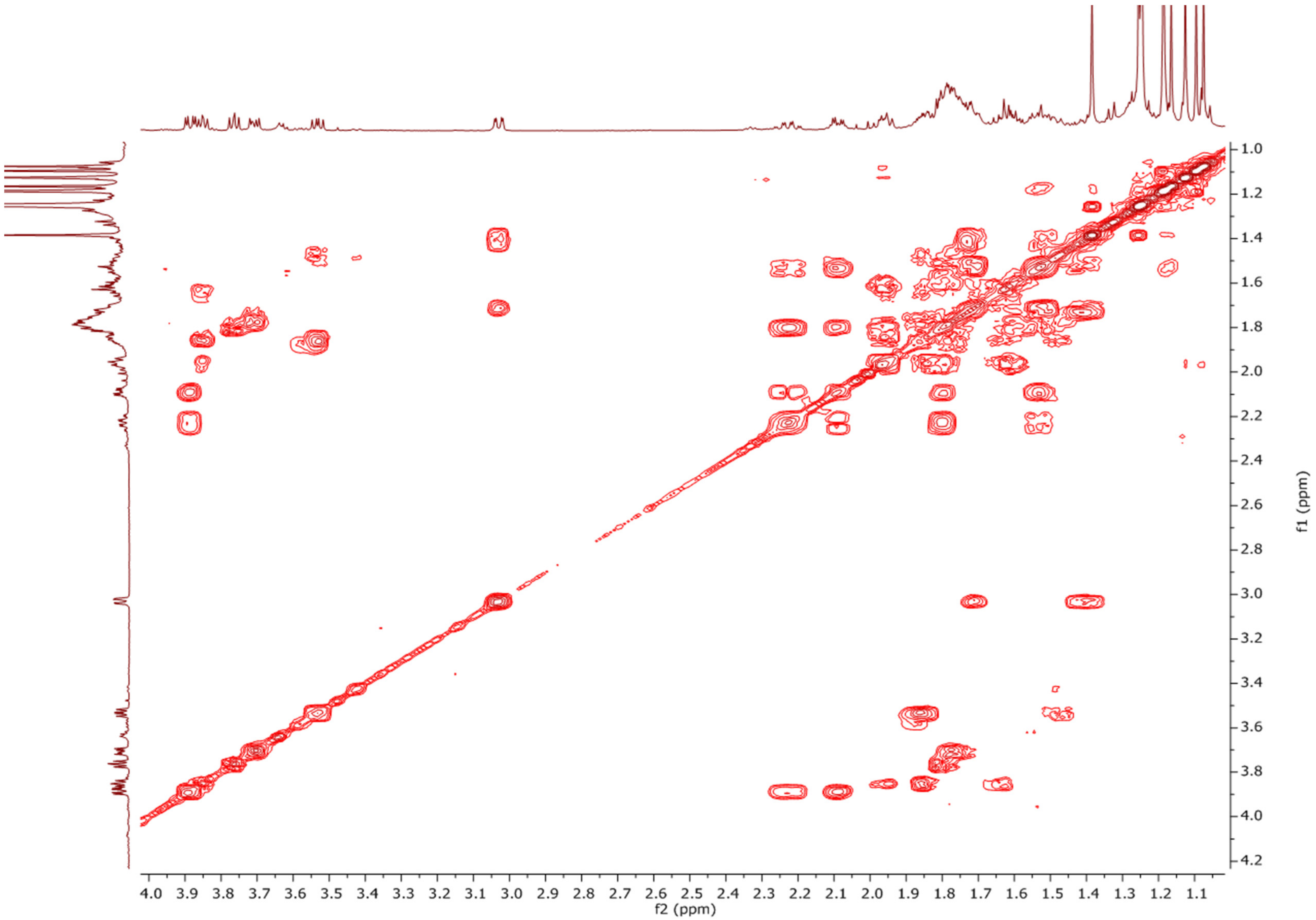

Figure S21. ^1H-^1H COSY NMR spectrum (600MHz, $CDCl_3$, 303K) of compound **4**

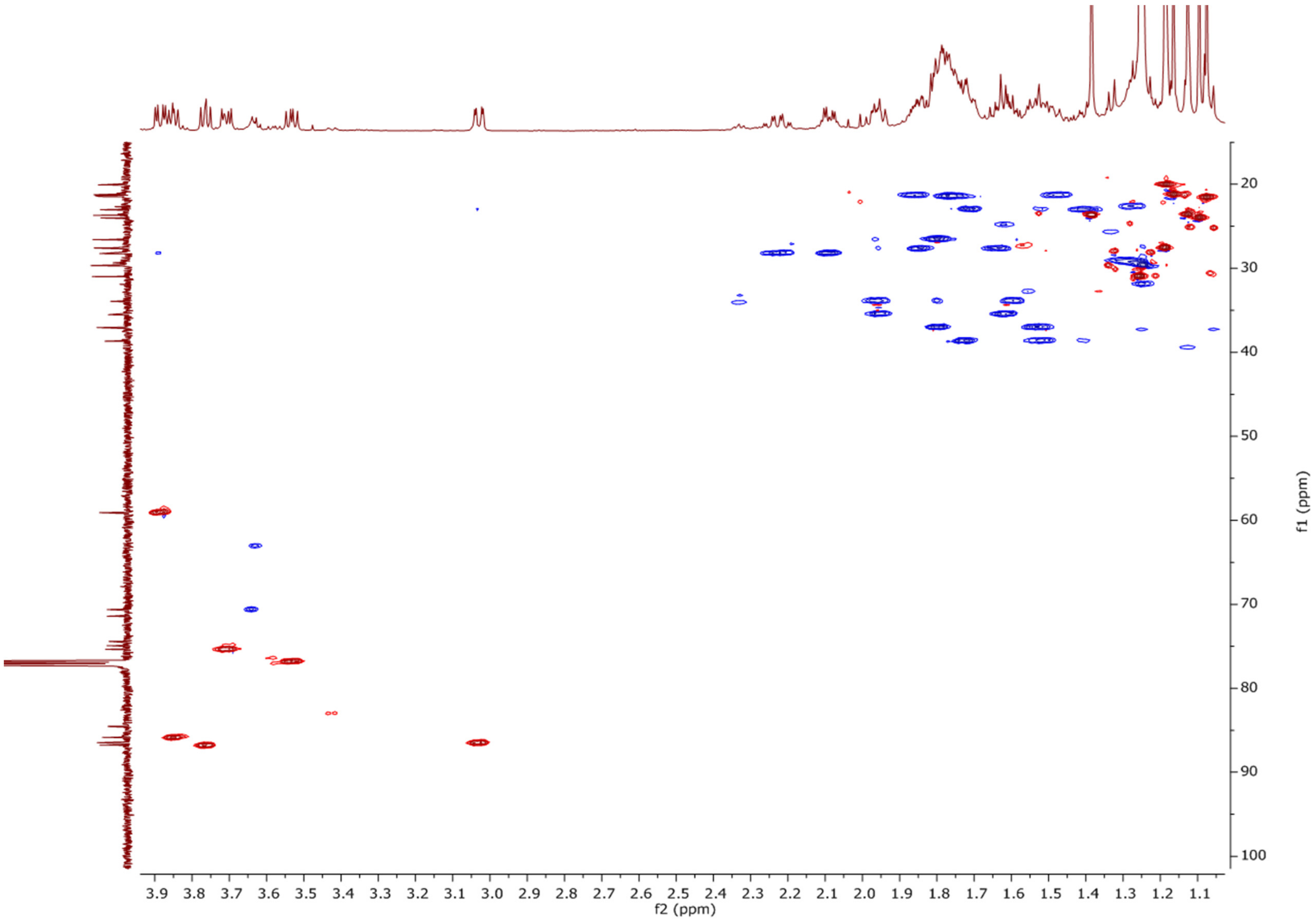

Figure S22. HSQC-DEPT NMR spectrum (600MHz, $CDCl_3$, 303K) of compound **4**

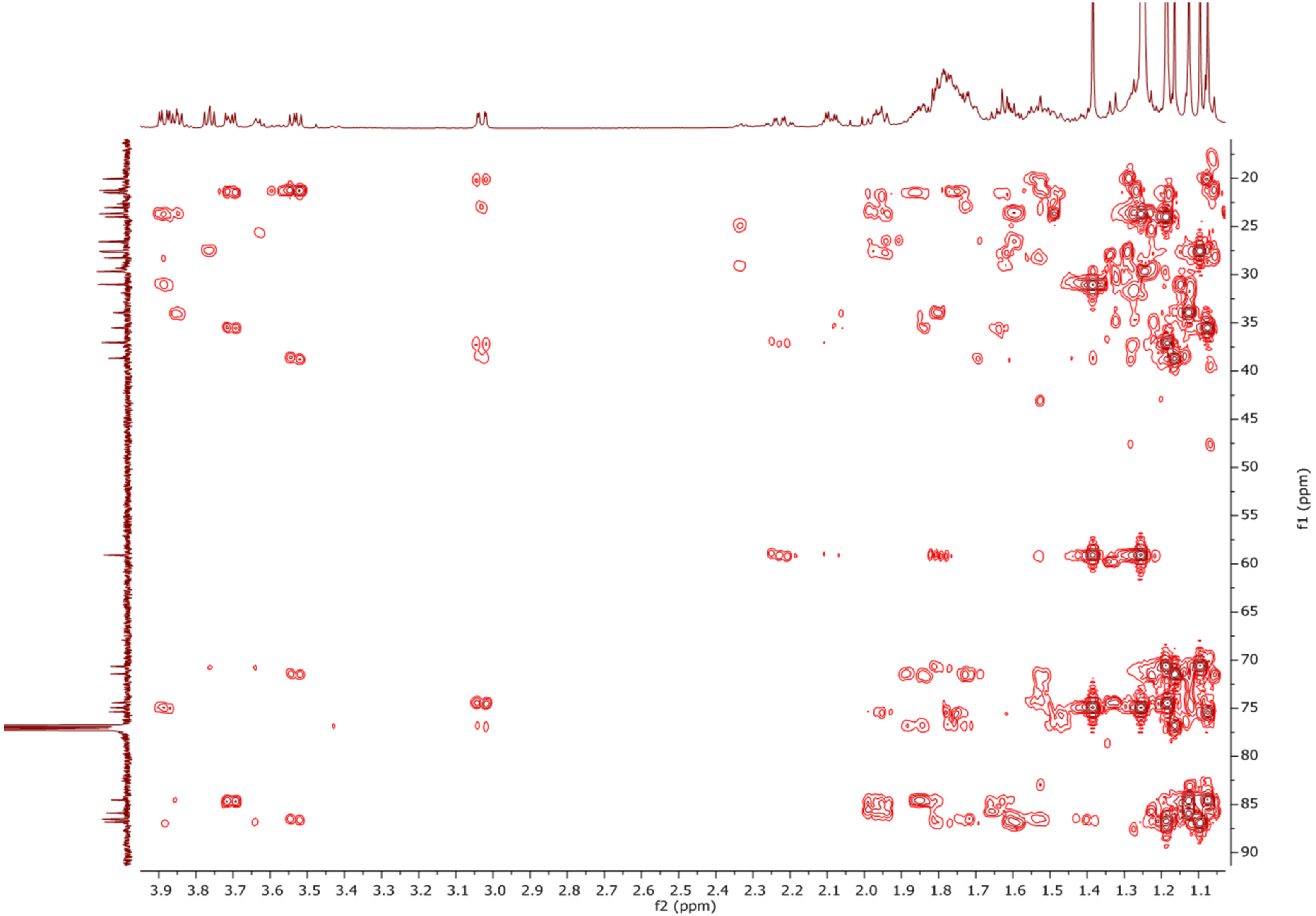

Figure S23. HMBC NMR spectrum (600MHz, $CDCl_3$, 303K) of compound **4**

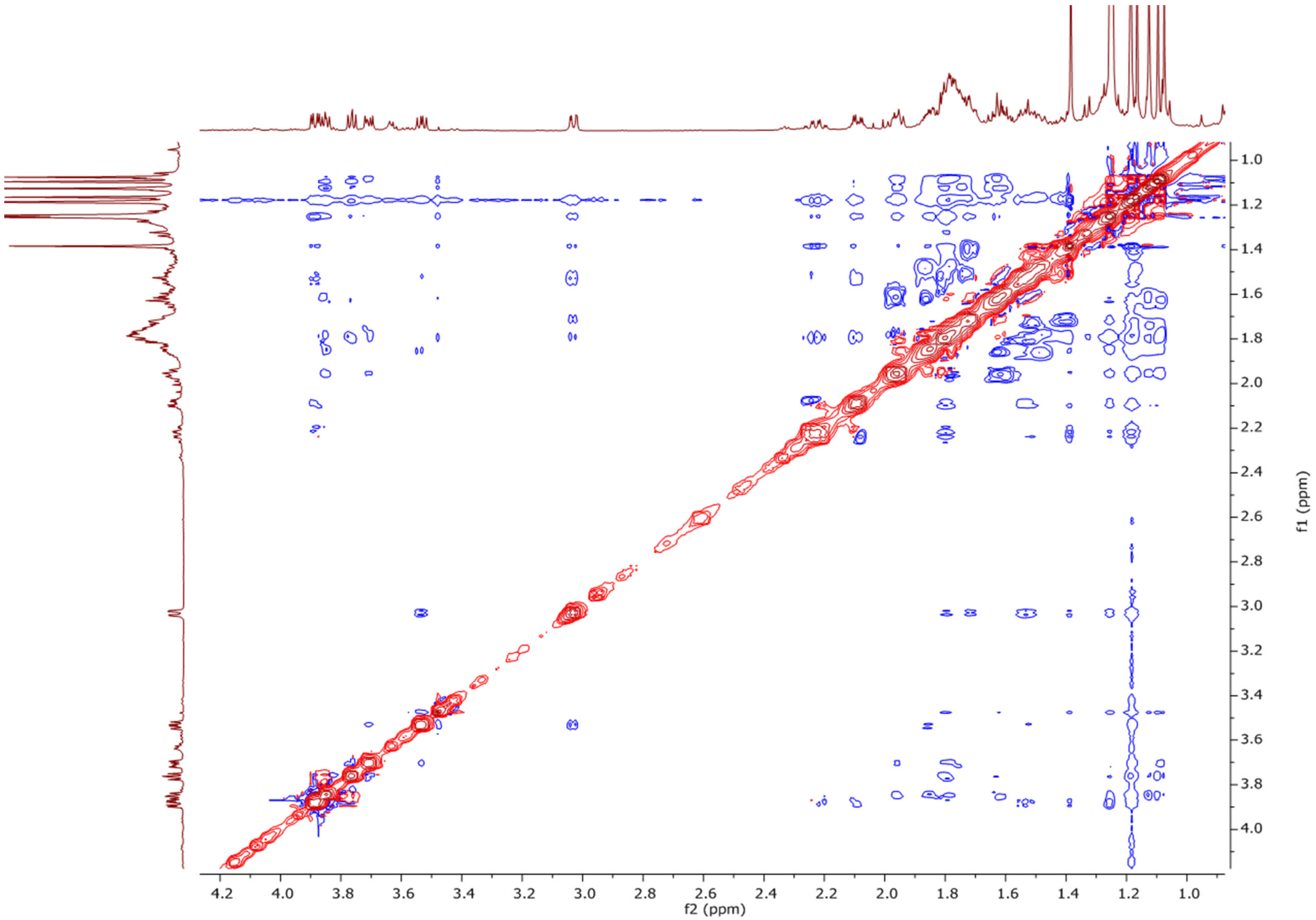

Figure S24. ROESY NMR spectrum (600MHz, $CDCl_3$, 303K) of compound **4**

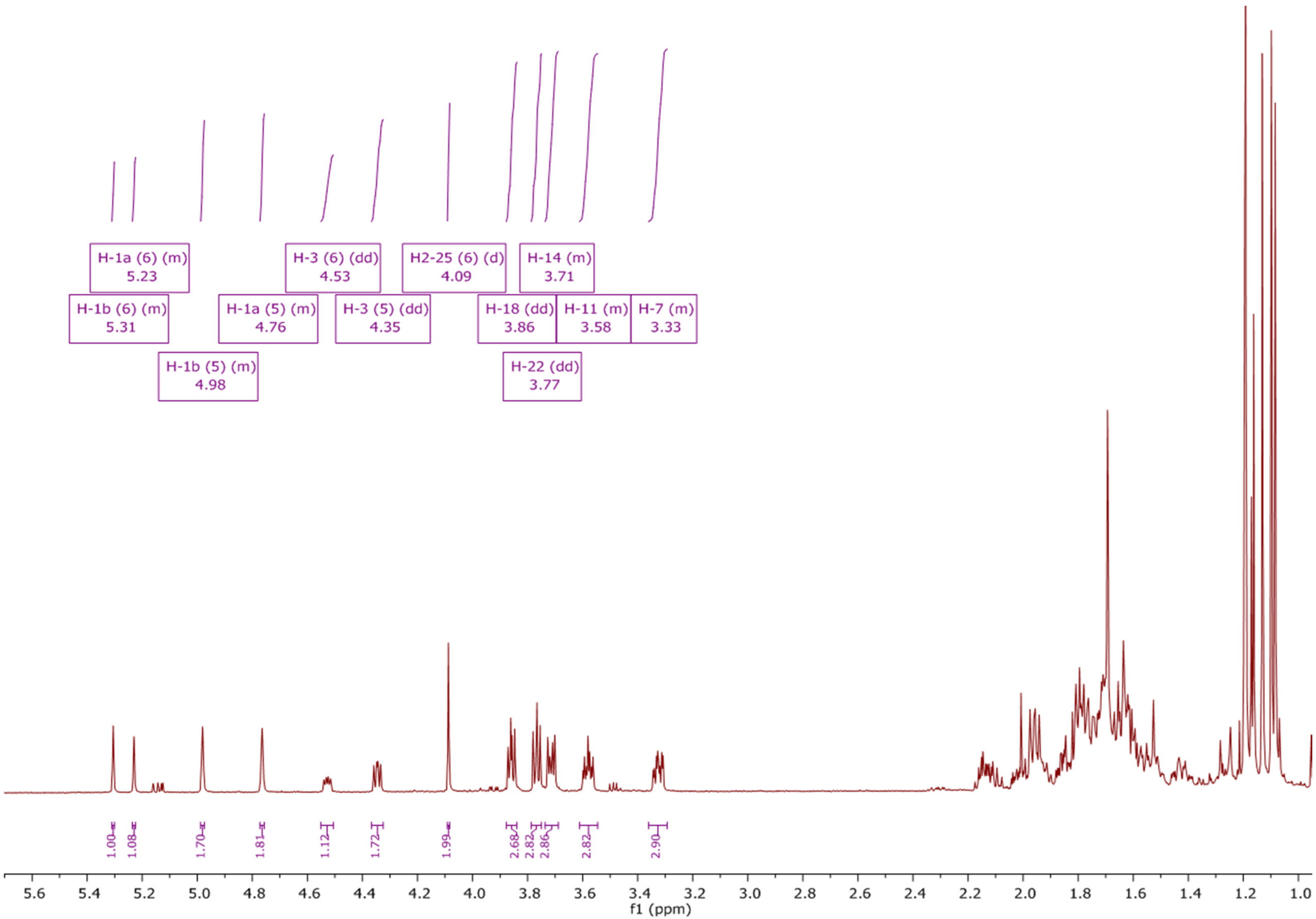

Figure S25. ^{1}H-NMR spectrum (600MHz, $CDCl_3$, 303K) of compounds **5** and **6**

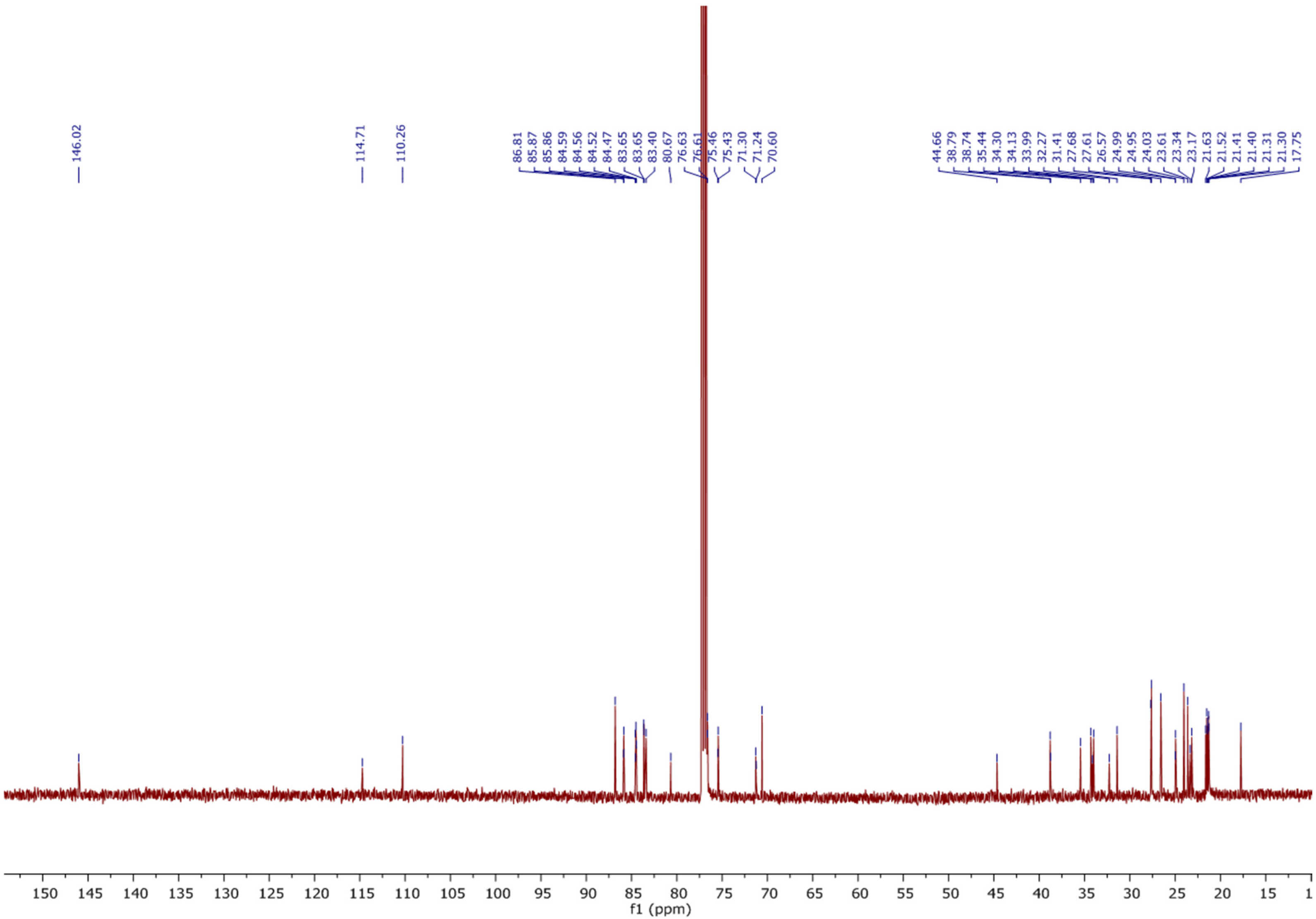

Figure S26. ^{13}C-NMR spectrum (150MHz, $CDCl_3$, 303K) of compounds **5** and **6**

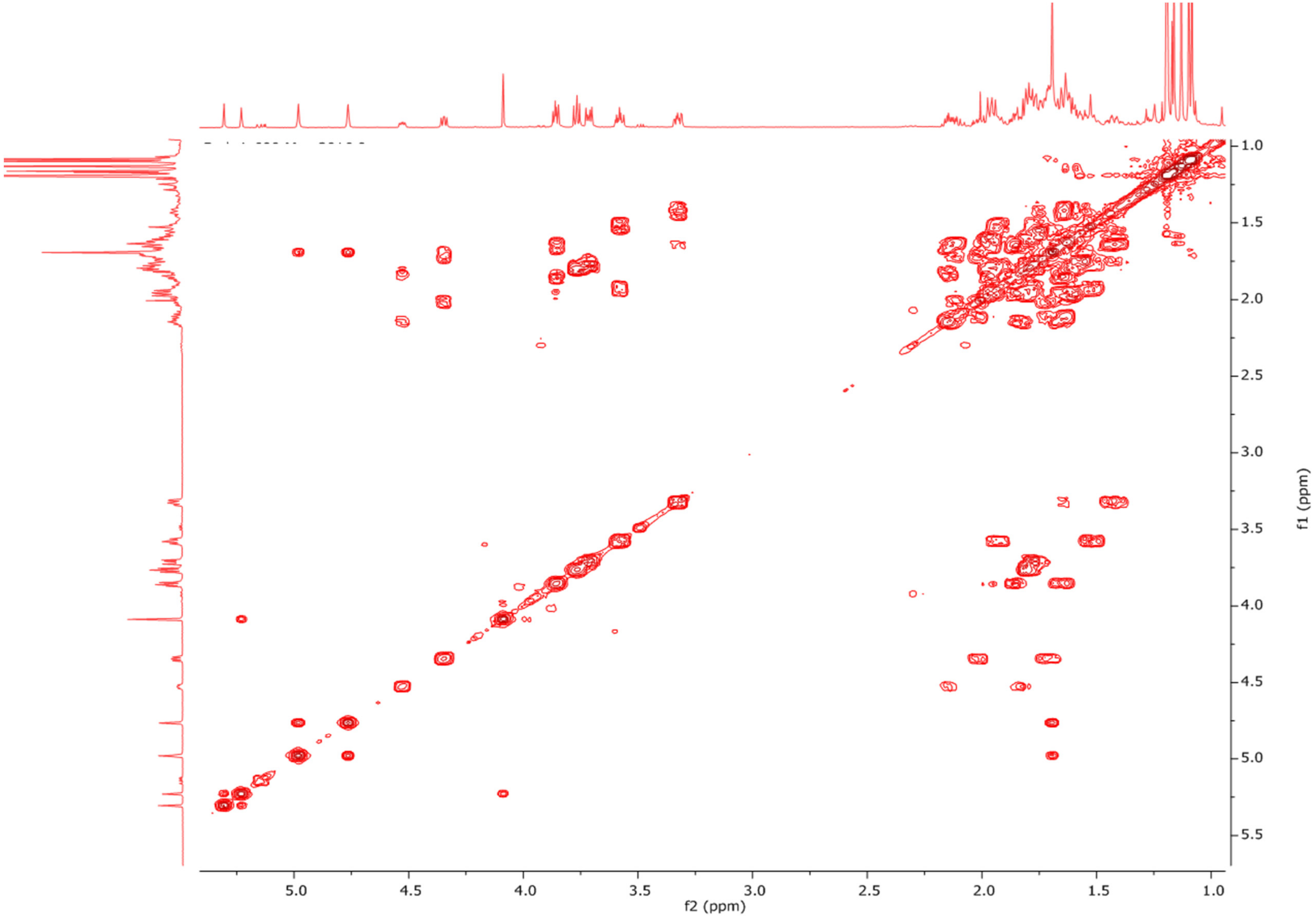

Figure S27. ^{1}H-^{1}H COSY NMR spectrum (600MHz, $CDCl_3$, 303K) of compounds **5** and **6**

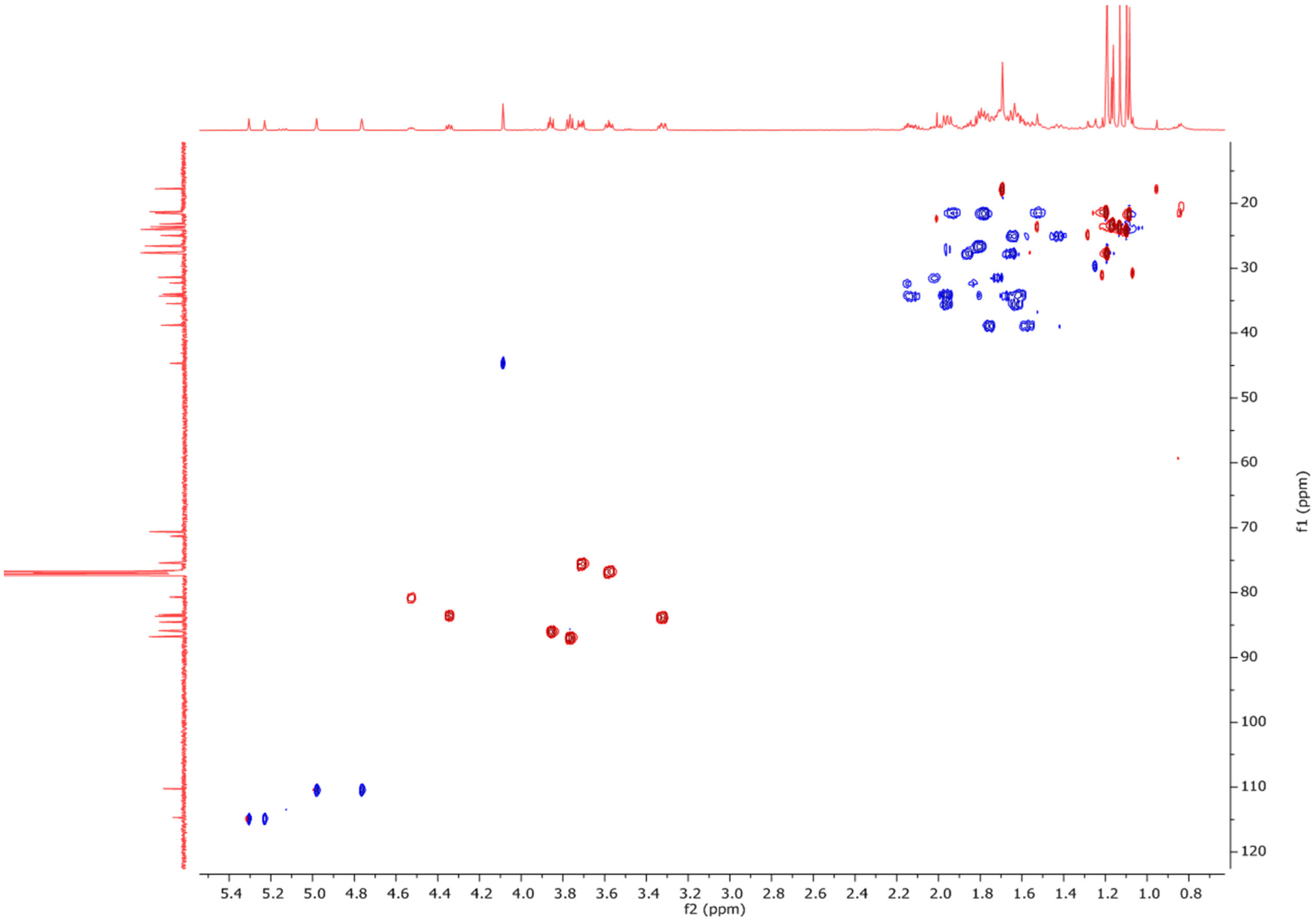

Figure S28. HSQC-DEPT NMR spectrum (600MHz, $CDCl_3$, 303K) of compounds **5** and **6**

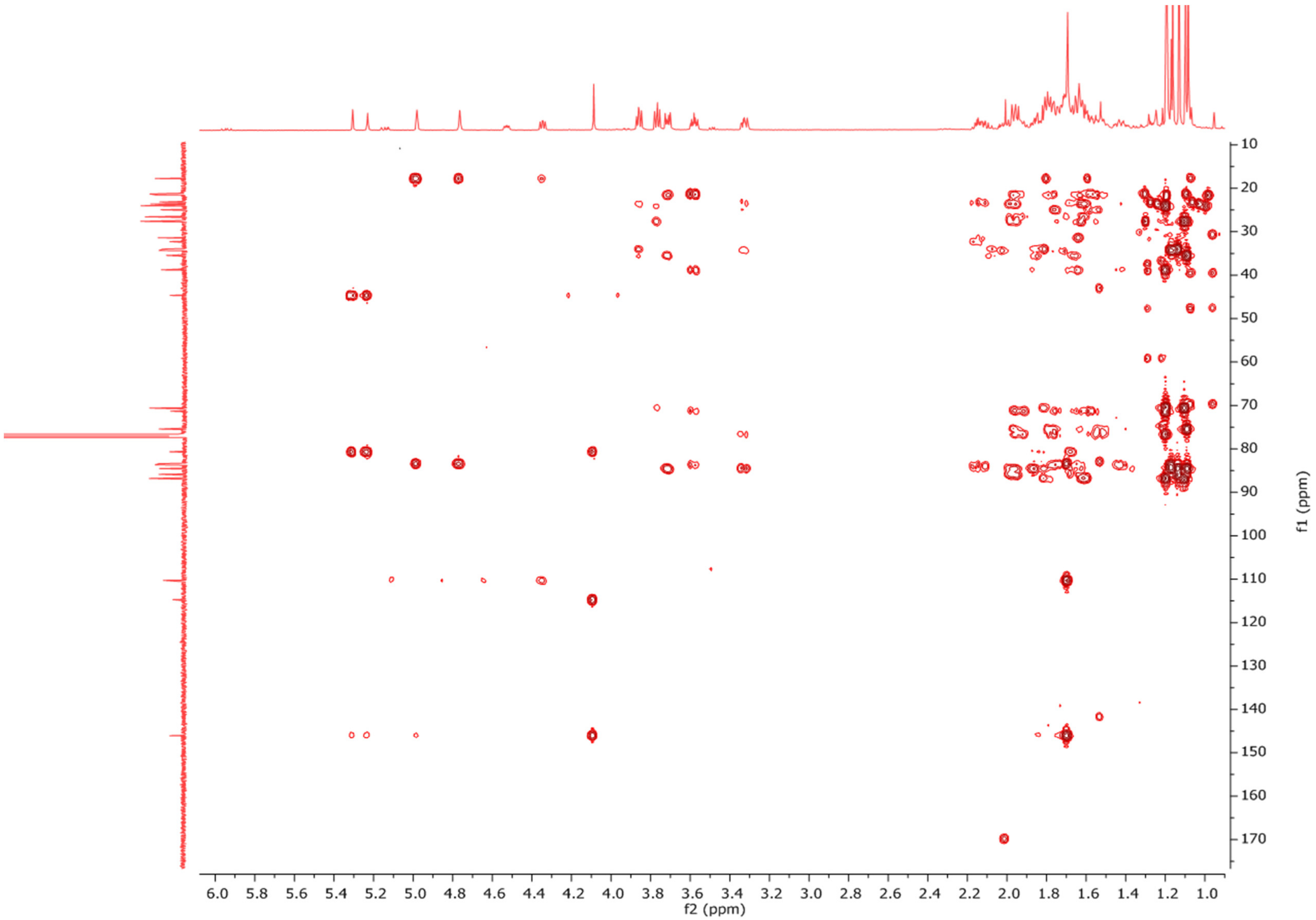

Figure S29. HMBC NMR spectrum (600MHz, $CDCl_3$, 303K) of compounds **5** and **6**

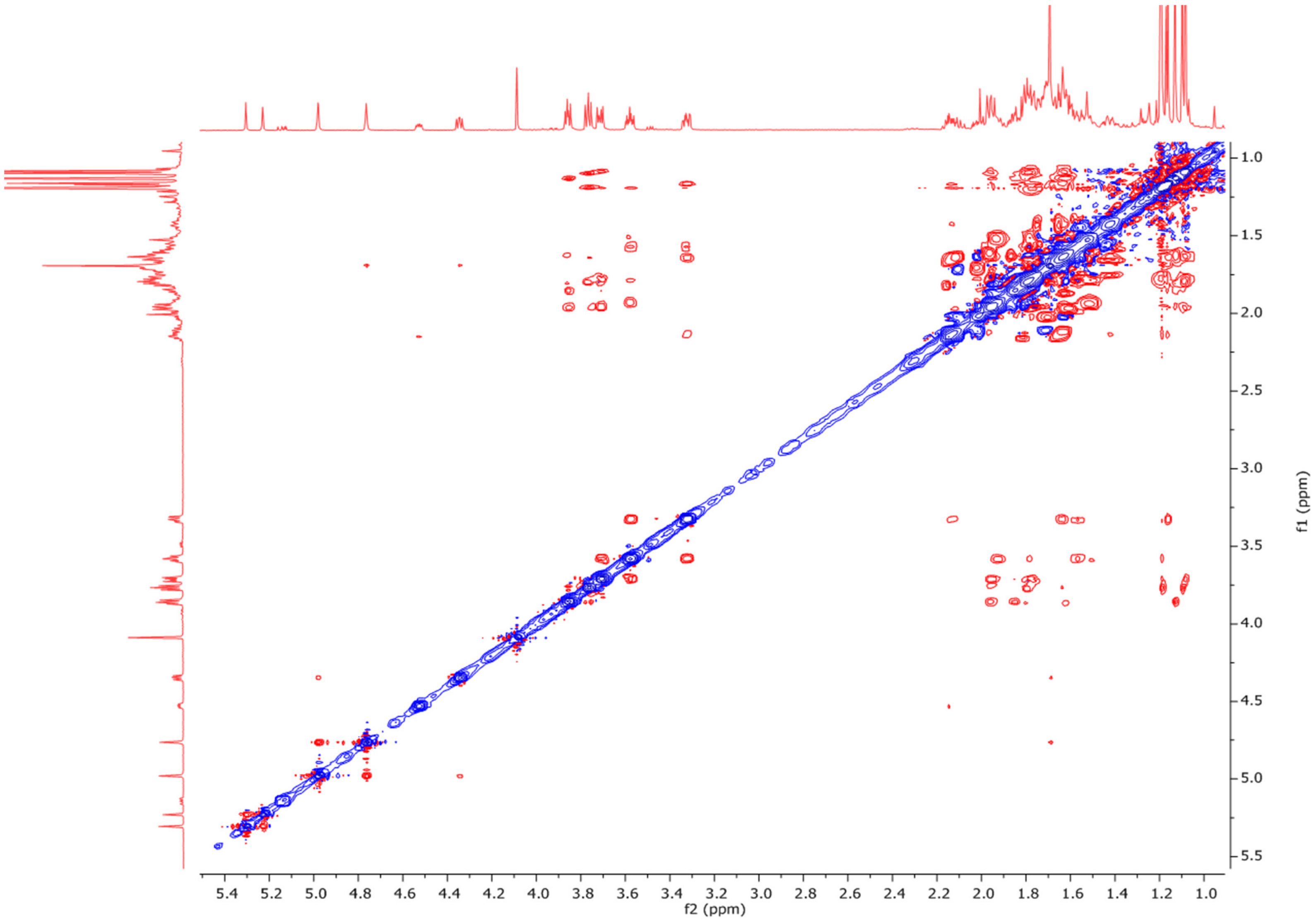

Figure S30. ROESY NMR spectrum (600MHz, $CDCl_3$, 303K) of compounds **5** and **6**

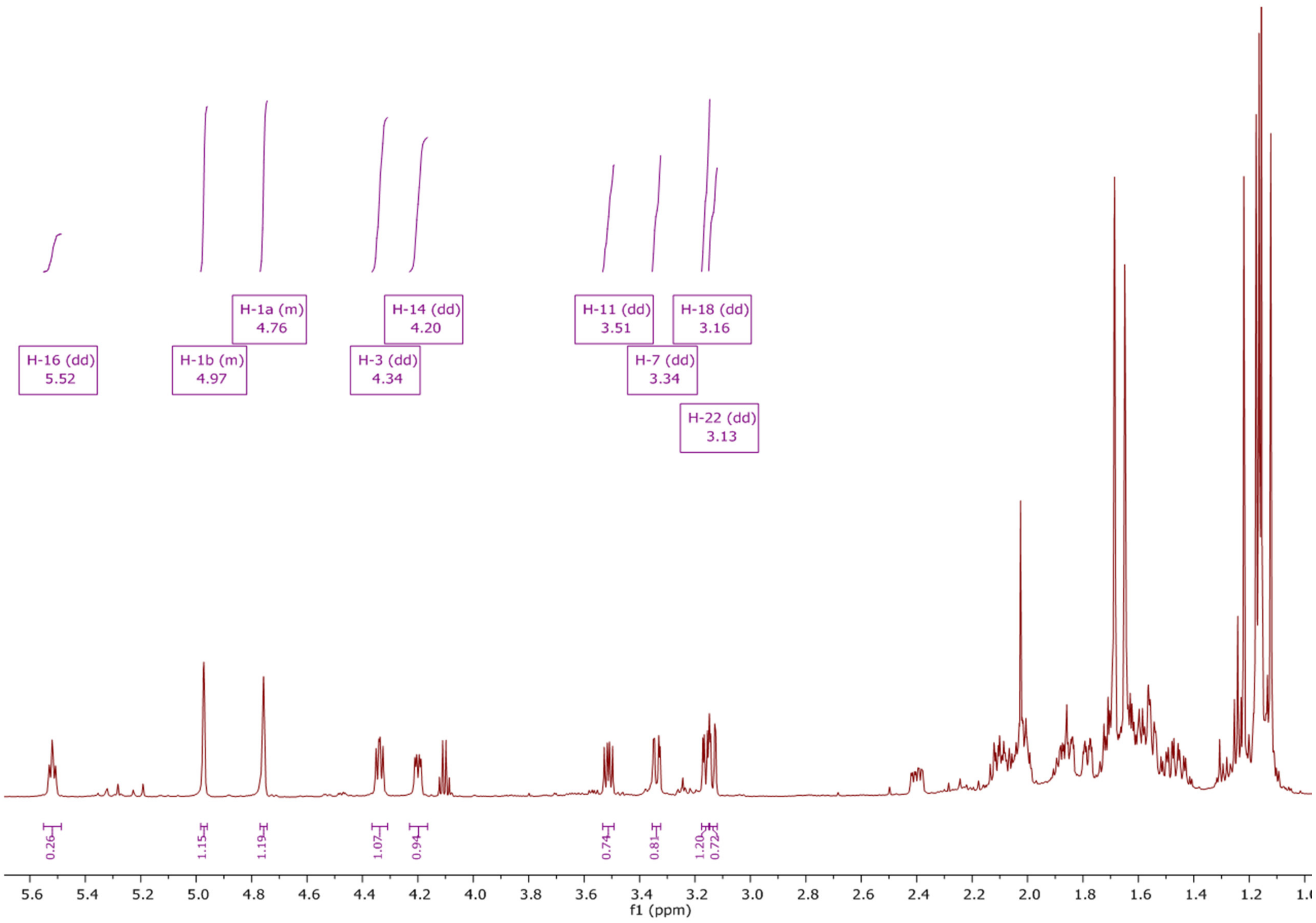

Figure S31. ^{1}H-NMR spectrum (600MHz, $CDCl_3$, 303K) of compound **7**

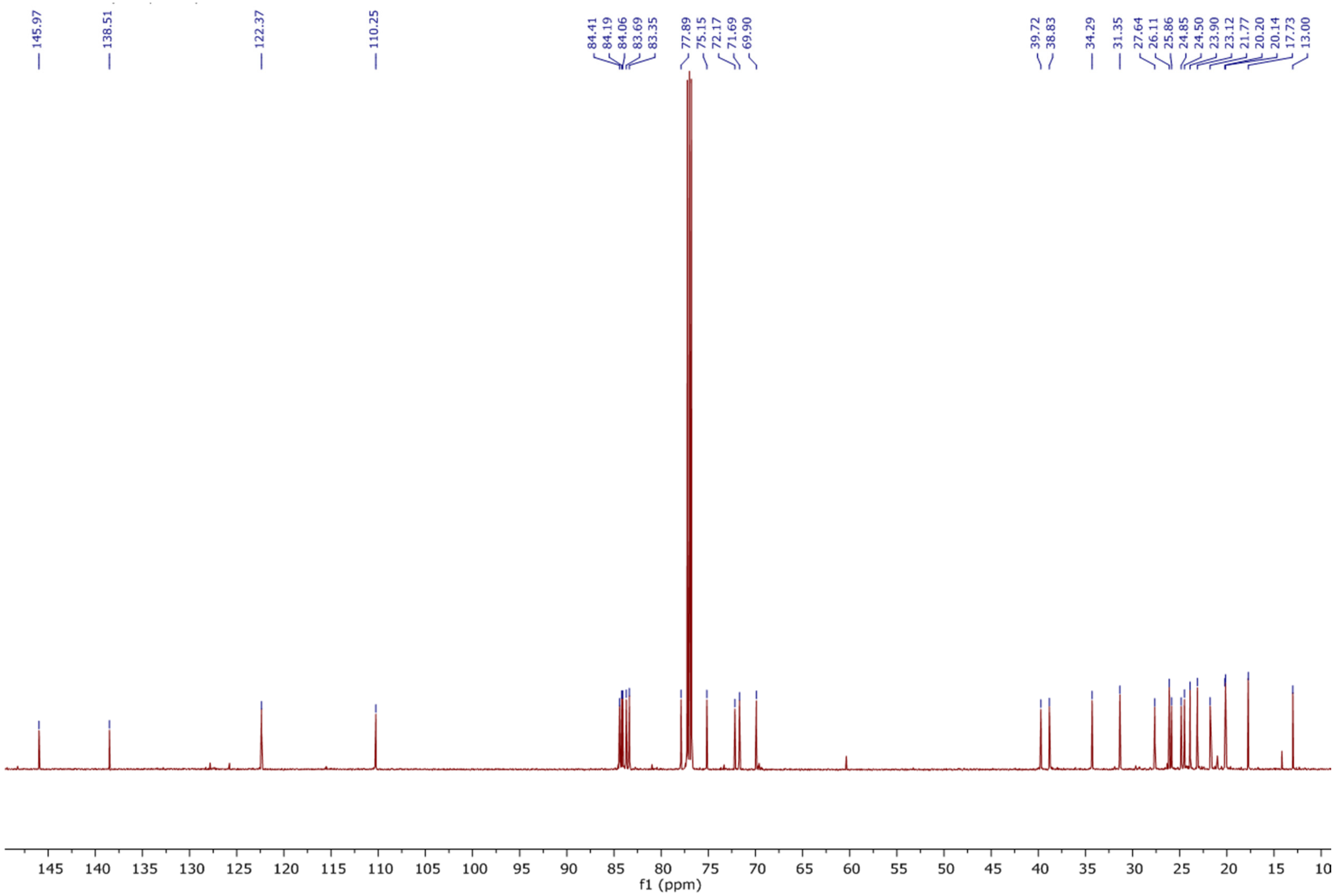

Figure S32. ^{13}C-NMR spectrum (150MHz, $CDCl_3$, 303K) of compound **7**

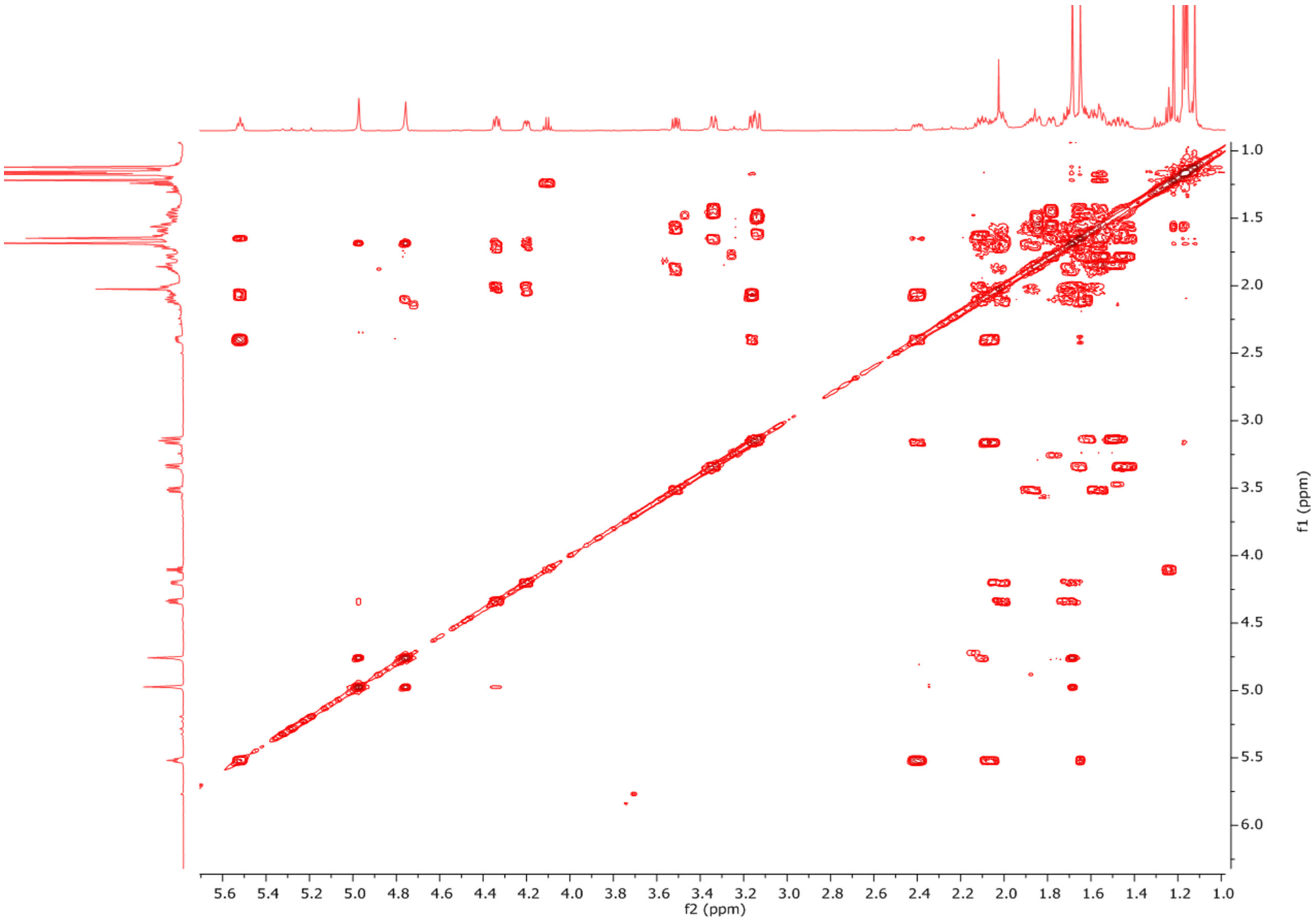

Figure S33. ^1H-^1H COSY NMR spectrum (600MHz, $CDCl_3$, 303K) of compound **7**

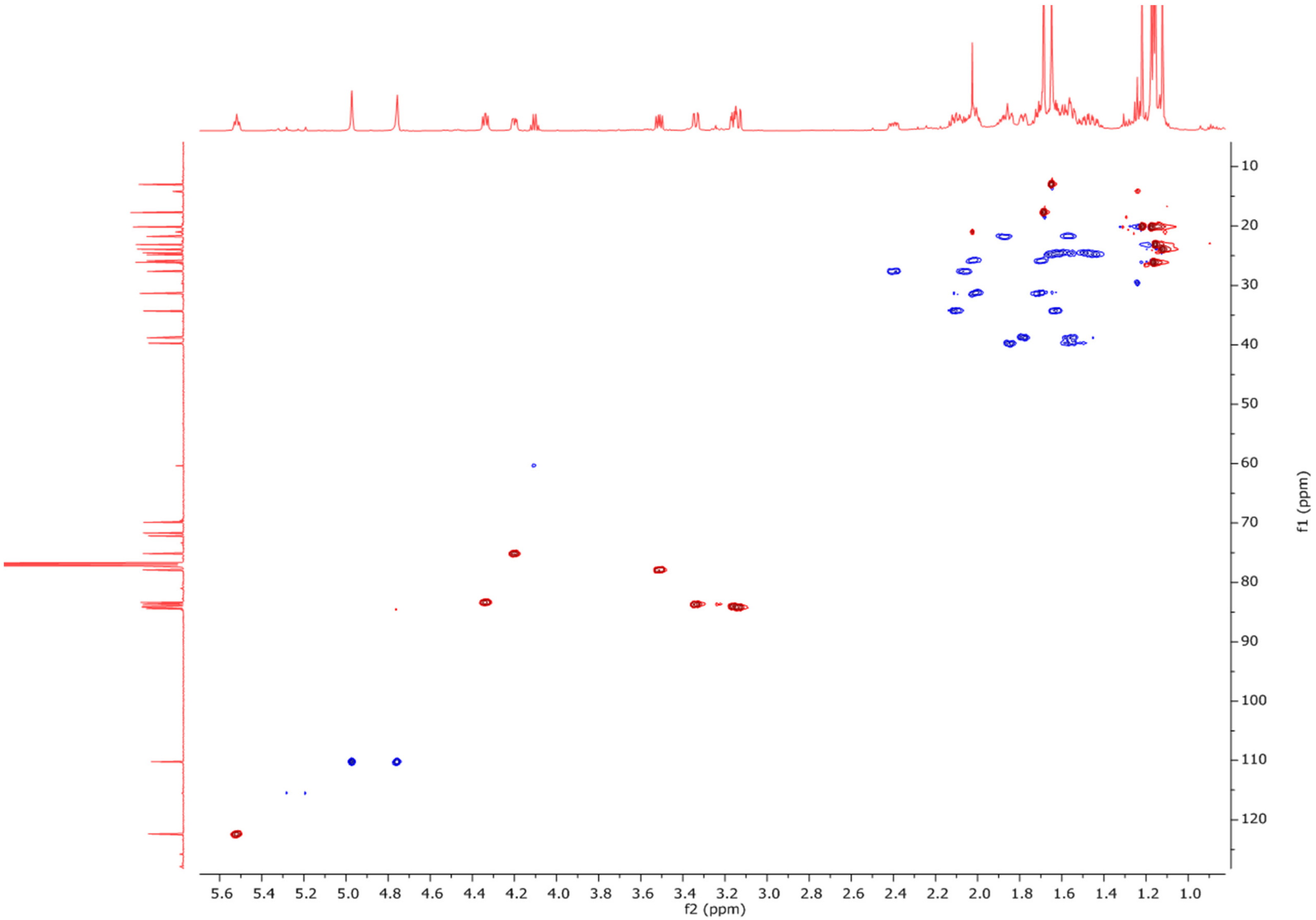

Figure S34. HSQC-DEPT NMR spectrum (600MHz, $CDCl_3$, 303K) of compound **7**

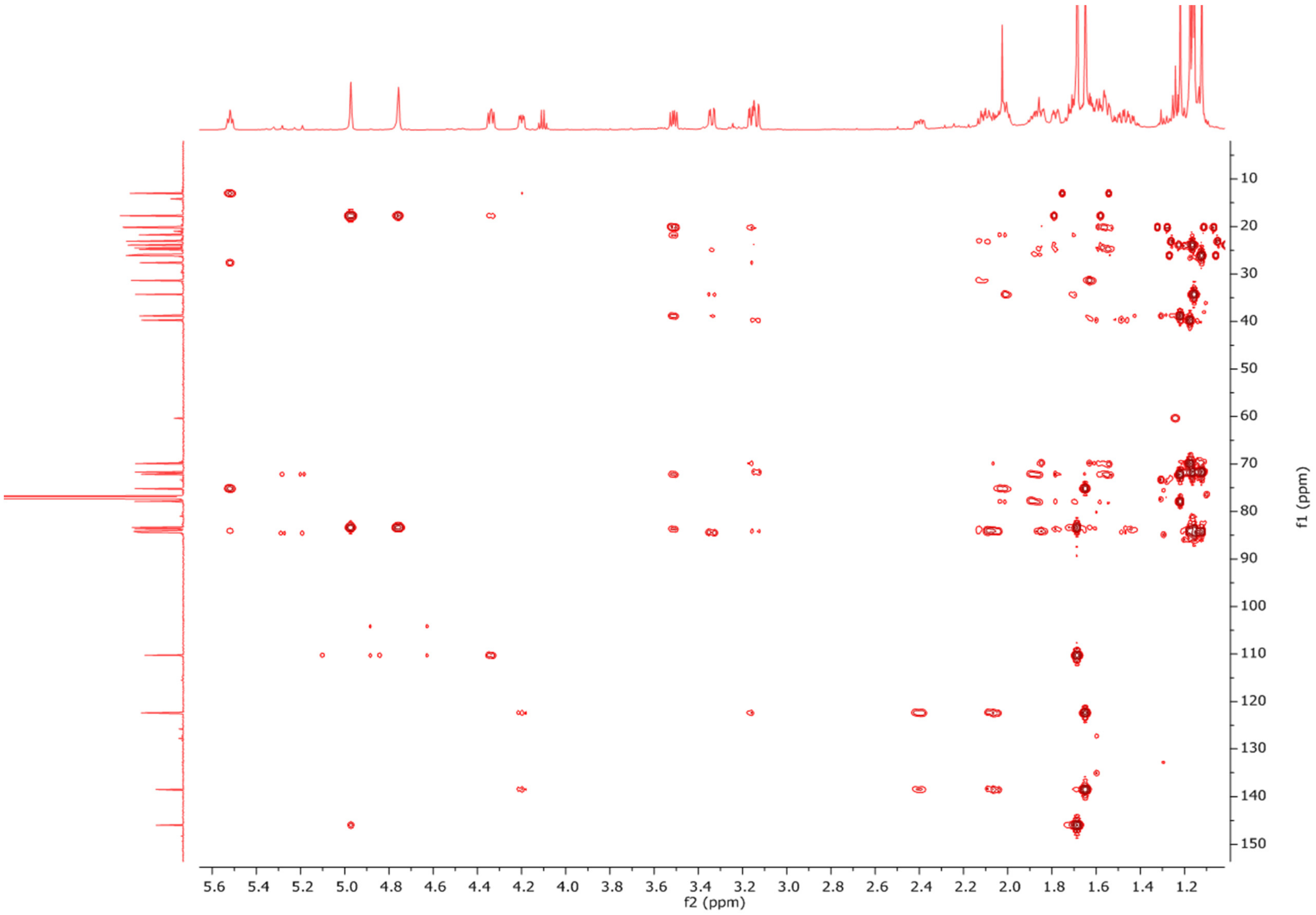

Figure S35. HMBC NMR spectrum (600MHz, $CDCl_3$, 303K) of compound **7**

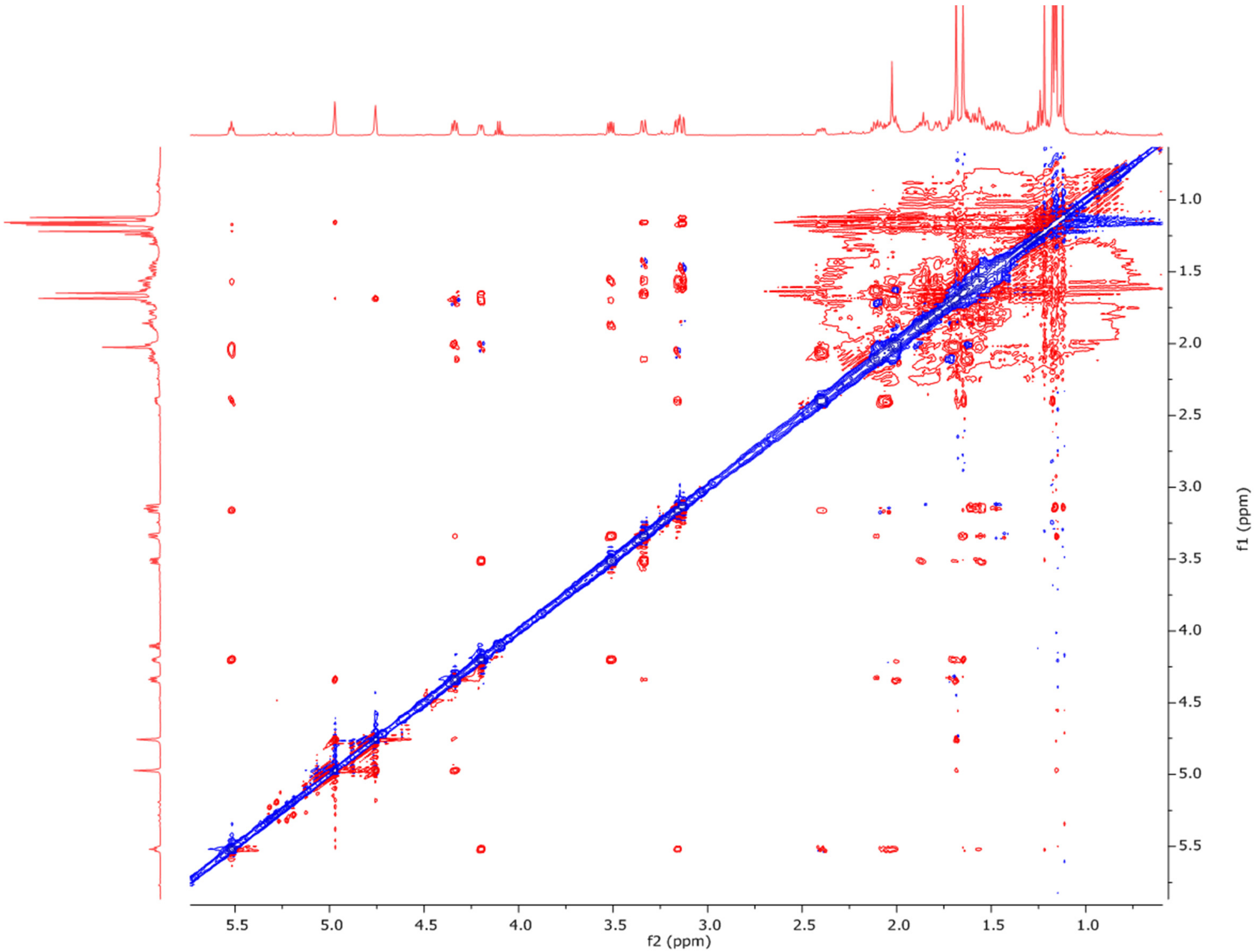

Figure S36. ROESY NMR spectrum (600MHz, $CDCl_3$, 303K) of compound **7**

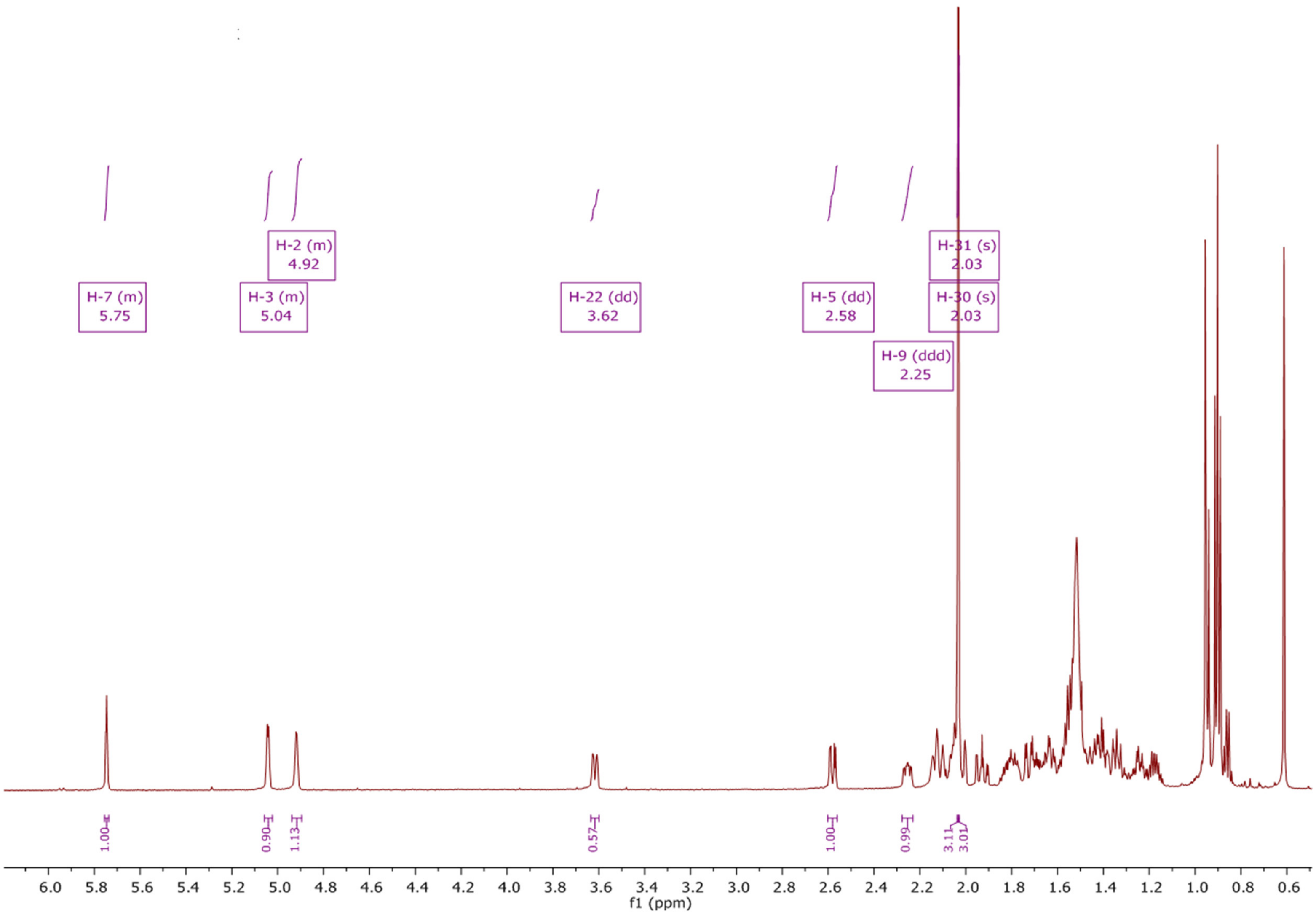

Figure S37. ^{1}H-NMR spectrum (600MHz, $CDCl_3$, 303K) of compound **8**

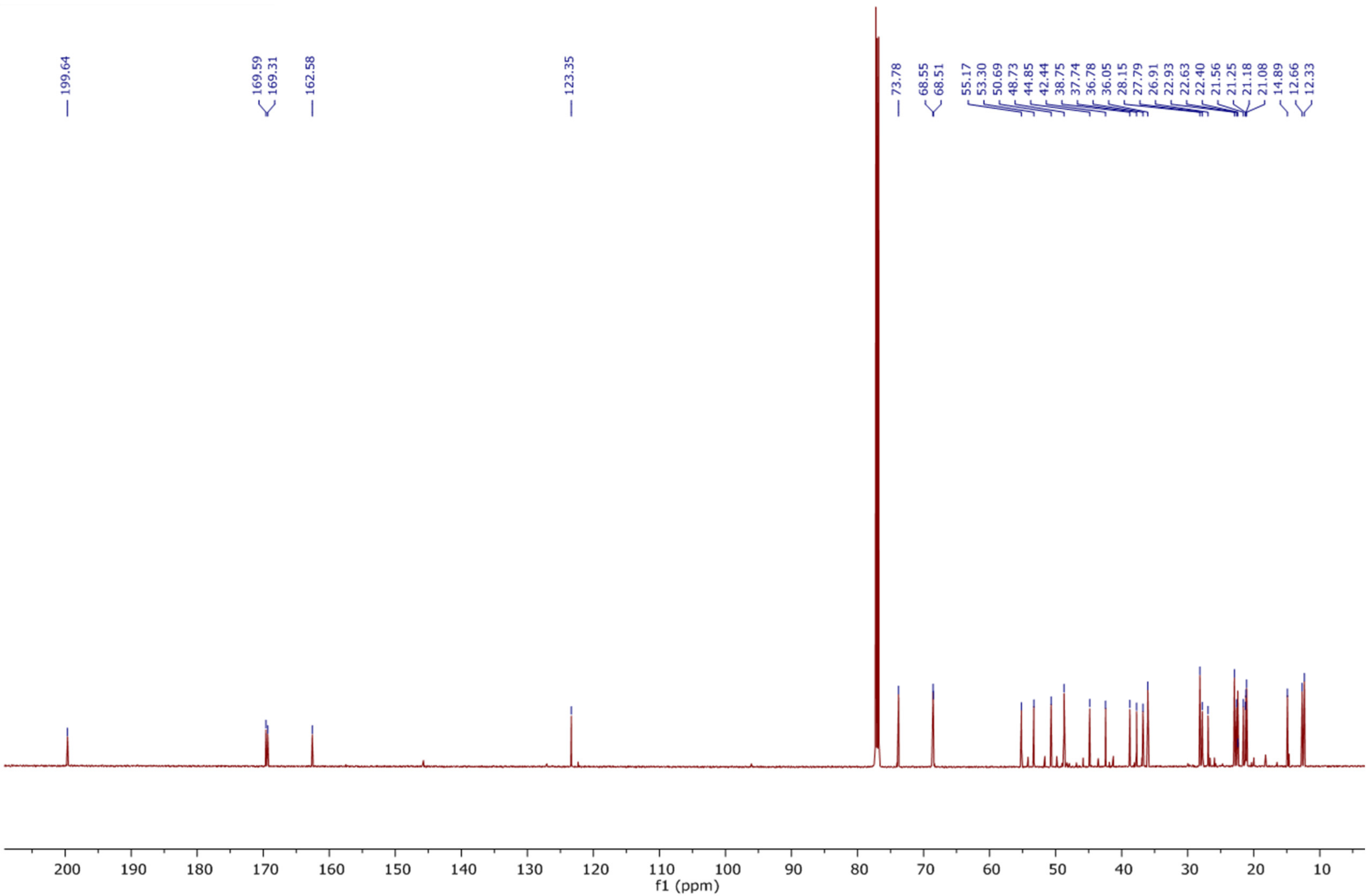

Figure S38. ^{13}C-NMR spectrum (150MHz, $CDCl_3$, 303K) of compound **8**

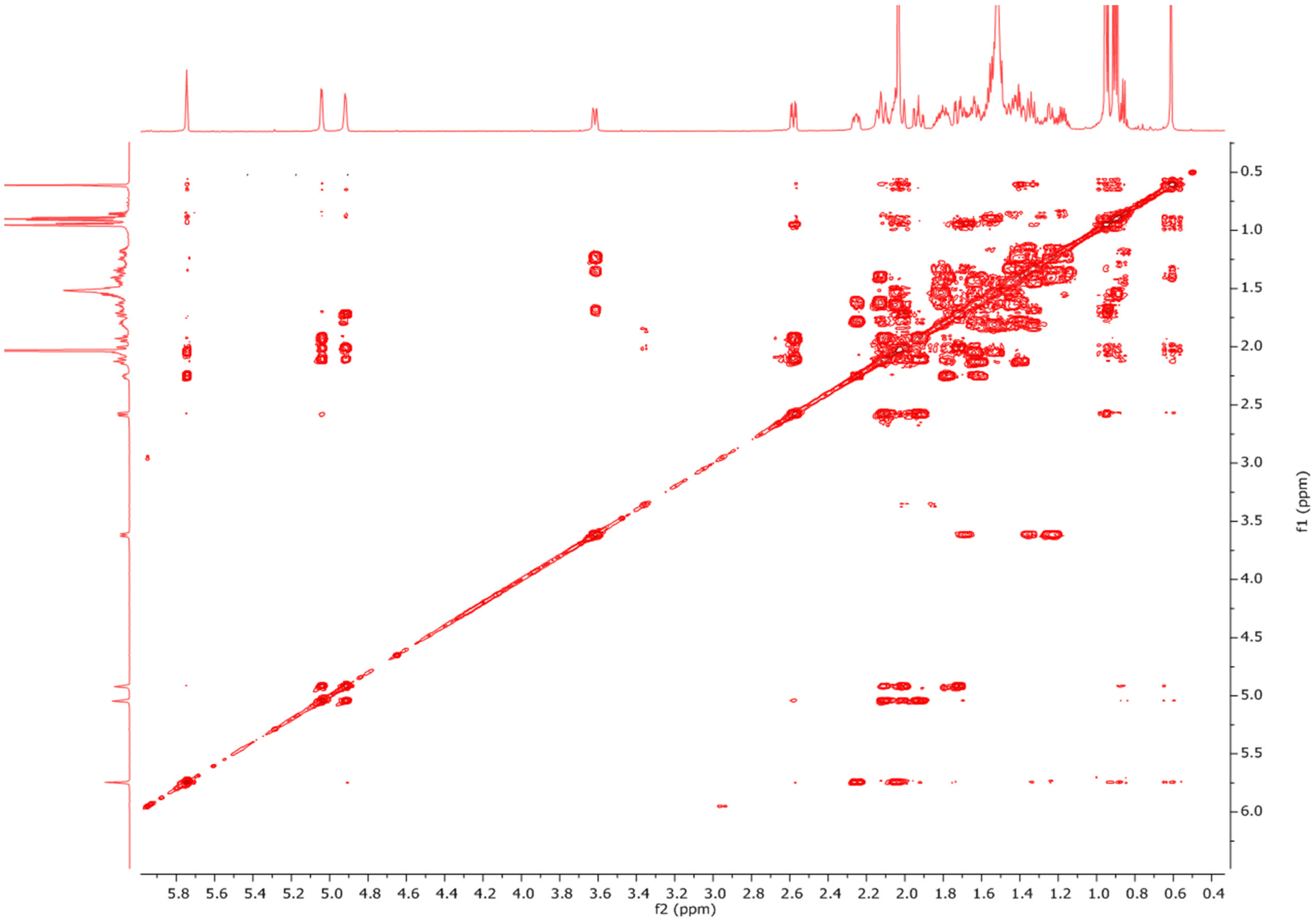

Figure S39. ^{1}H-^{1}H COSY NMR spectrum (600MHz, $CDCl_3$, 303K) of compound **8**

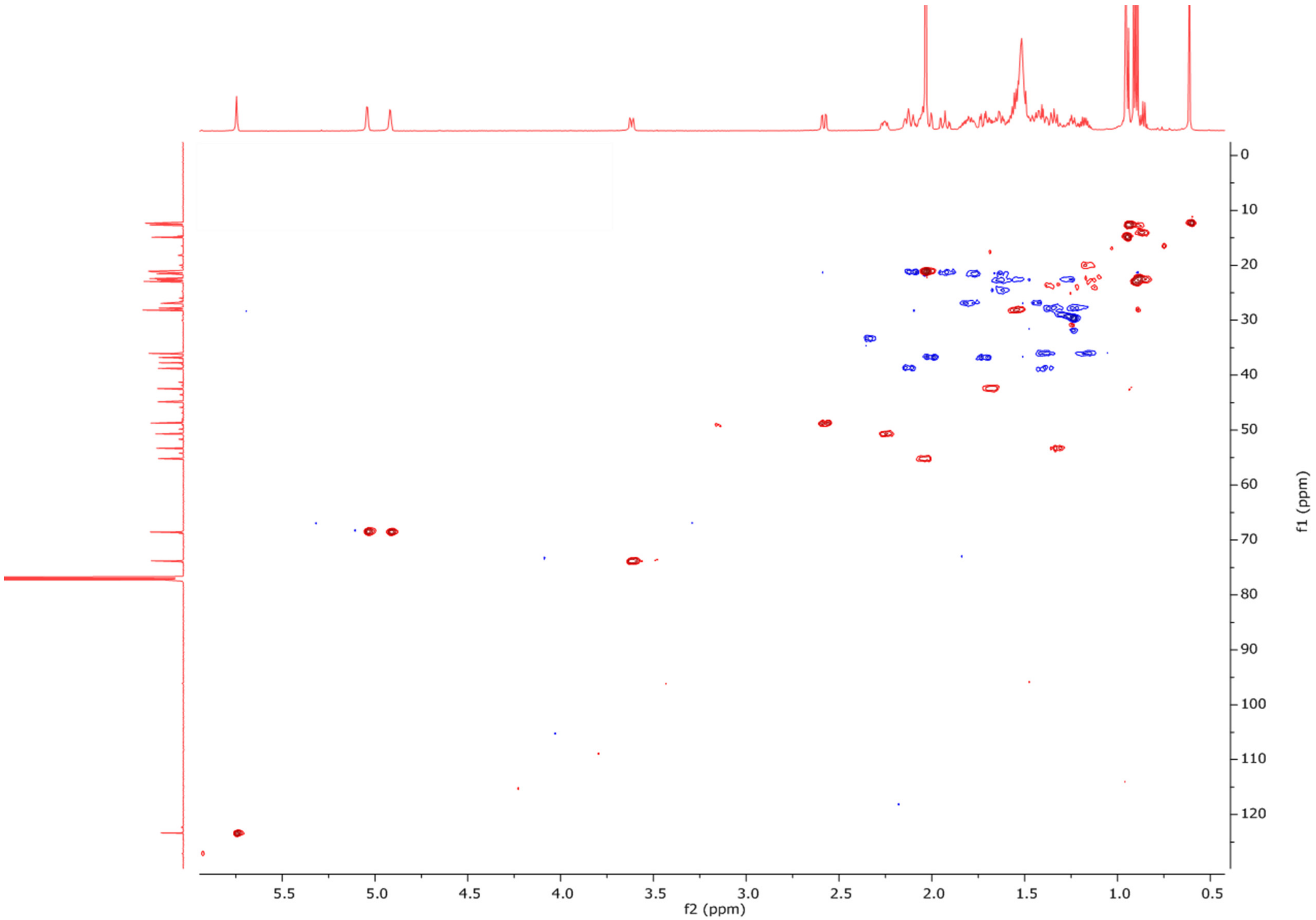

Figure S40. HSQC-DEPT NMR spectrum (600MHz, $CDCl_3$, 303K) of compound **8**

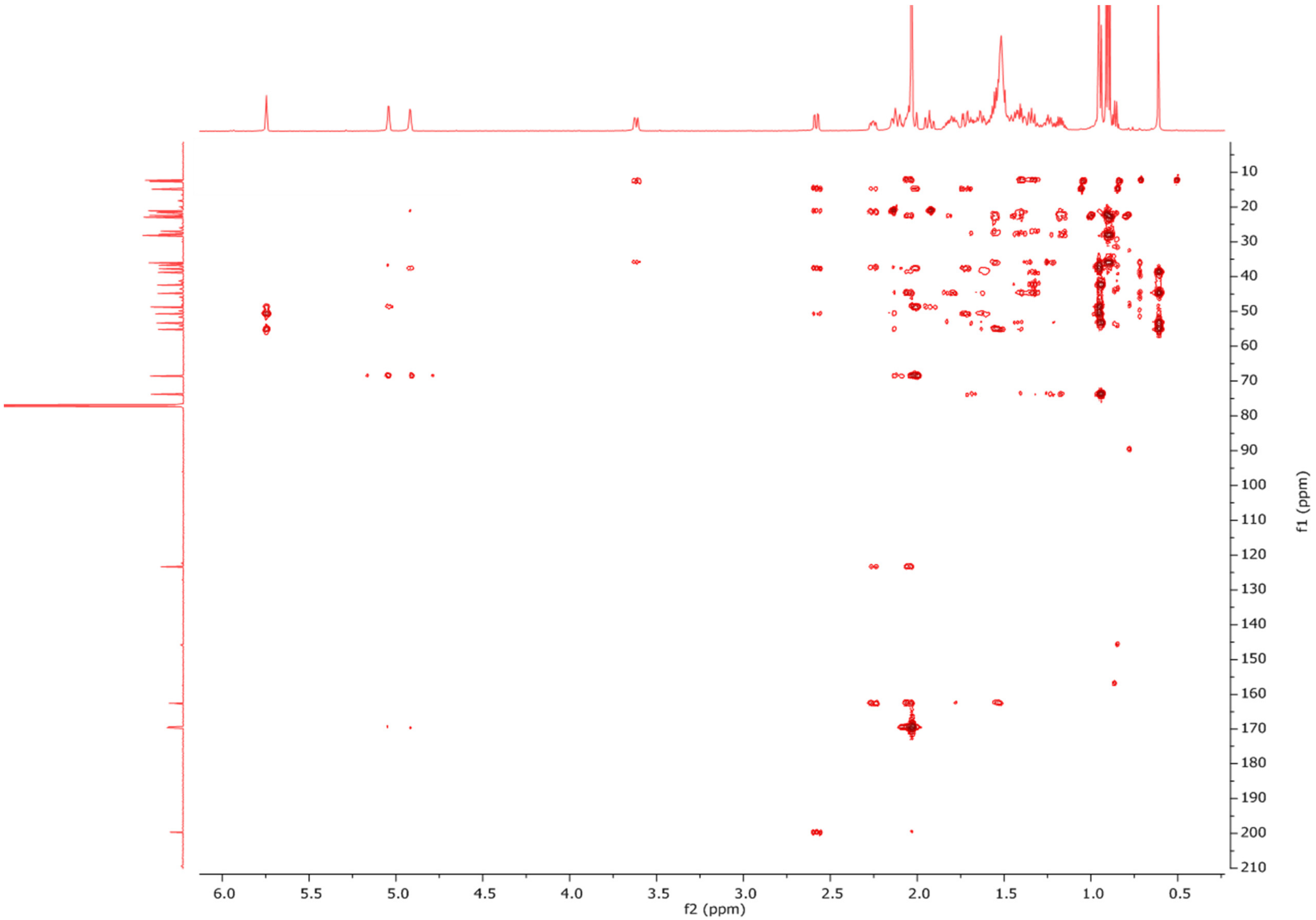

Figure S41. HMBC NMR spectrum (600MHz, $CDCl_3$, 303K) of compound **8**

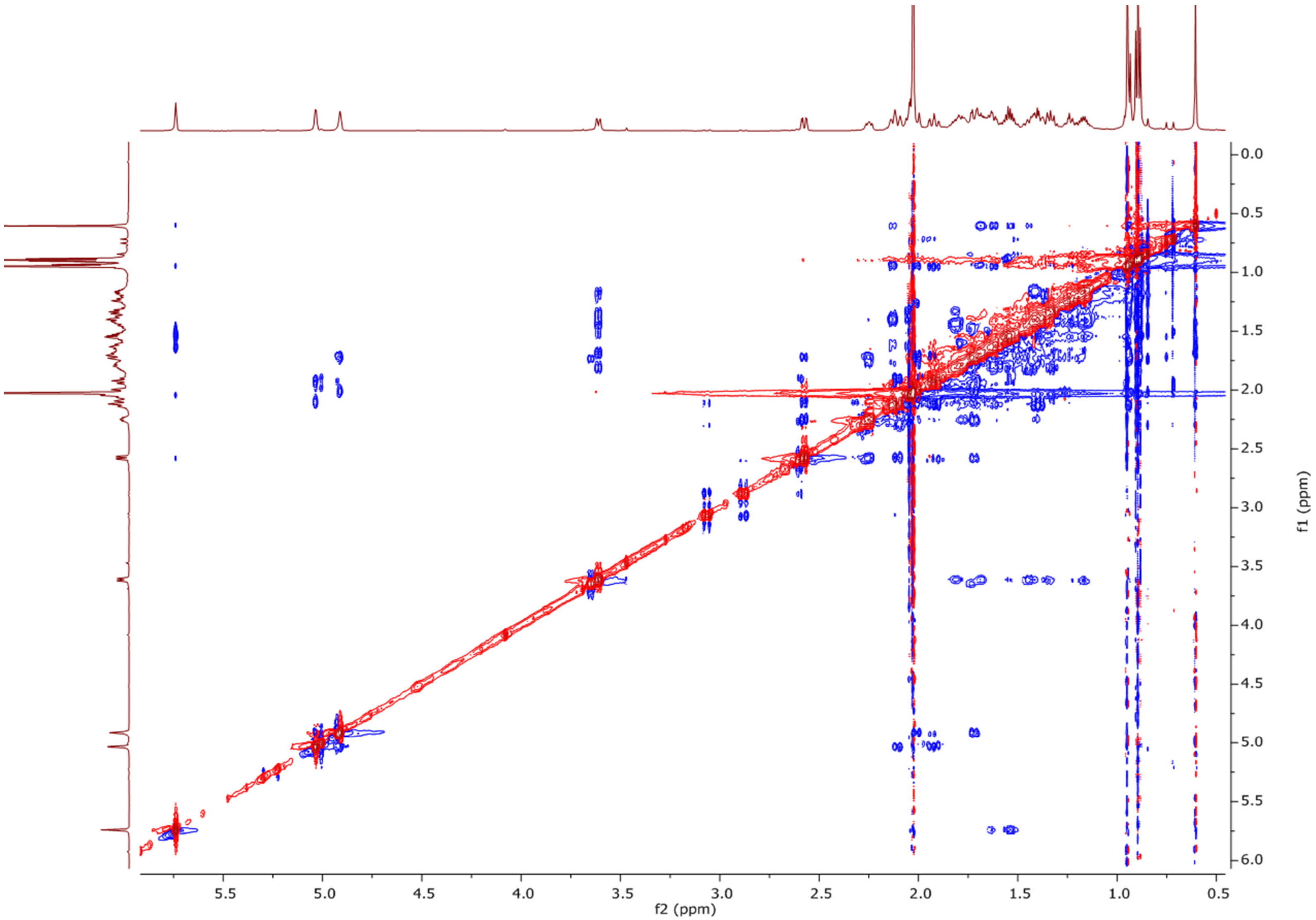

Figure S42. NOESY NMR spectrum (600MHz, $CDCl_3$, 303K) of compound **8**

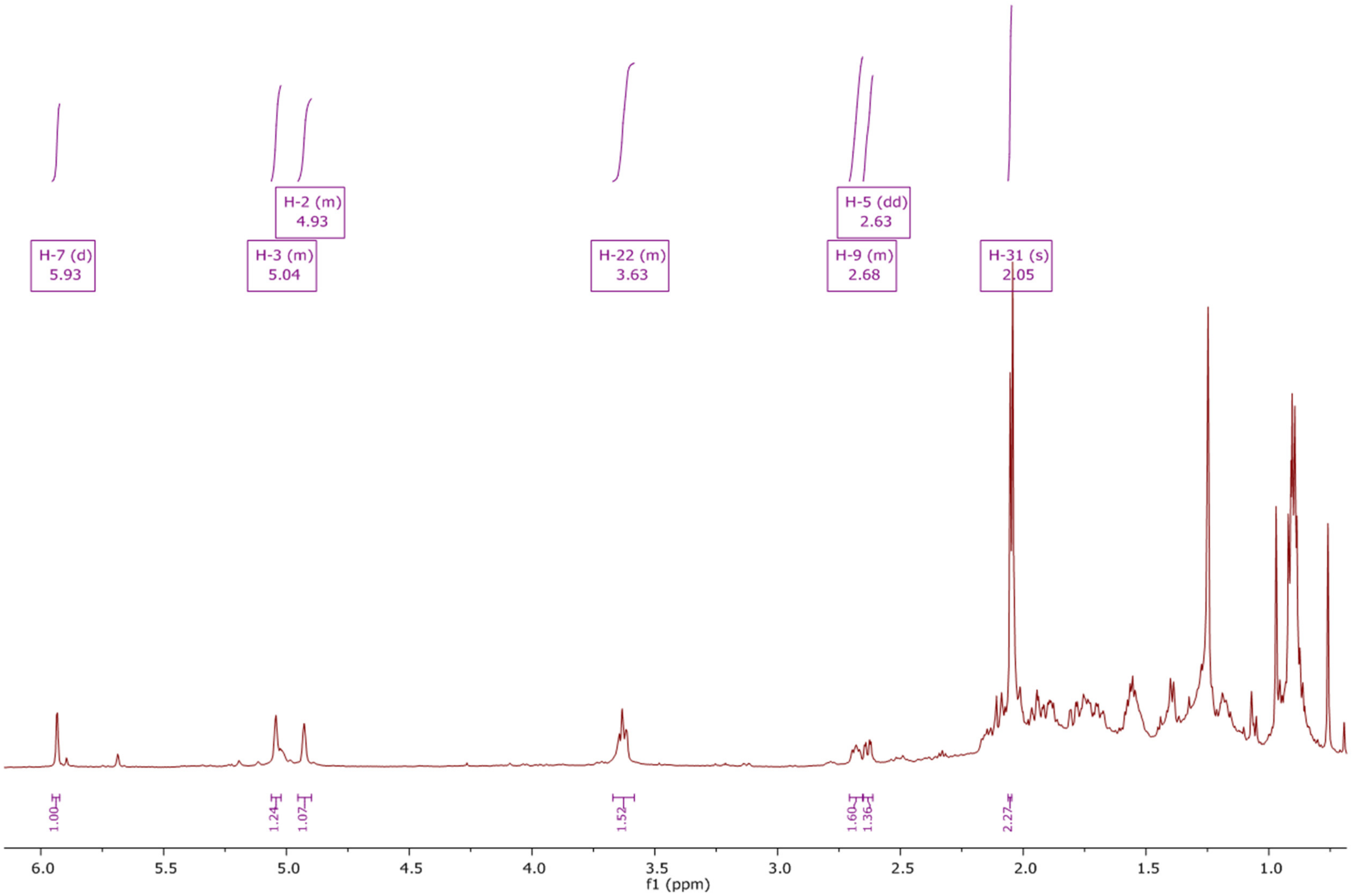

Figure S43. ^{1}H-NMR spectrum (600MHz, $CDCl_3$, 303K) of compound **9**

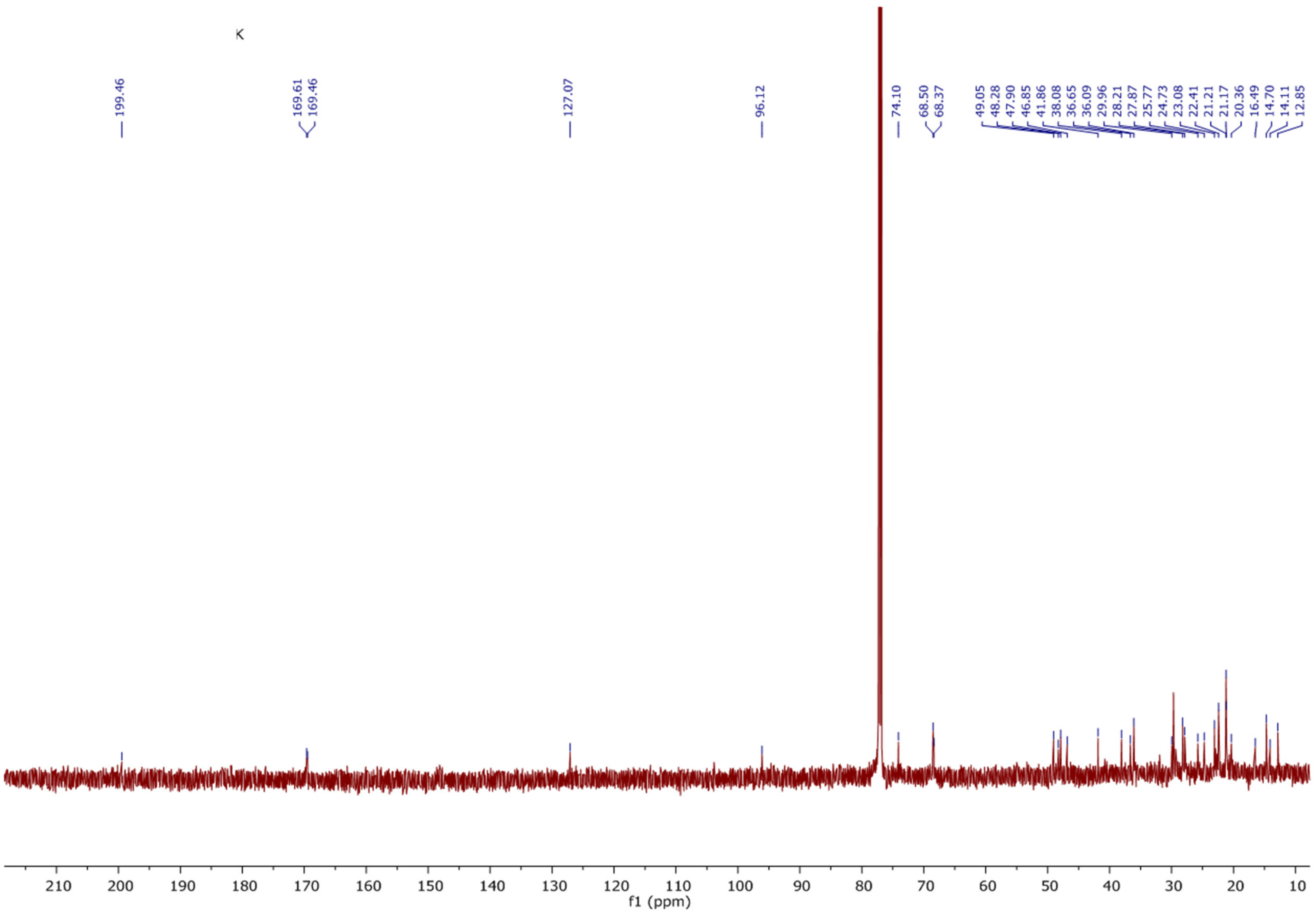

Figure S44. ^{13}C-NMR spectrum (150MHz, $CDCl_3$, 303K) of compound **9**

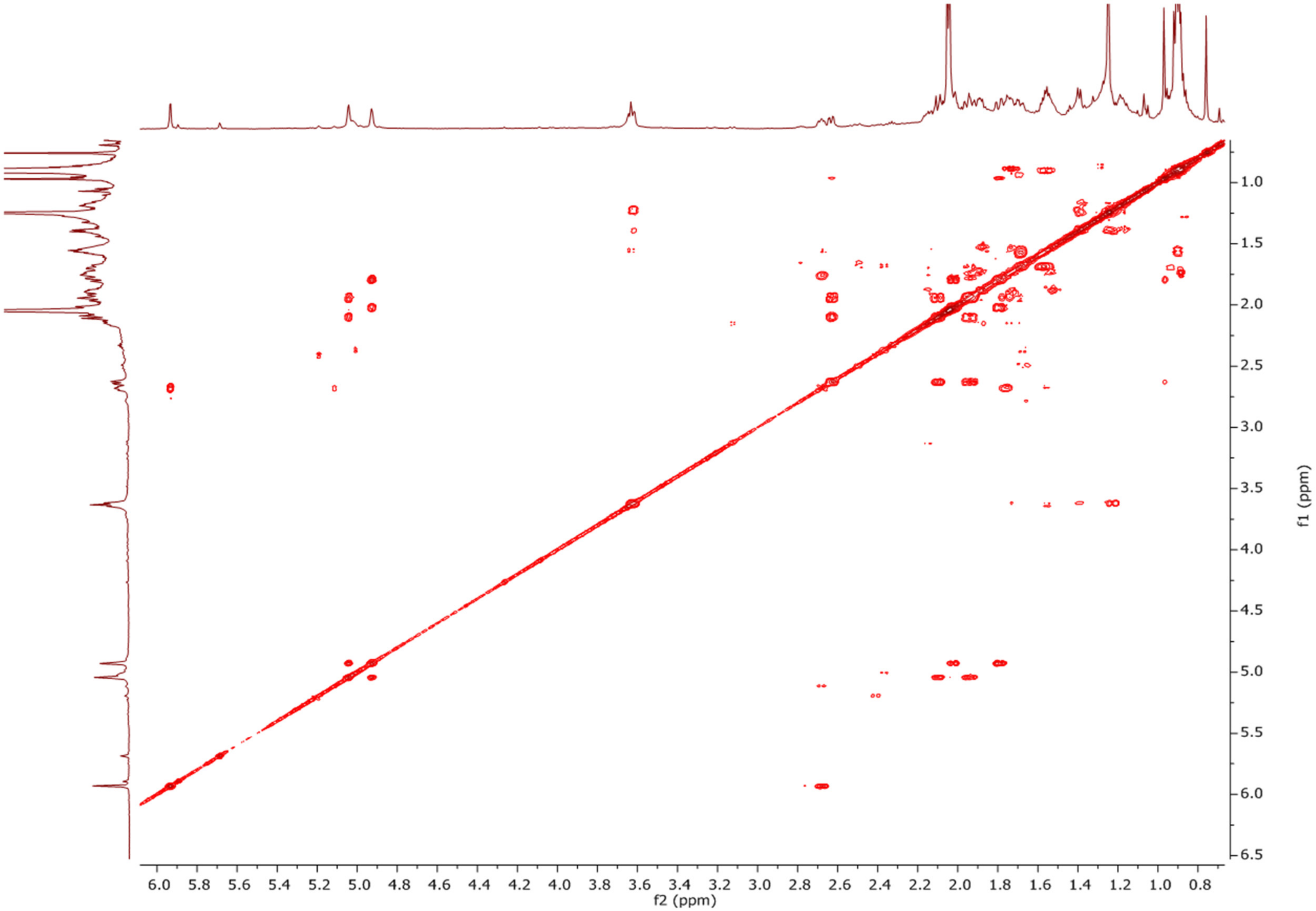

Figure S45. ^1H-^1H COSY NMR spectrum (600MHz, $CDCl_3$, 303K) of compound **9**

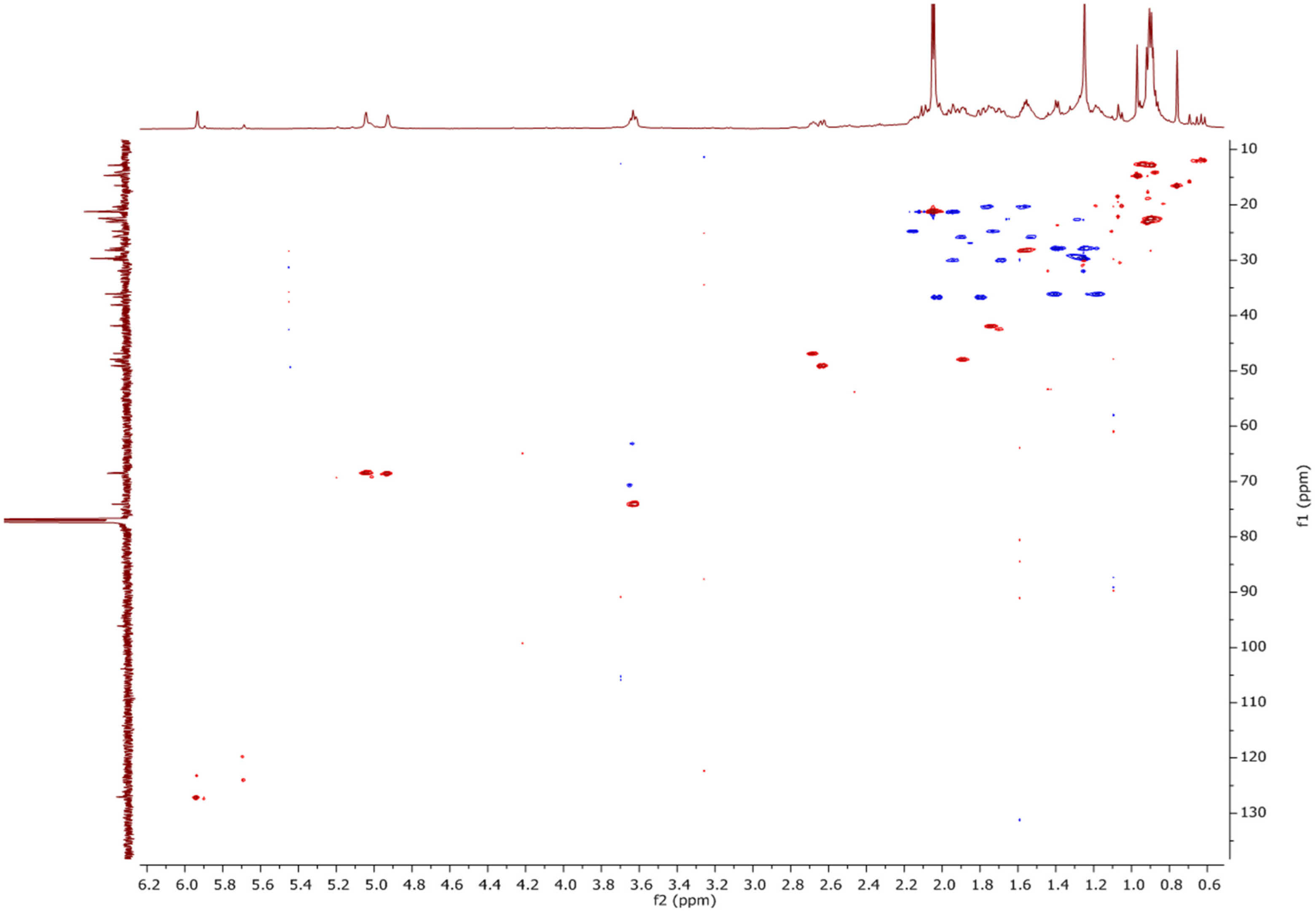

Figure S46. HSQC-DEPT NMR spectrum (600MHz, $CDCl_3$, 303K) of compound **9**

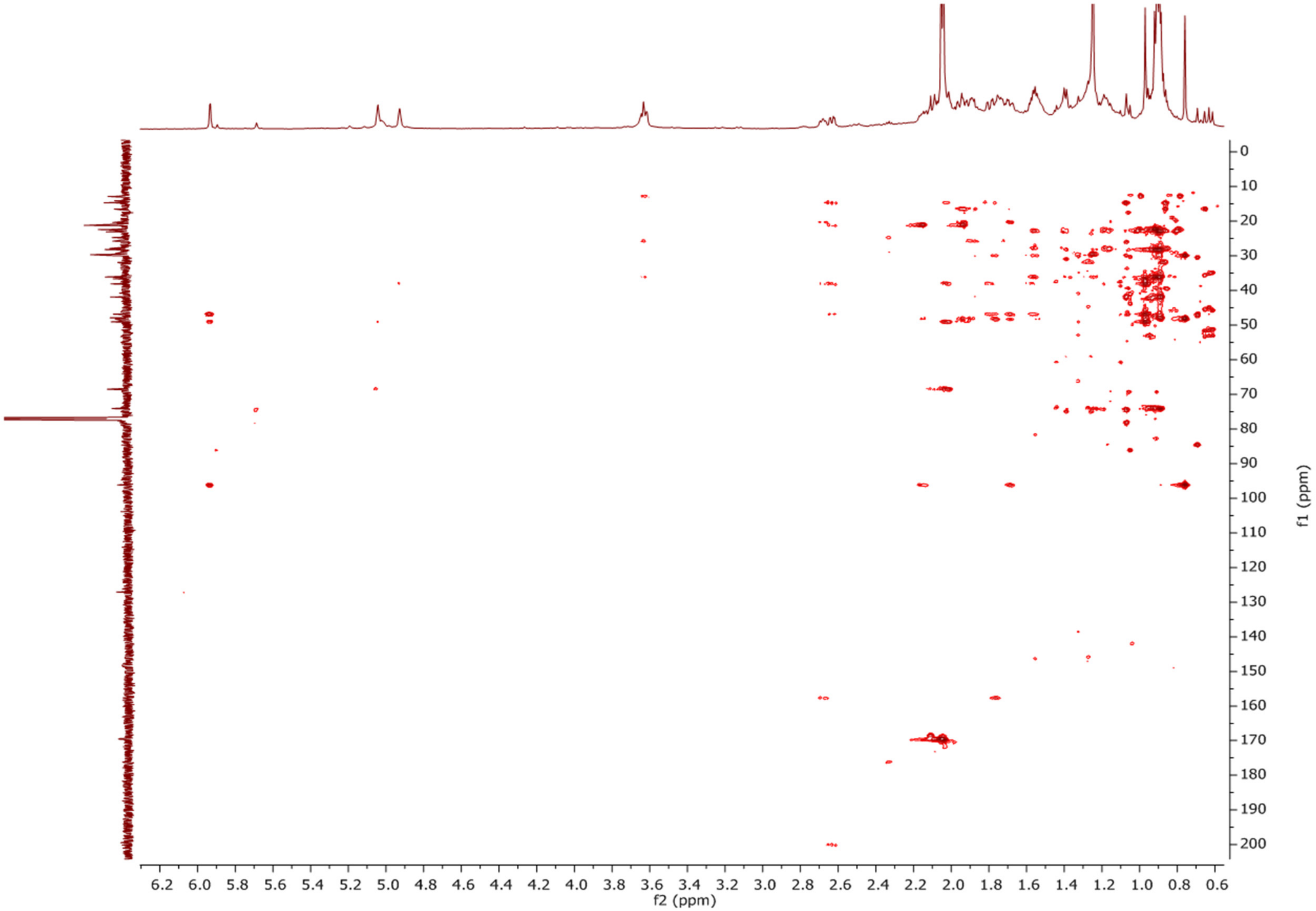

Figure S47. HMBC NMR spectrum (600MHz, $CDCl_3$, 303K) of compound **9**

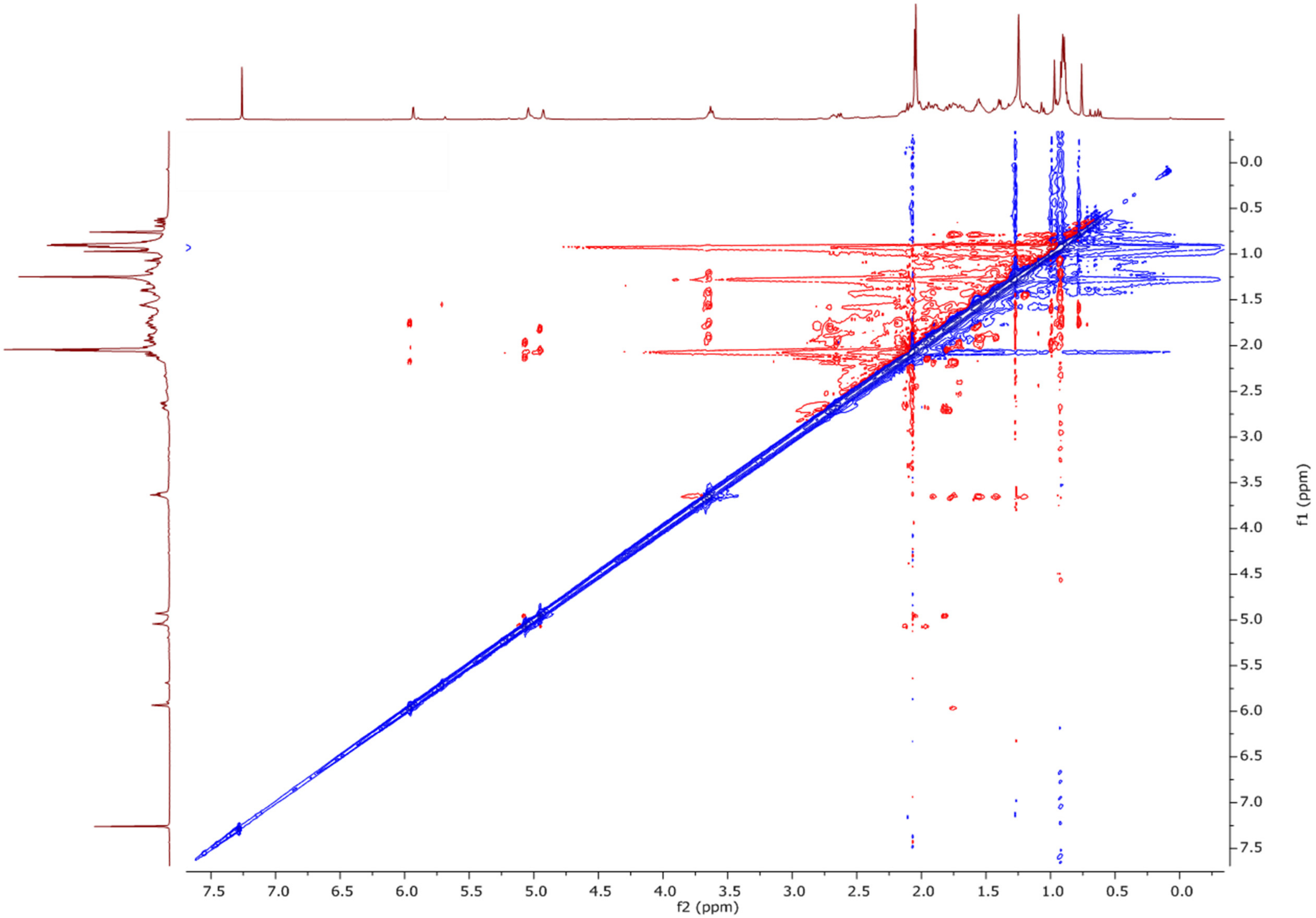

Figure S48. ROESY NMR spectrum (600MHz, $CDCl_3$, 303K) of compound **9**

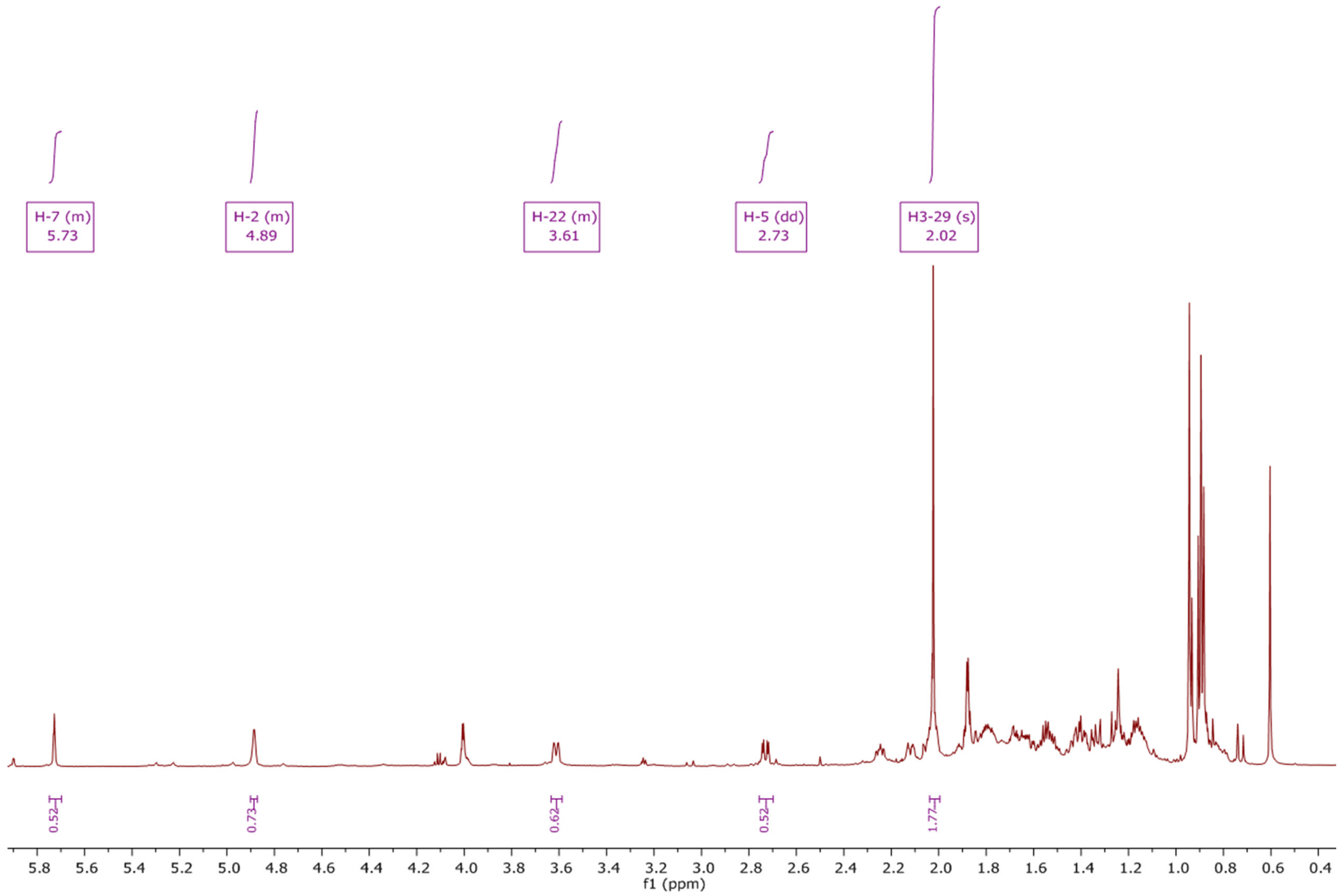

Figure S49. ^{1}H-NMR spectrum (600MHz, $CDCl_3$, 303K) of compound **10**

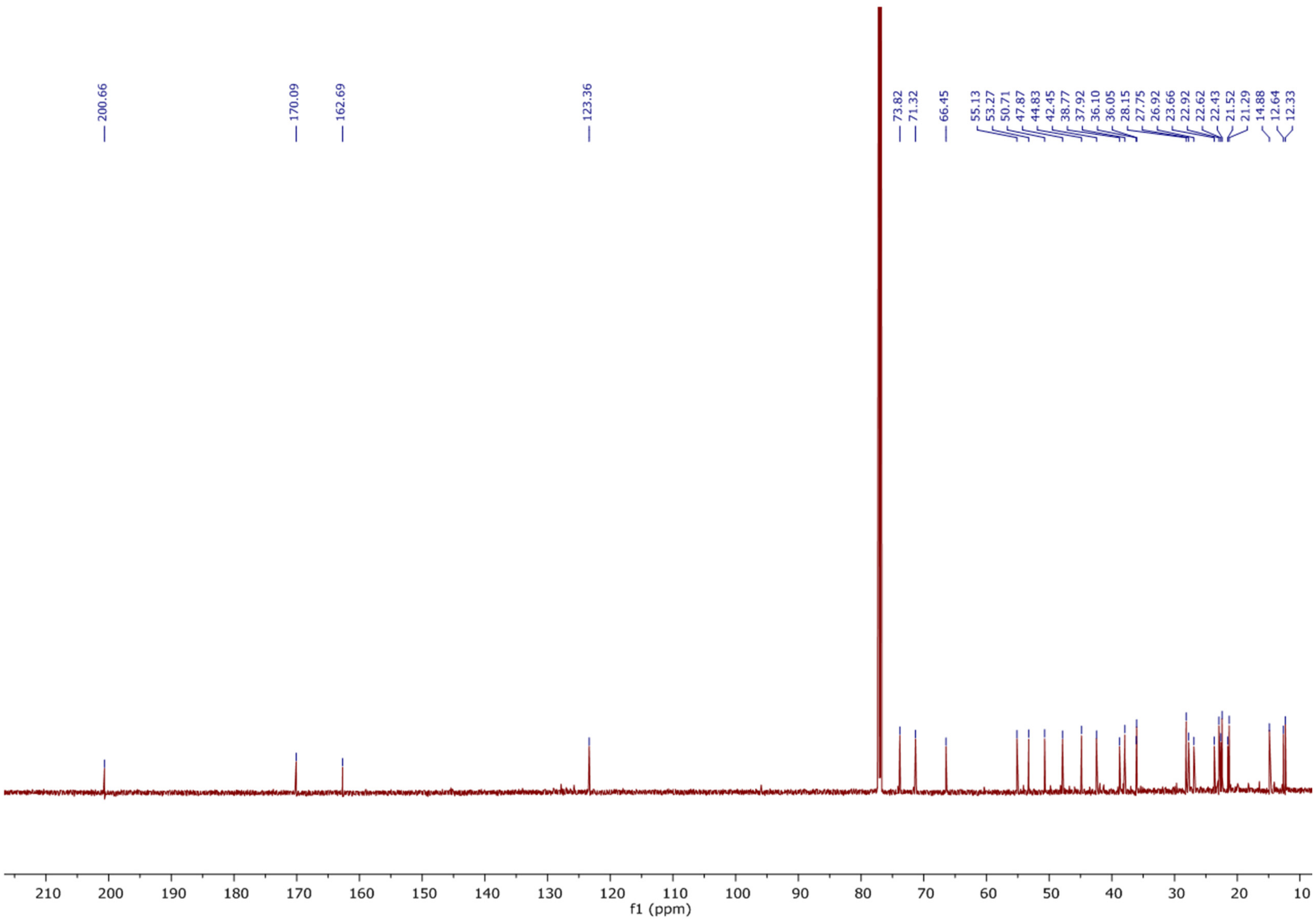

Figure S50. ^{13}C-NMR spectrum (150MHz, $CDCl_3$, 303K) of compound **10**

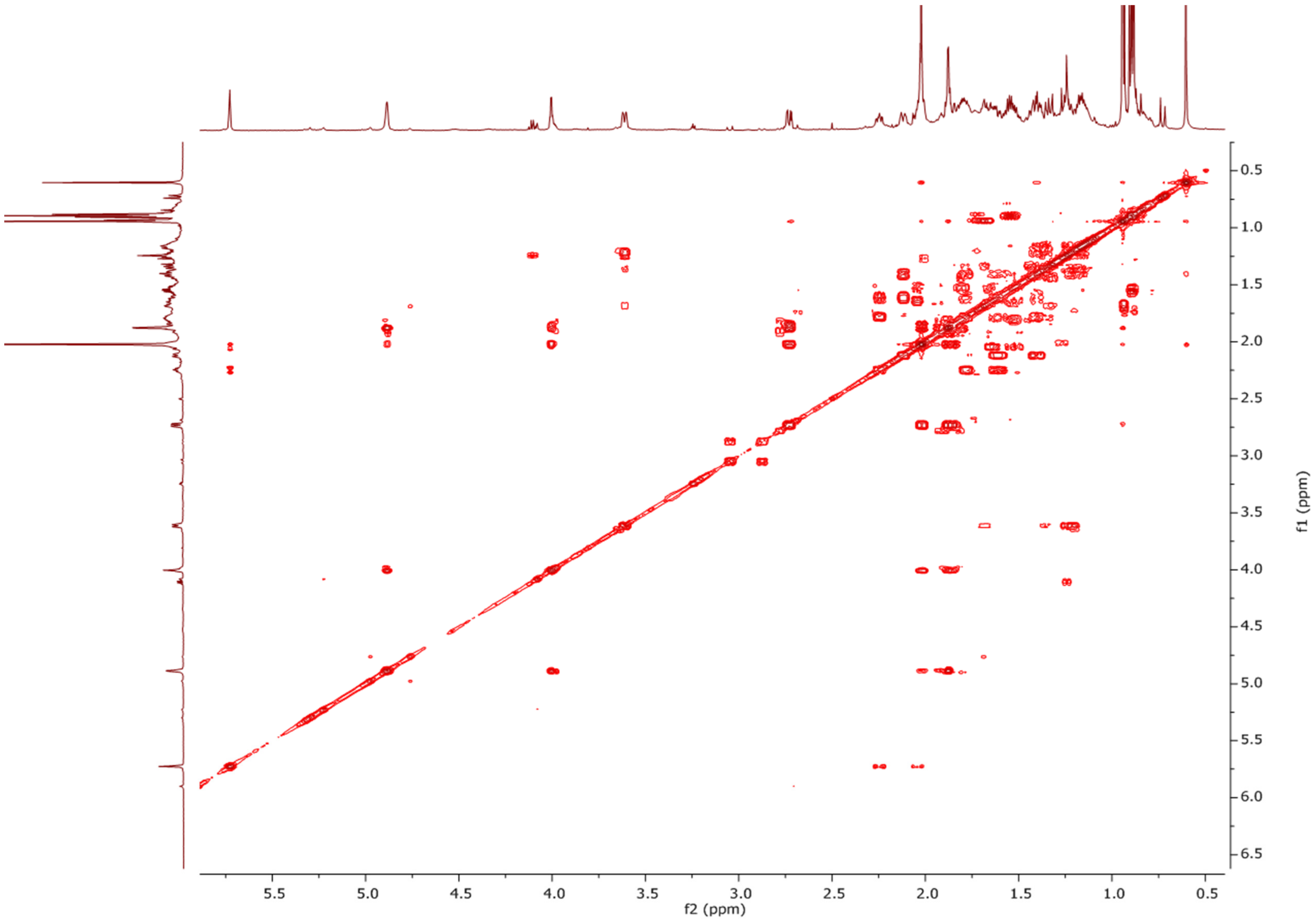

Figure S51. ^{1}H-^{1}H COSY NMR spectrum (600MHz, $CDCl_3$, 303K) of compound **10**

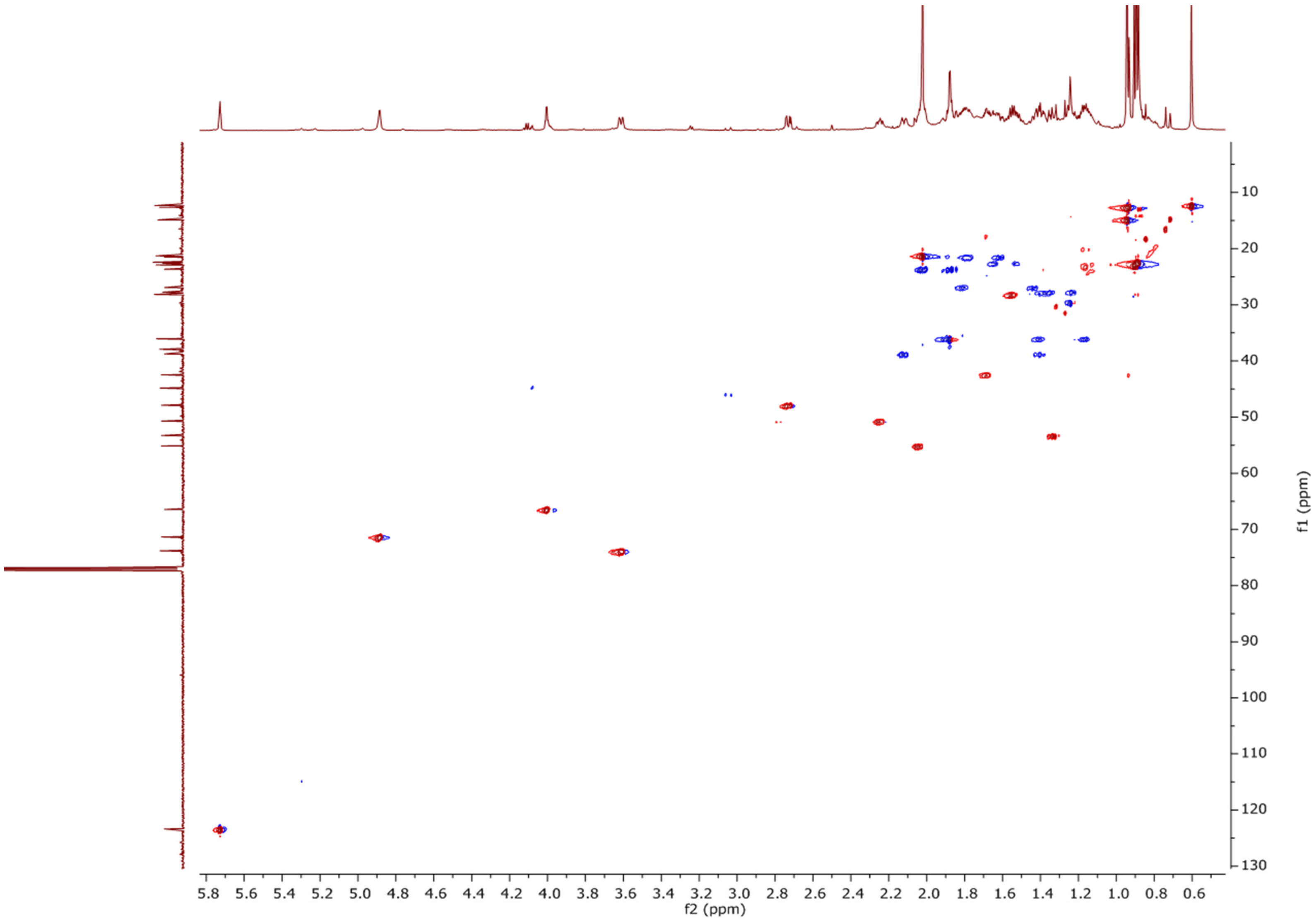

Figure S52. HSQC-DEPT NMR spectrum (600MHz, $CDCl_3$, 303K) of compound **10**

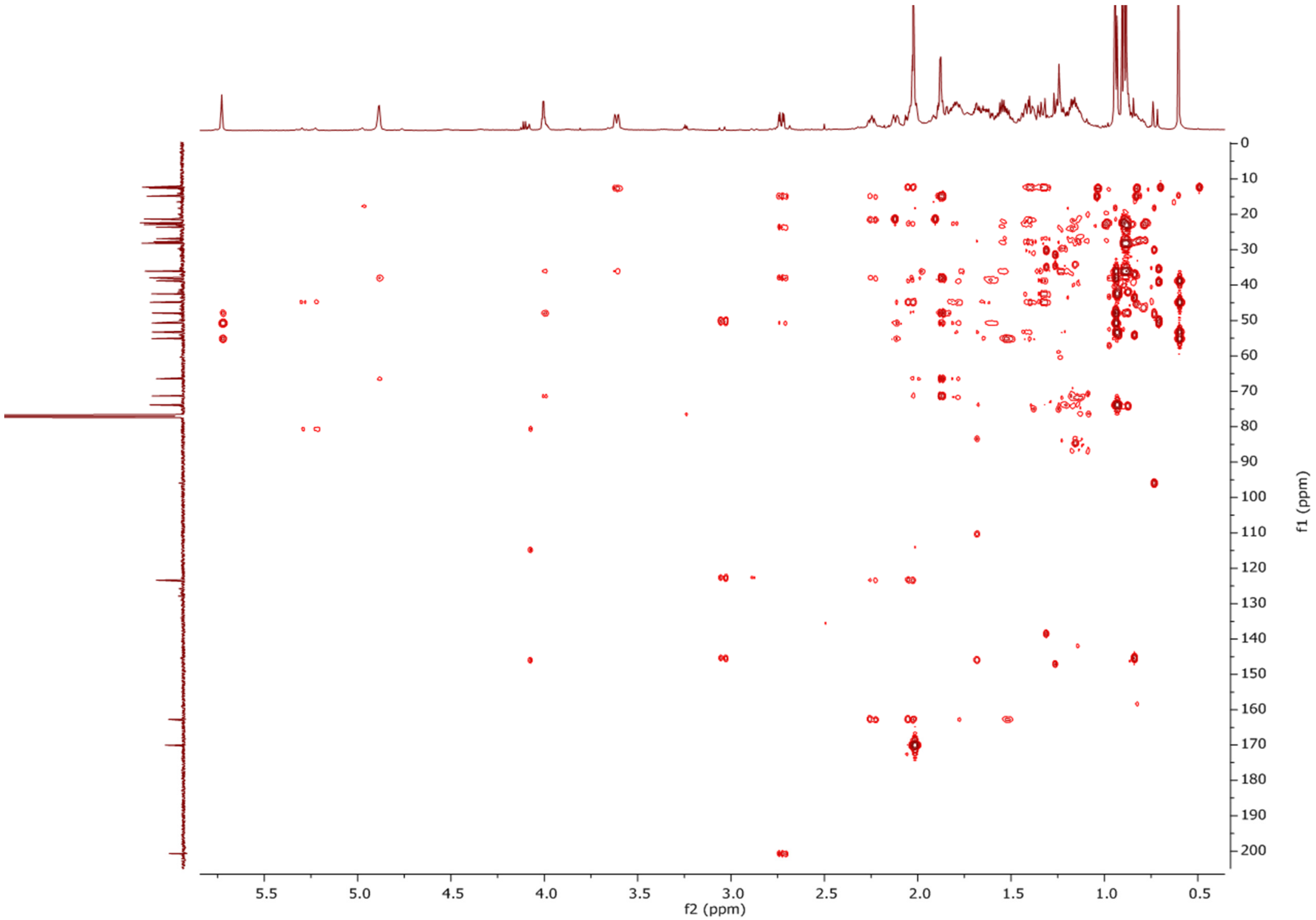

Figure S53. HMBC NMR spectrum (600MHz, $CDCl_3$, 303K) of compound **10**

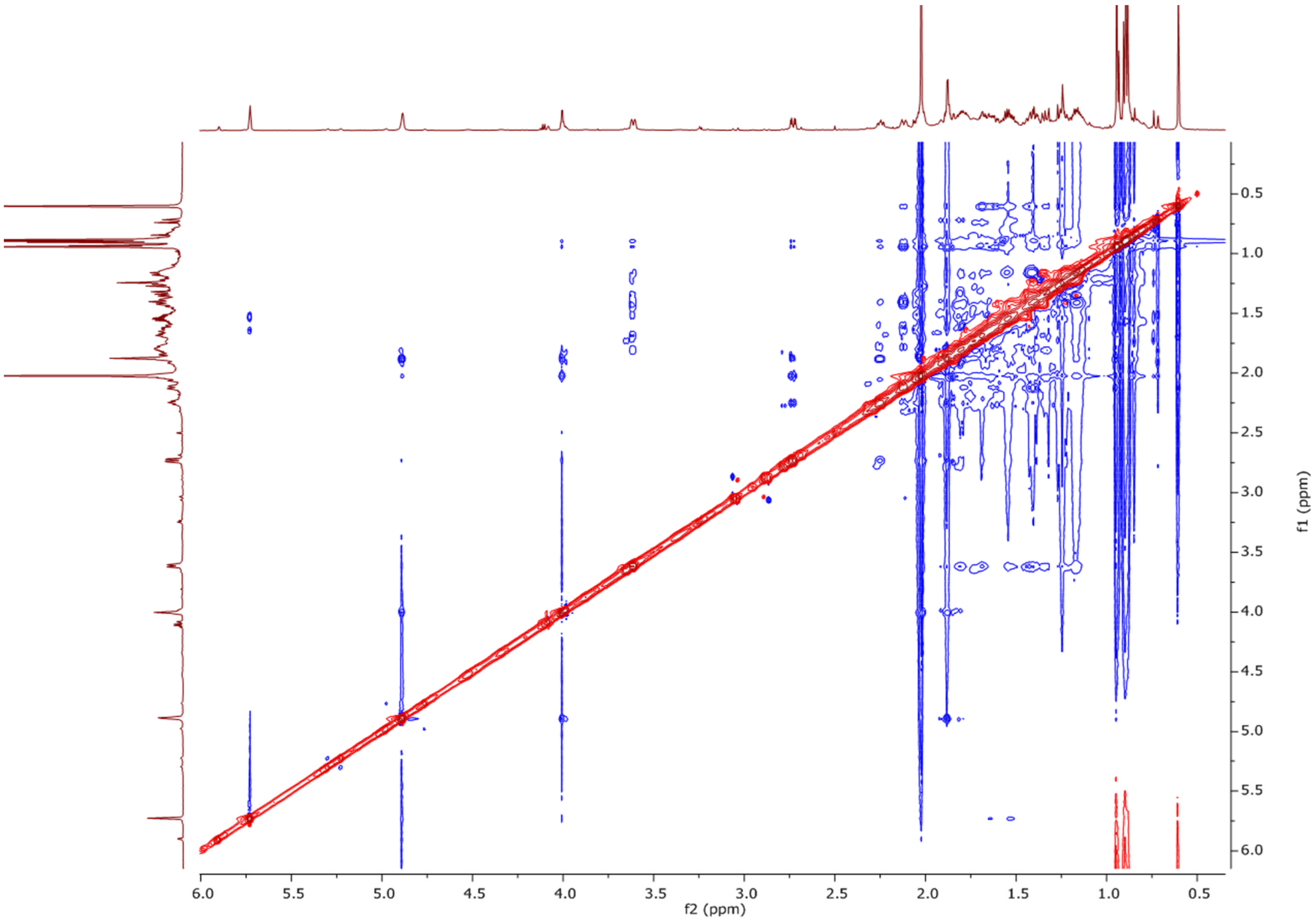

Figure S54. ROESY NMR spectrum (600MHz, $CDCl_3$, 303K) of compound **10**

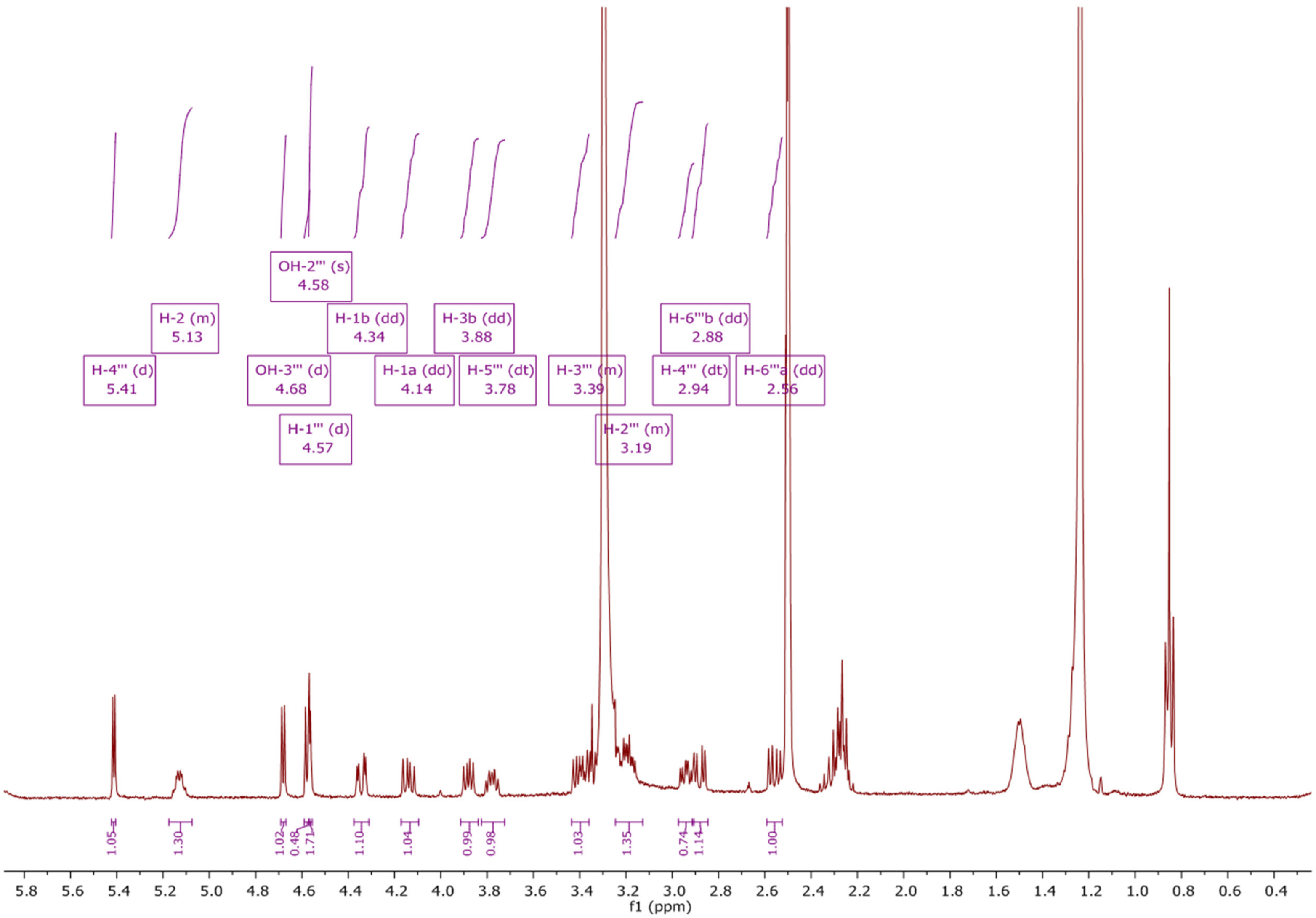

Figure S55. ^{1}H-NMR spectrum (600MHz, DMSO-d$_6$, 303K) of compound **11**

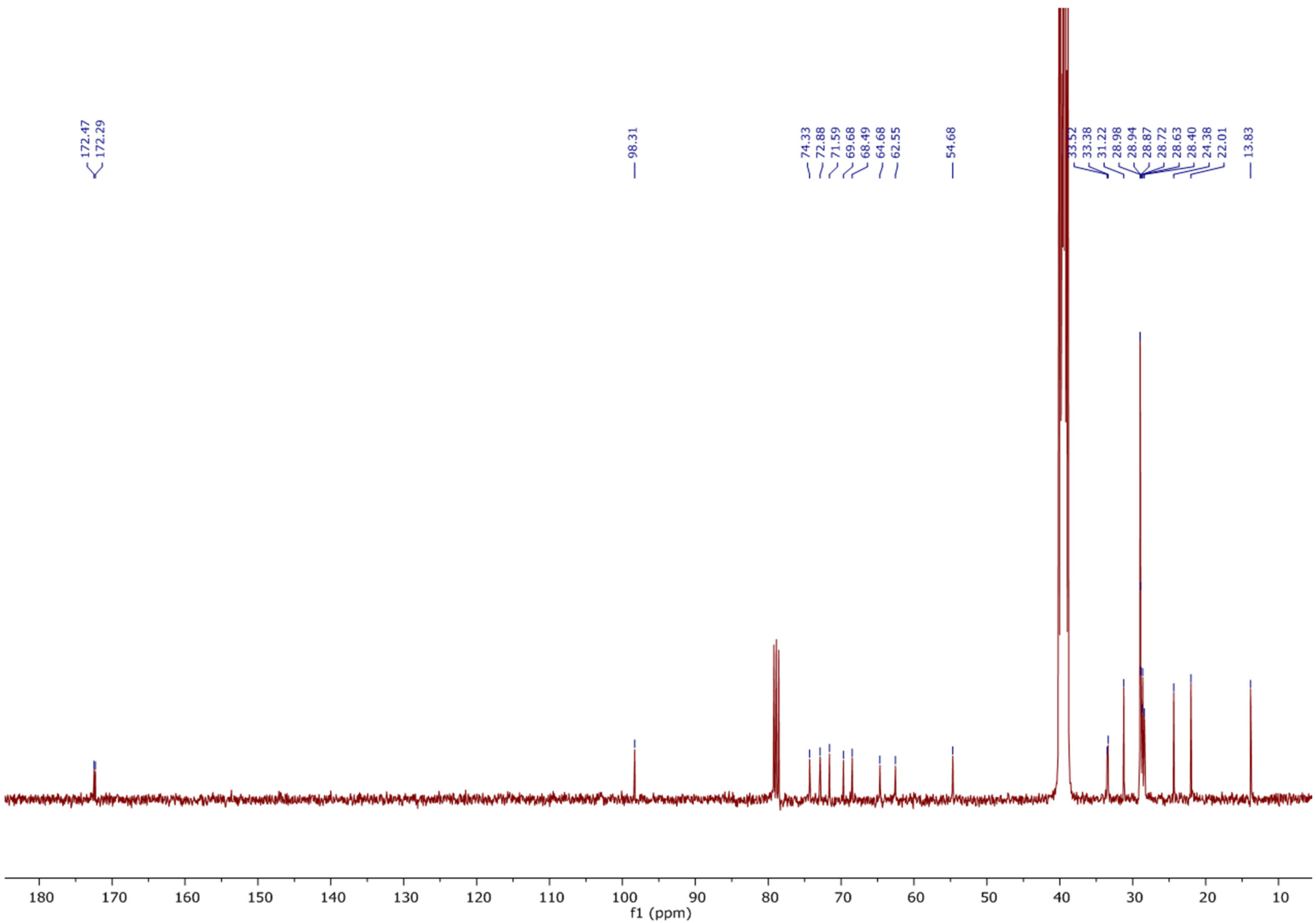

Figure S56. ^{13}C-NMR spectrum (150MHz, DMSO-d6, 303K) of compound **11**

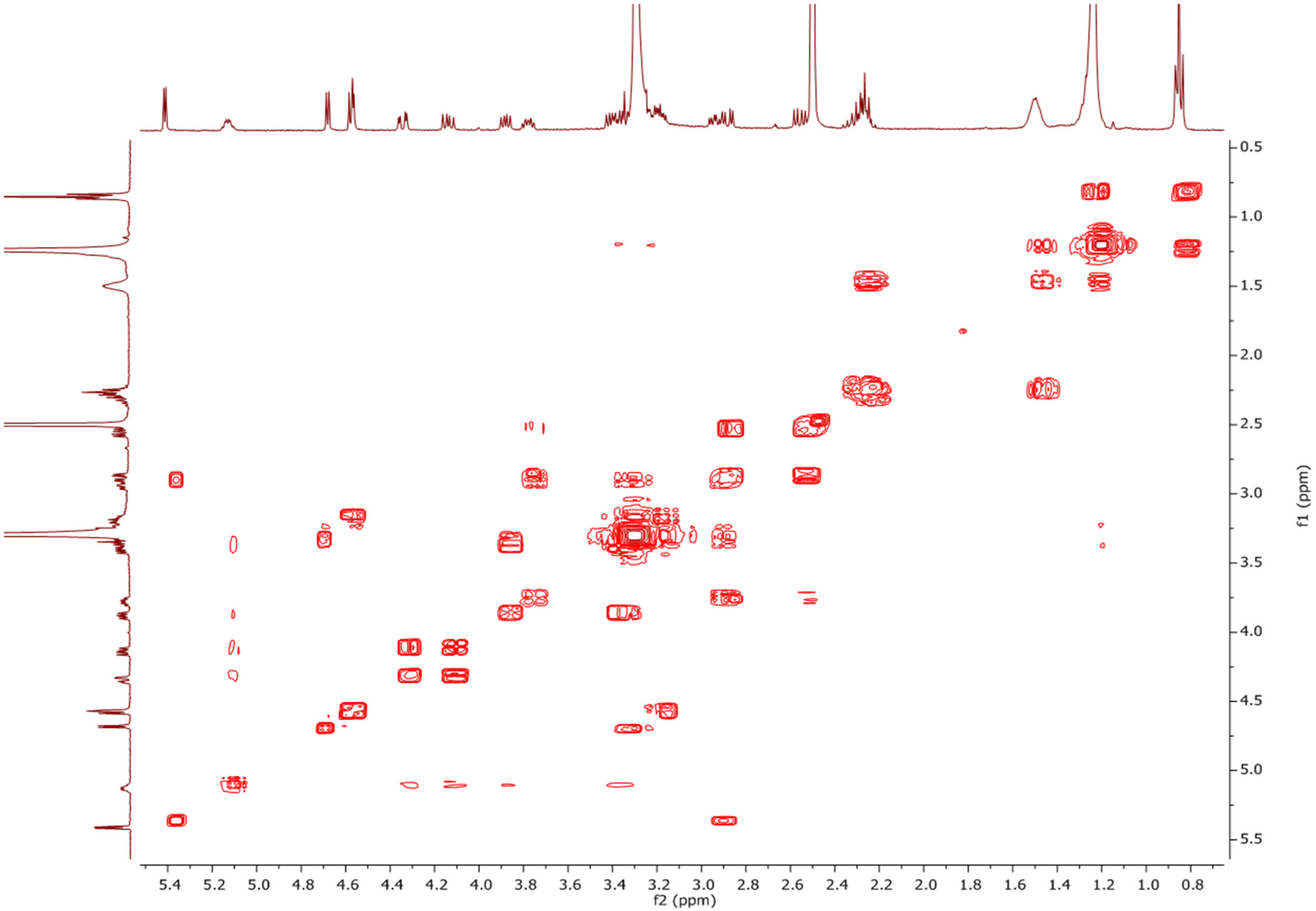

Figure S57. ^1H-^1H COSY NMR spectrum (600MHz, DMSO-d_6, 303K) of compound **11**

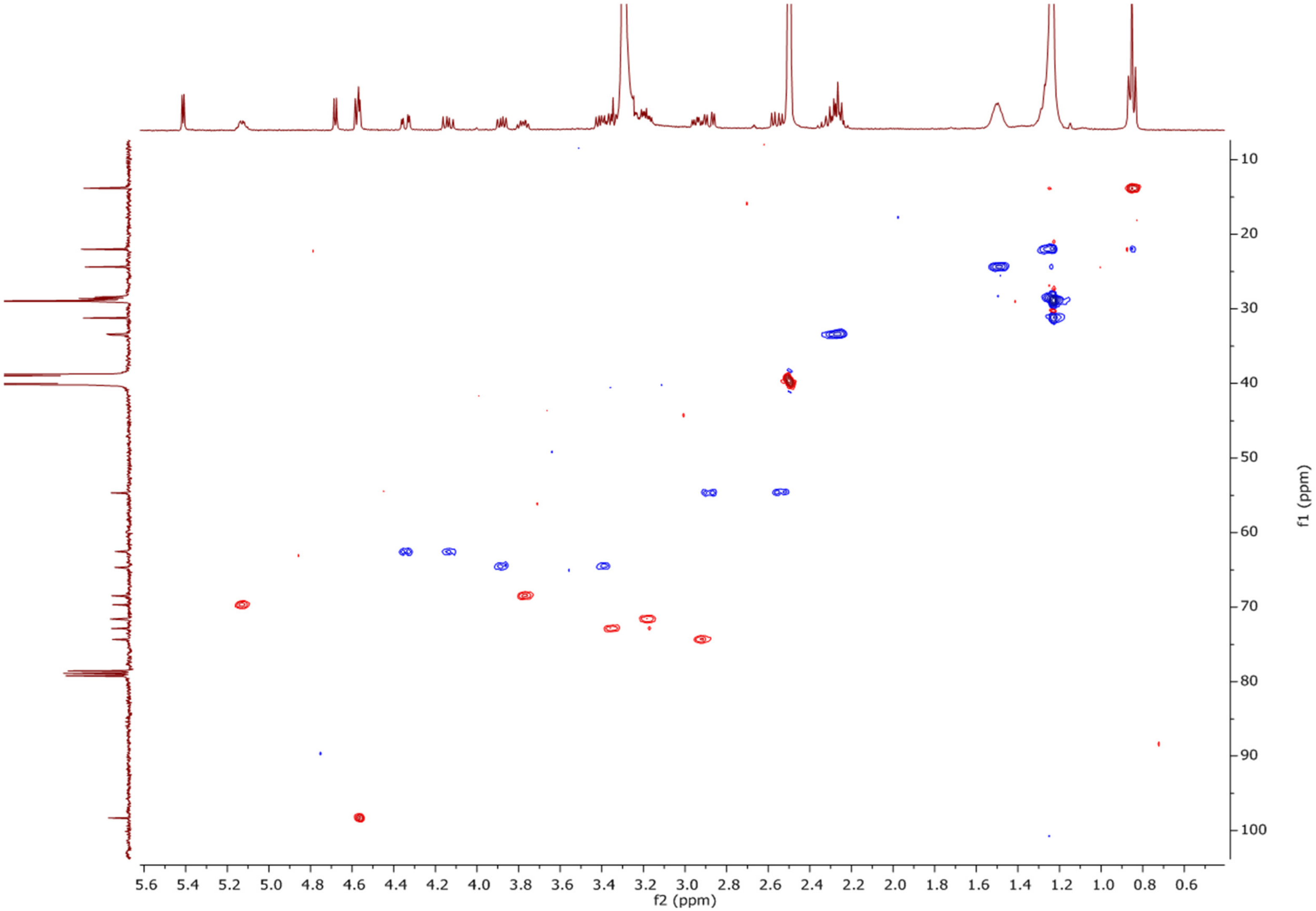

Figure S58. HSQC-DEPT NMR spectrum (600MHz, DMSO-d6, 303K) of compound **11**

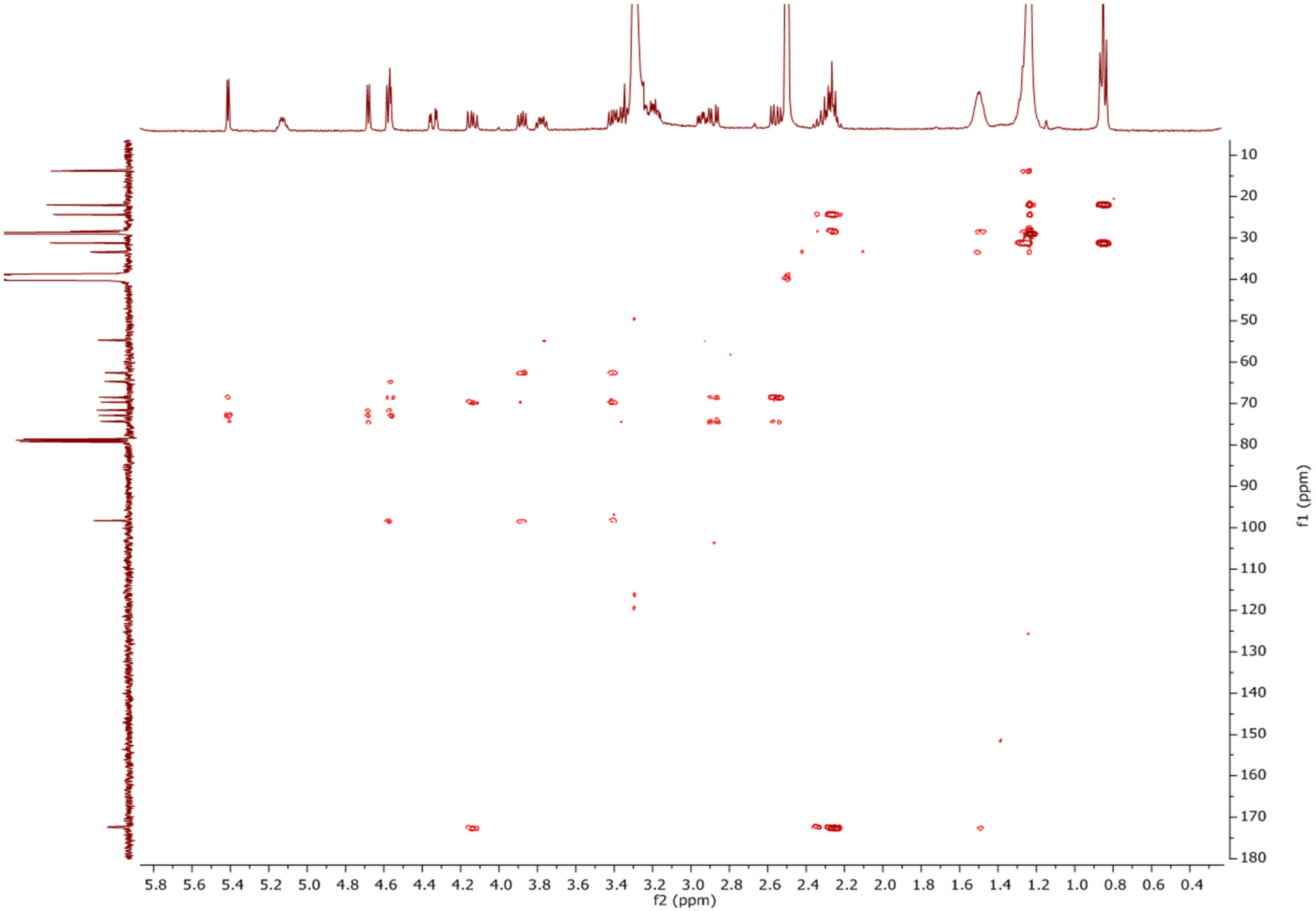

Figure S59. HMBC NMR spectrum (600MHz, DMSO-d_6, 303K) of compound **11**

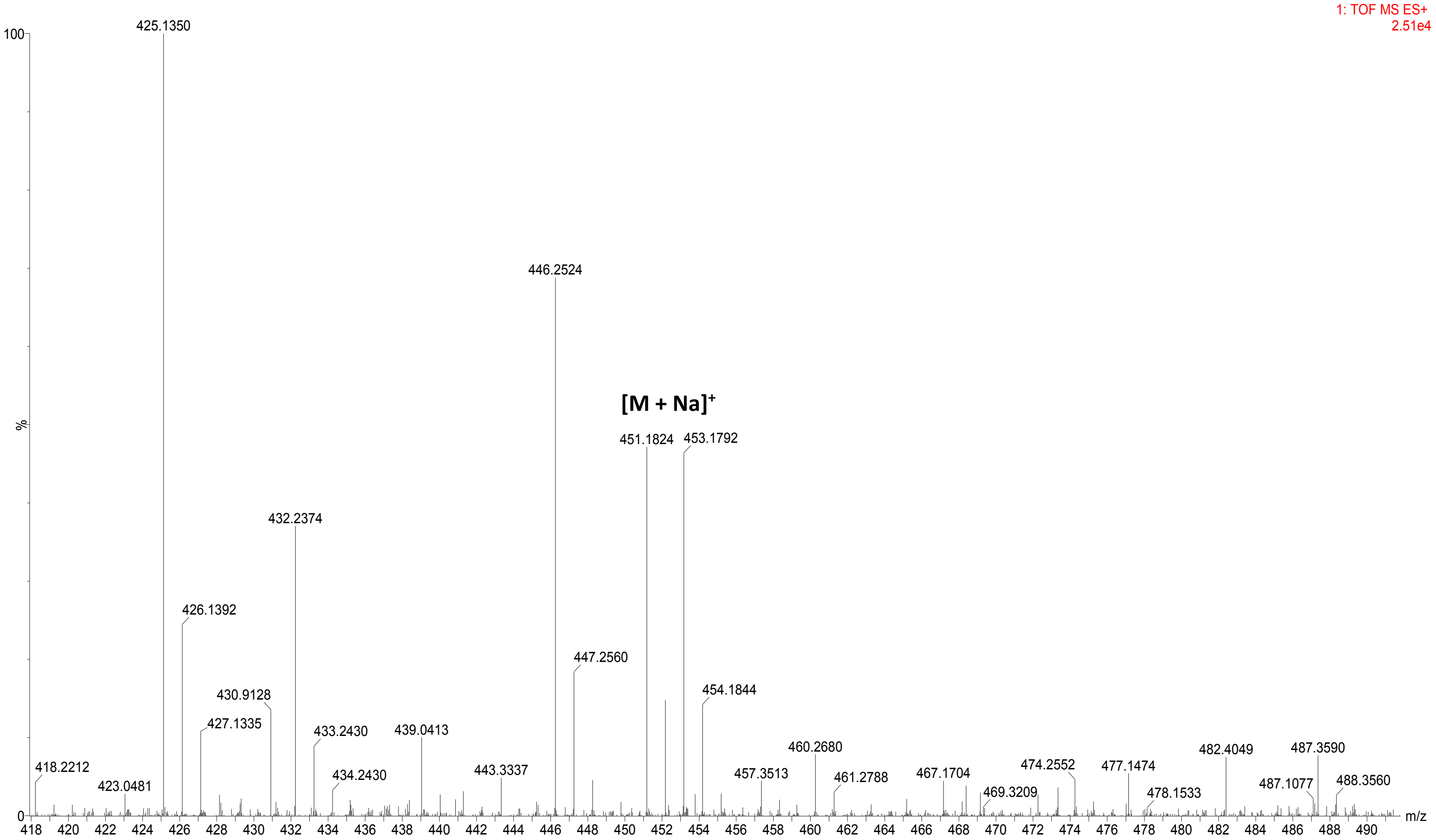

Figure S60. HR-ESI-MS spectrum of compound **1**

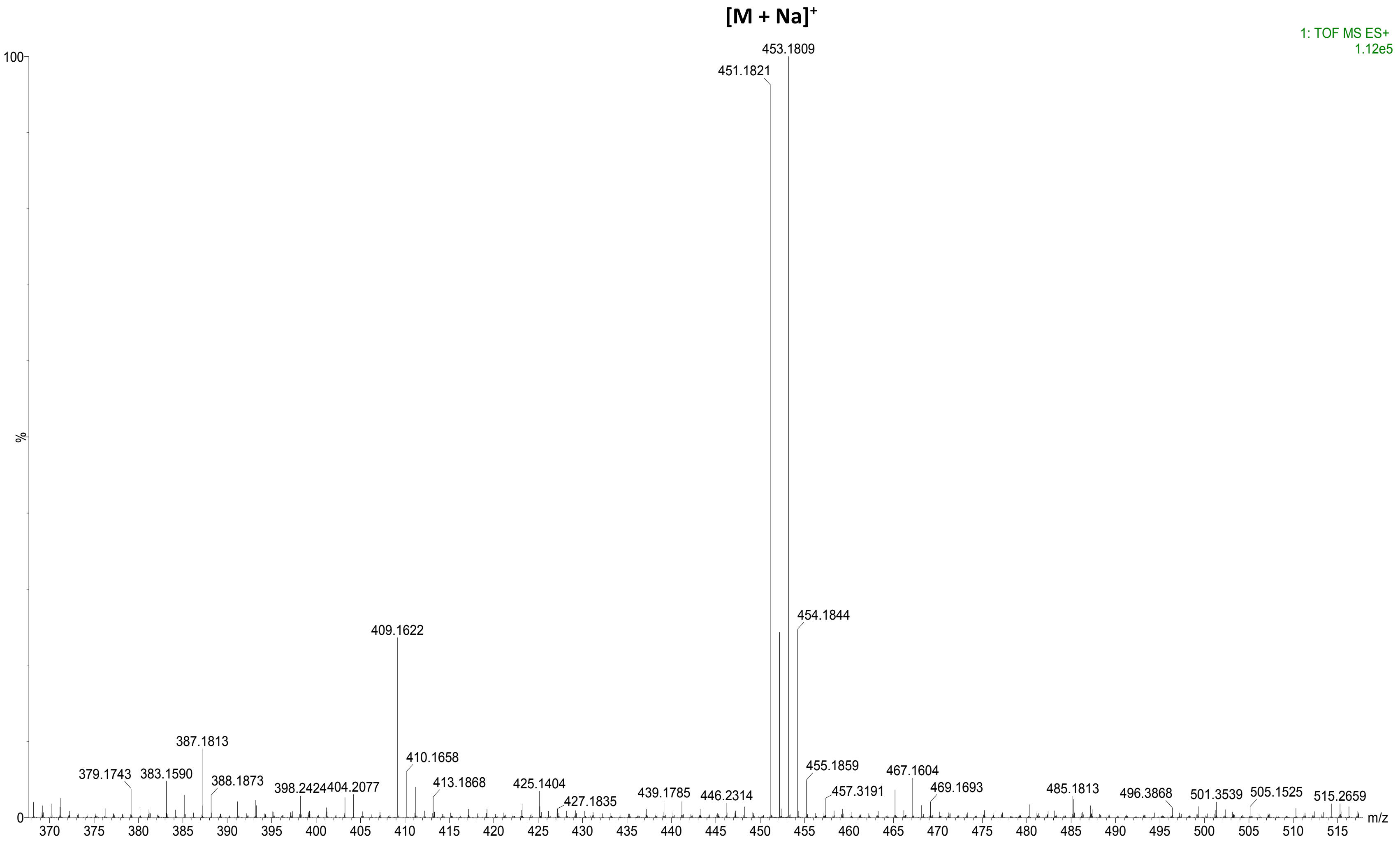

Figure S61. HR-ESI-MS spectrum of compound **2**

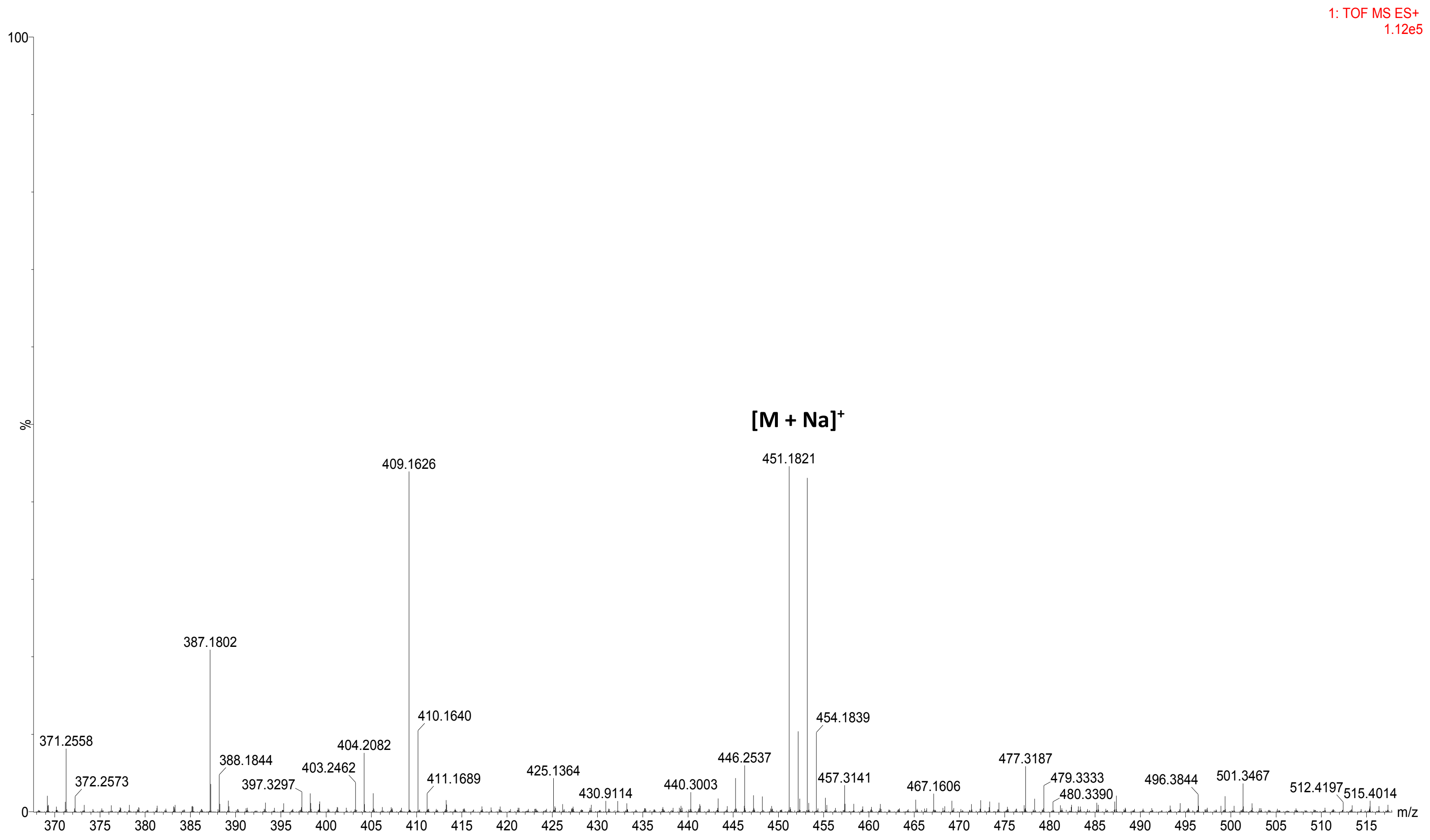

Figure S62. HR-ESI-MS spectrum of compound **3**

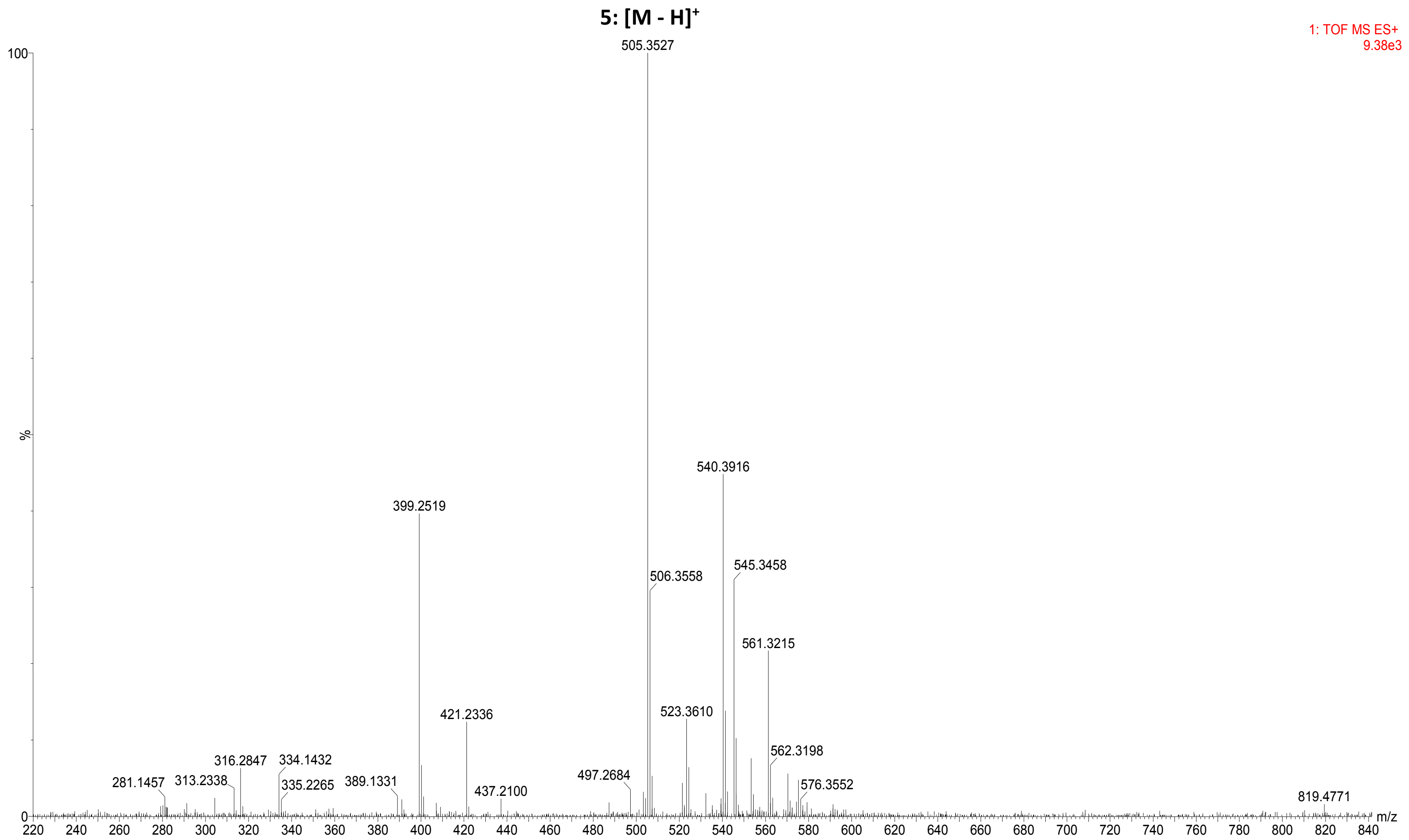

Figure S63. HR-ESI-MS spectrum of compound **5**

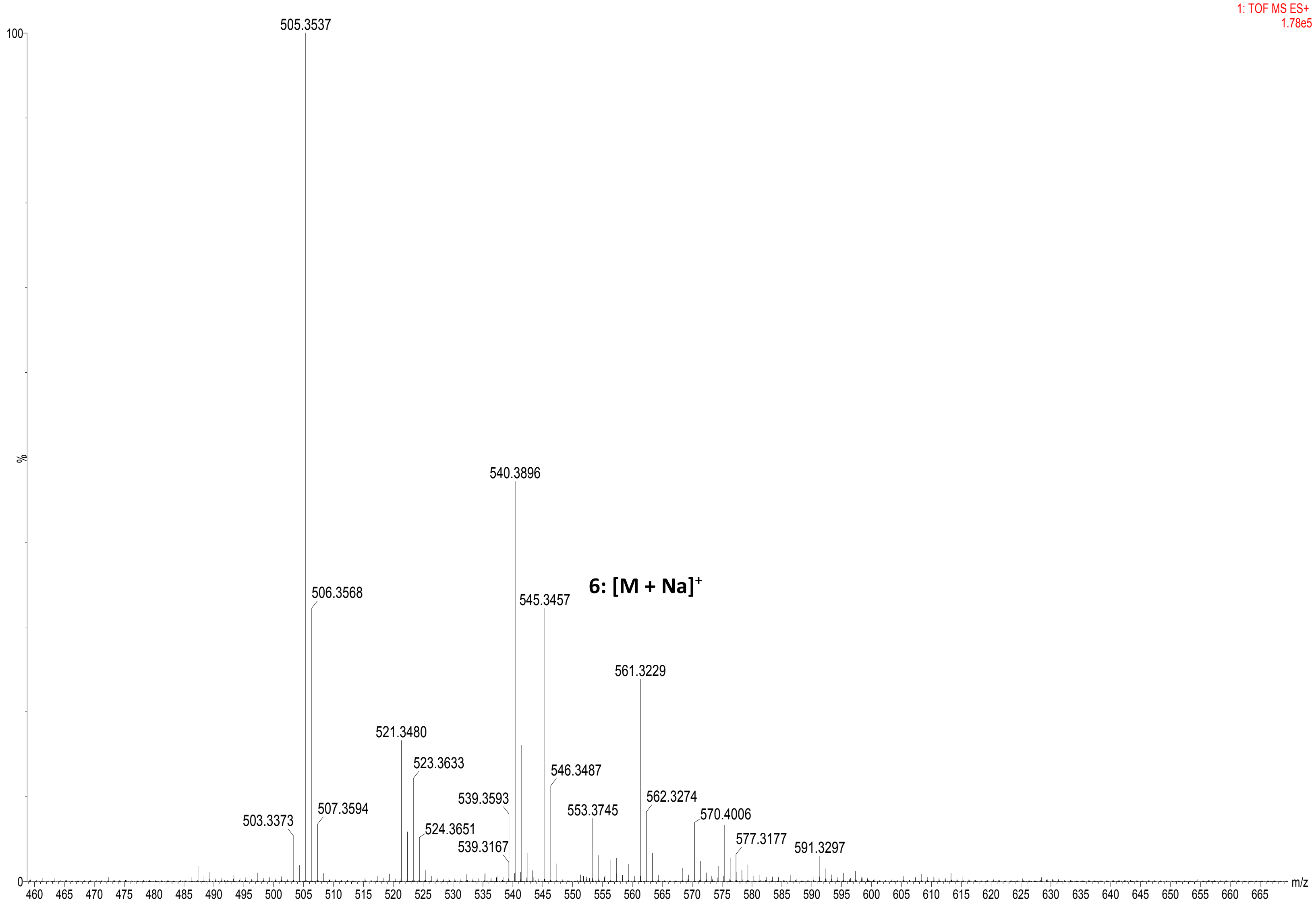

Figure S64. HR-ESI-MS spectrum of compound **6**

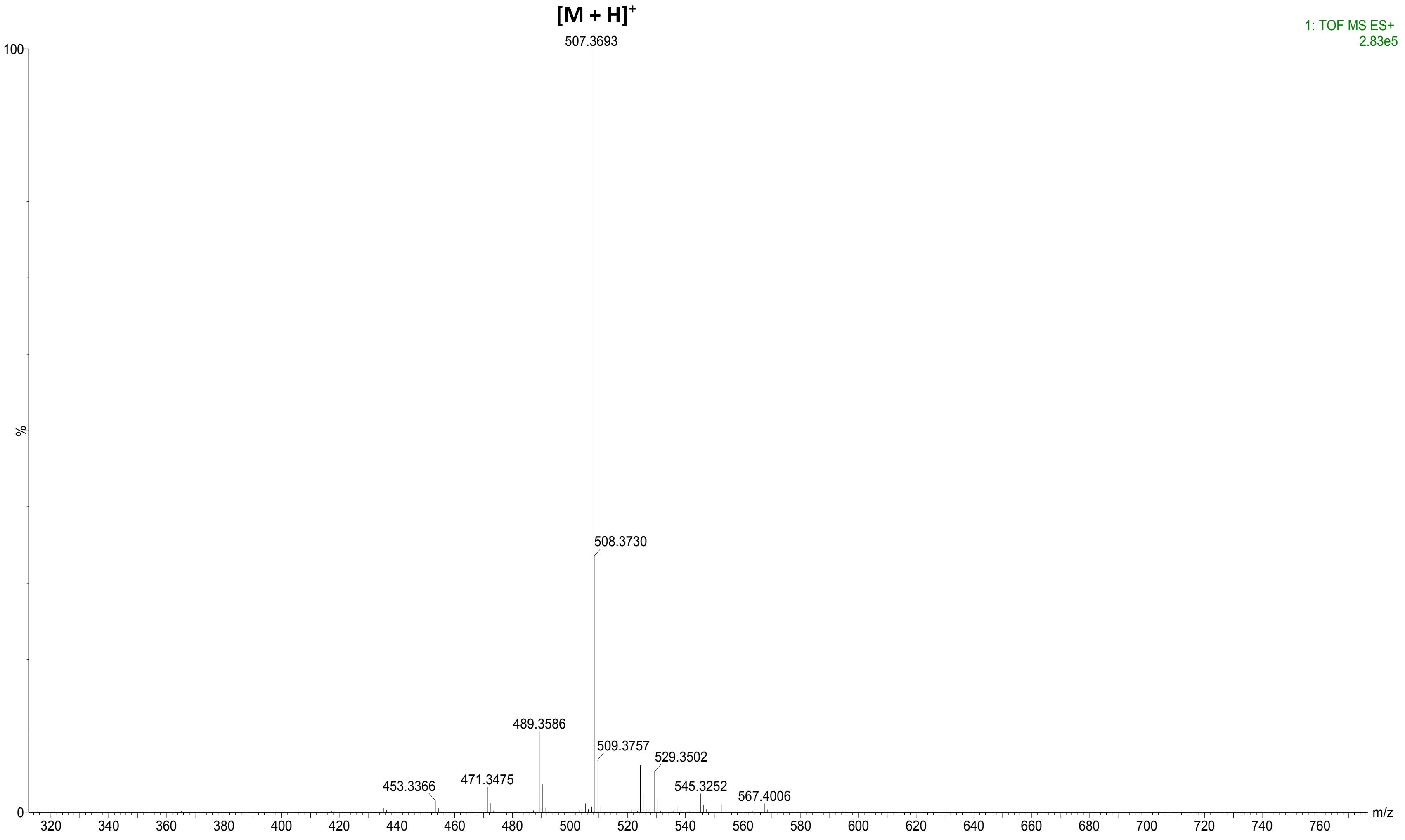

Figure S65. HR-ESI-MS spectrum of compound **7**

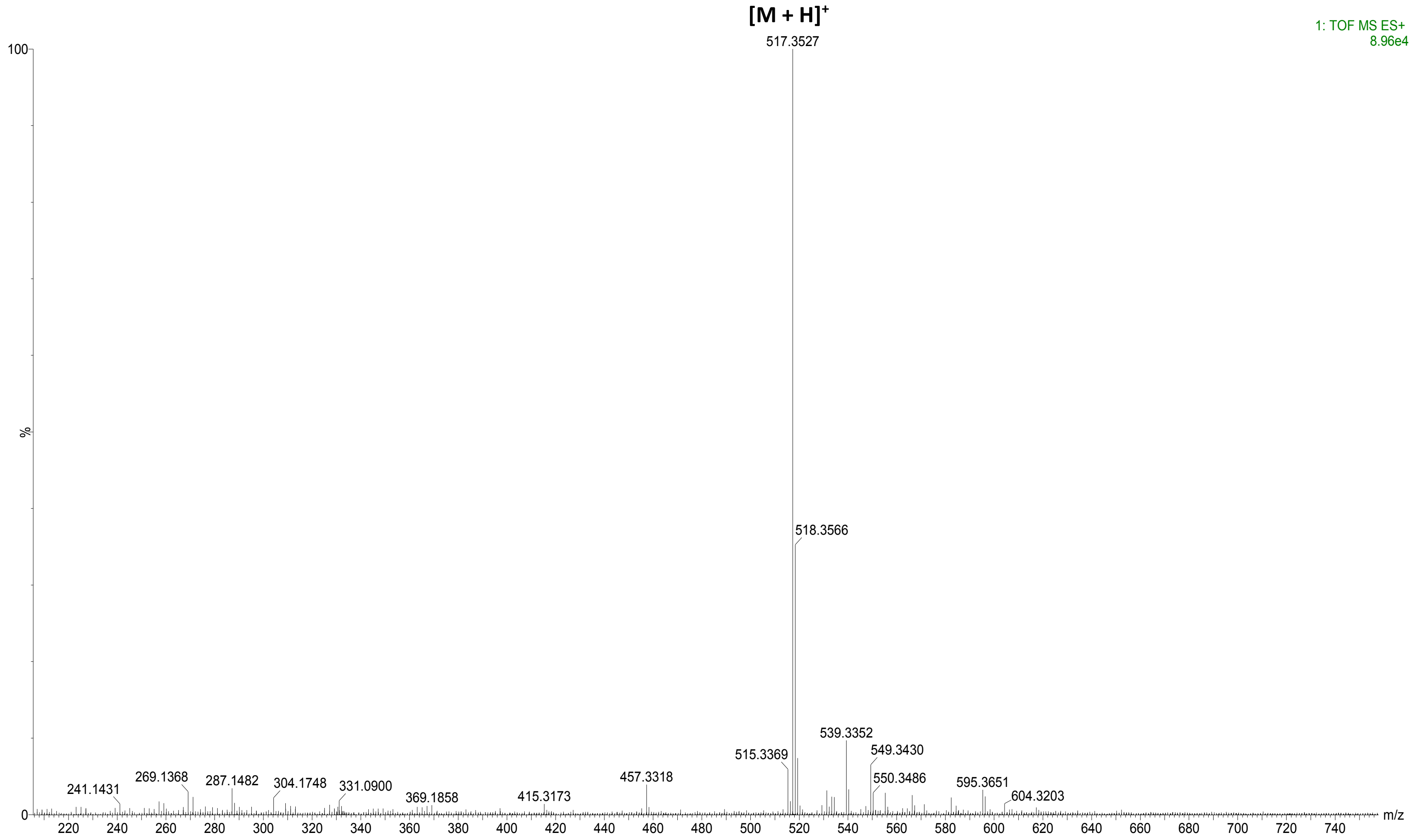

Figure S66. HR-ESI-MS spectrum of compound **8**

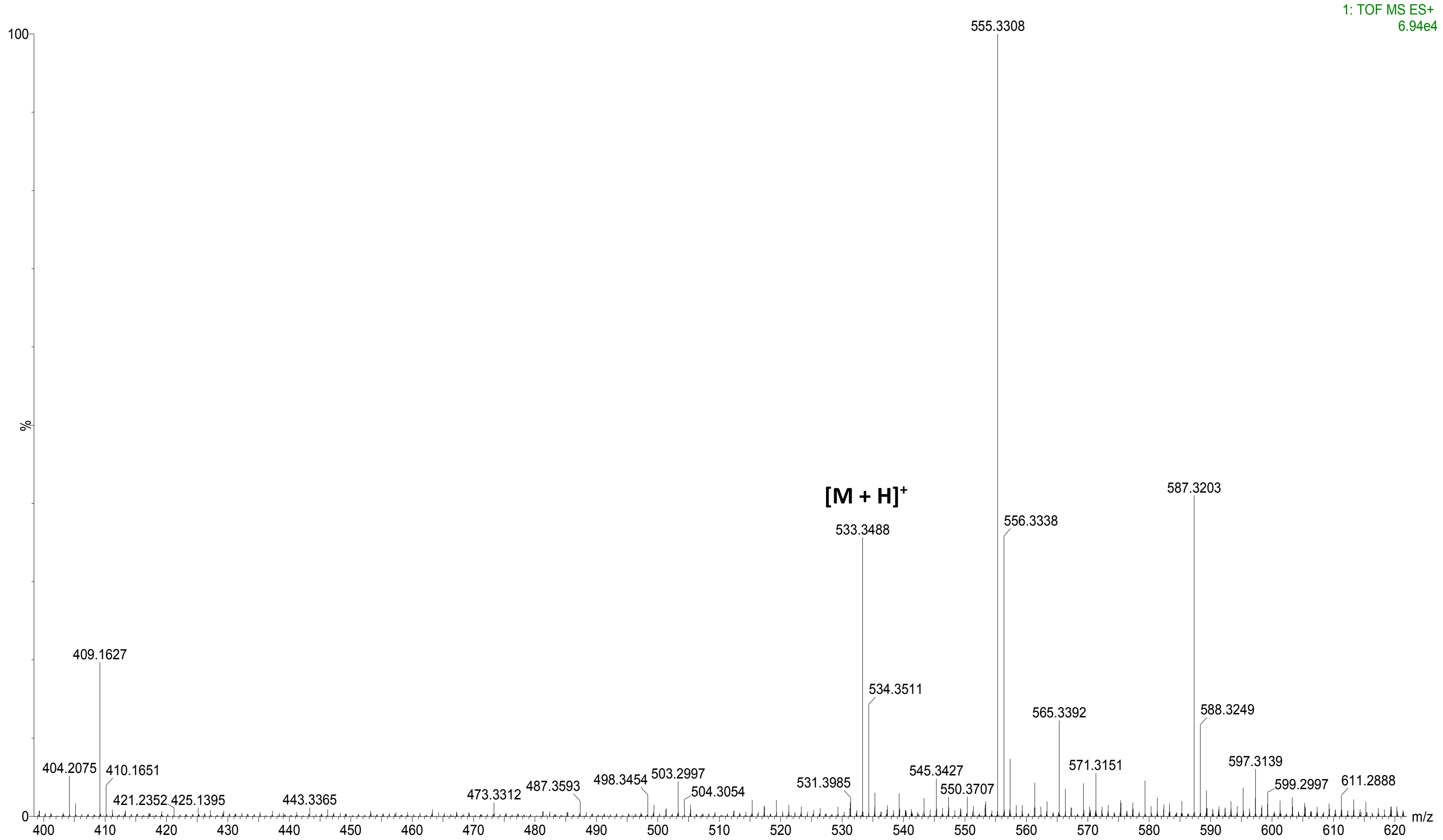

Figure S67. HR-ESI-MS spectrum of compound **9**

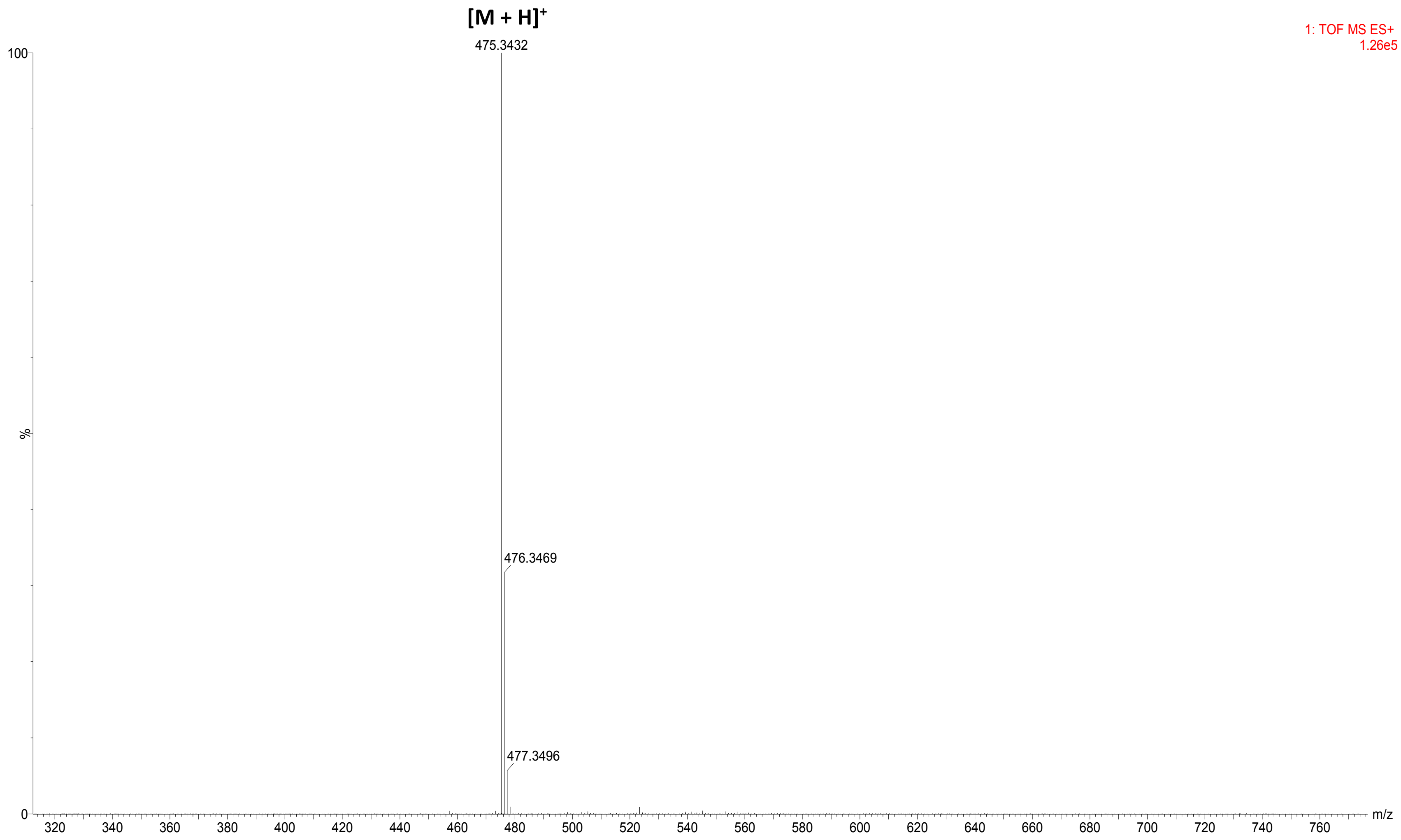

Figure S68. HR-ESI-MS spectrum of compound **10**

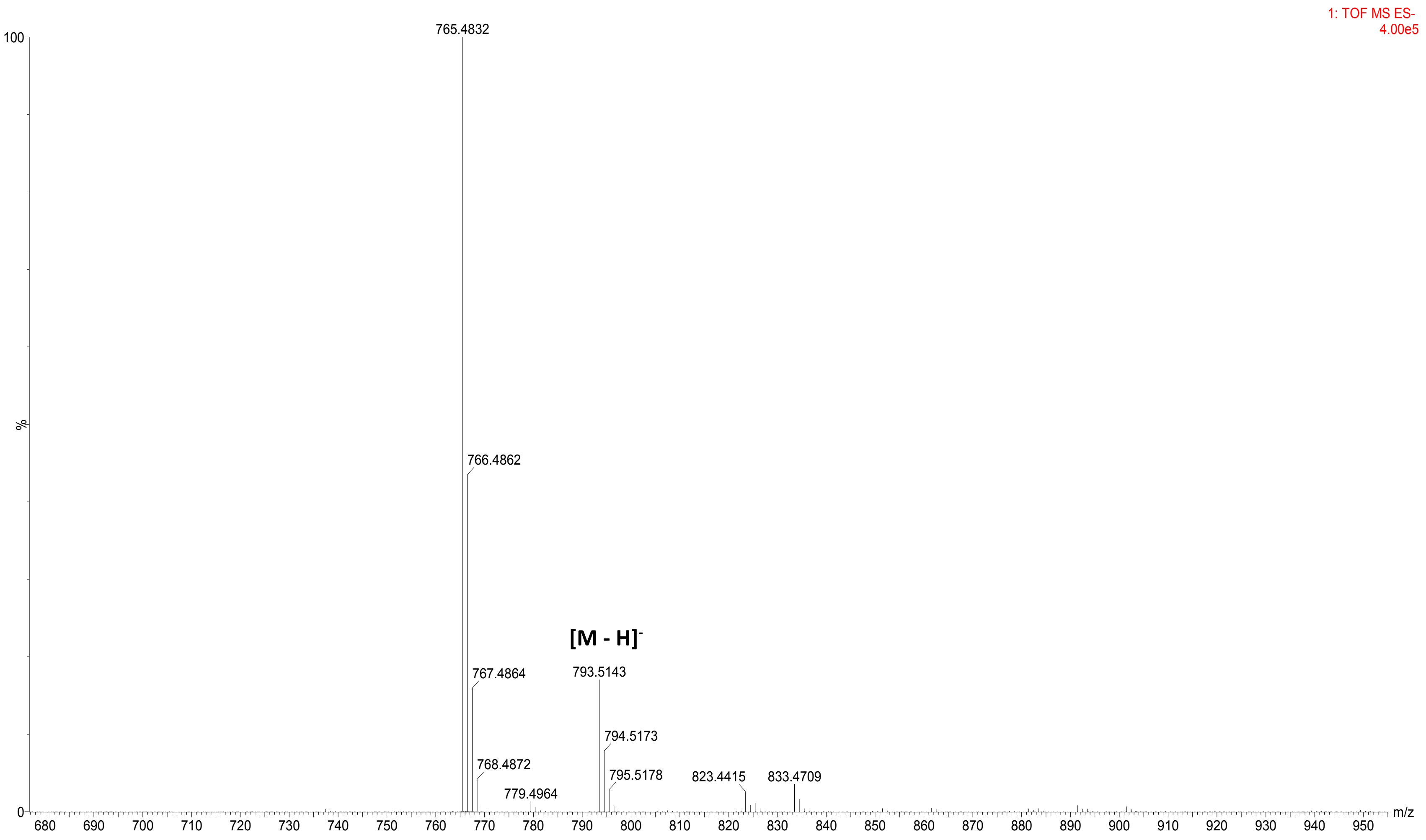

Figure S69. HR-ESI-MS spectrum of compound **11**

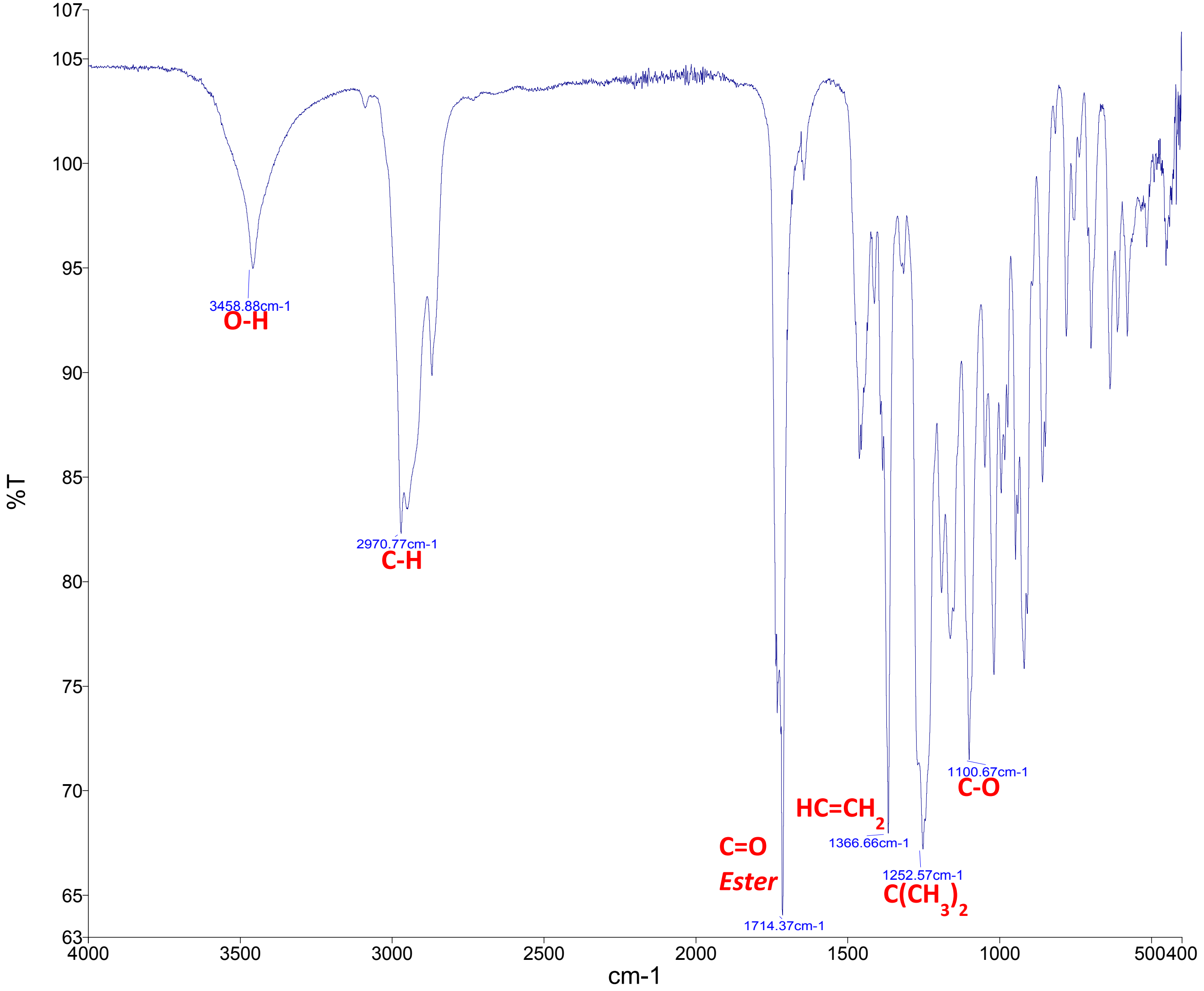

Figure S70. IR spectrum of compound **1**

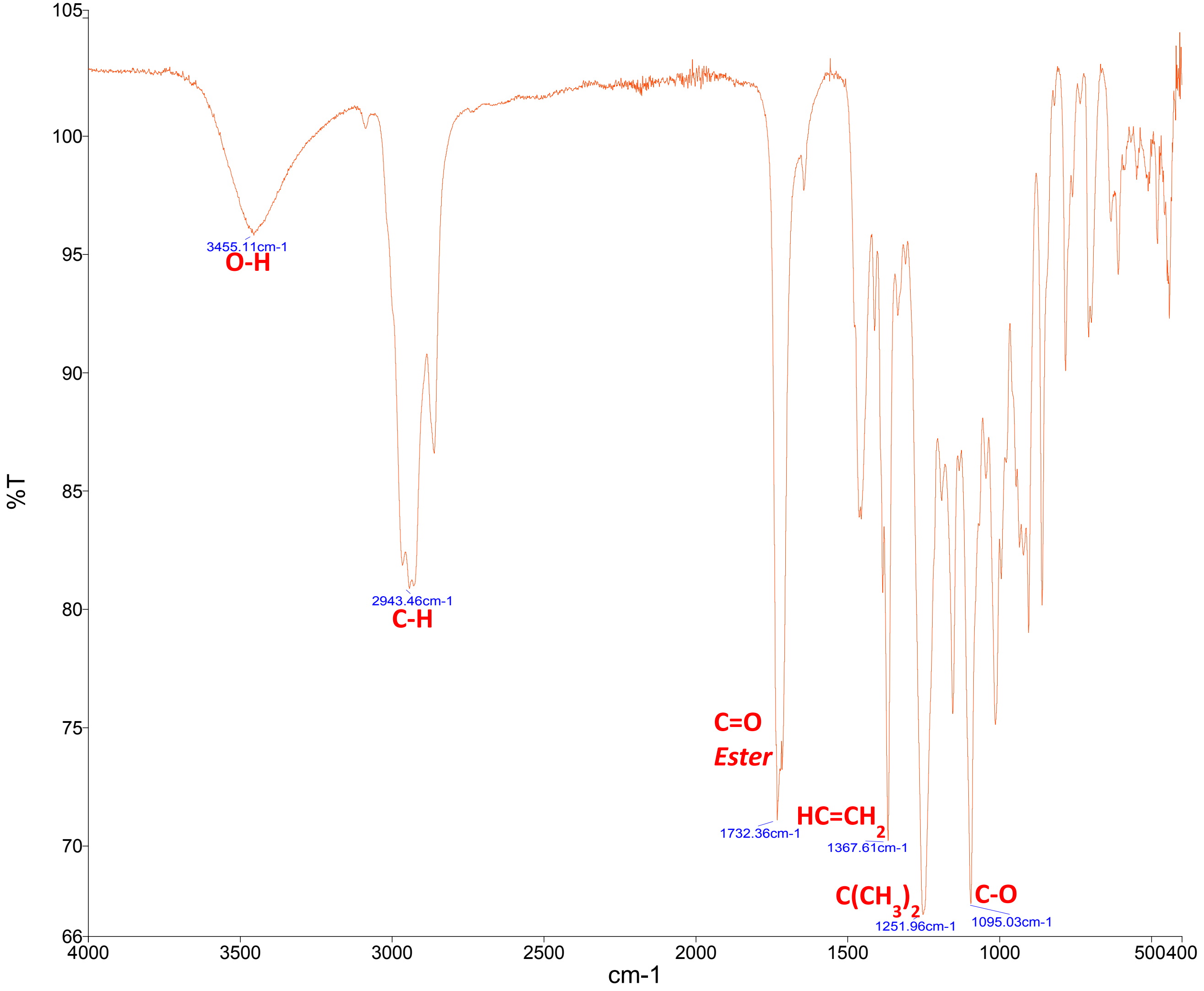

Figure S71. IR spectrum of compound **2**

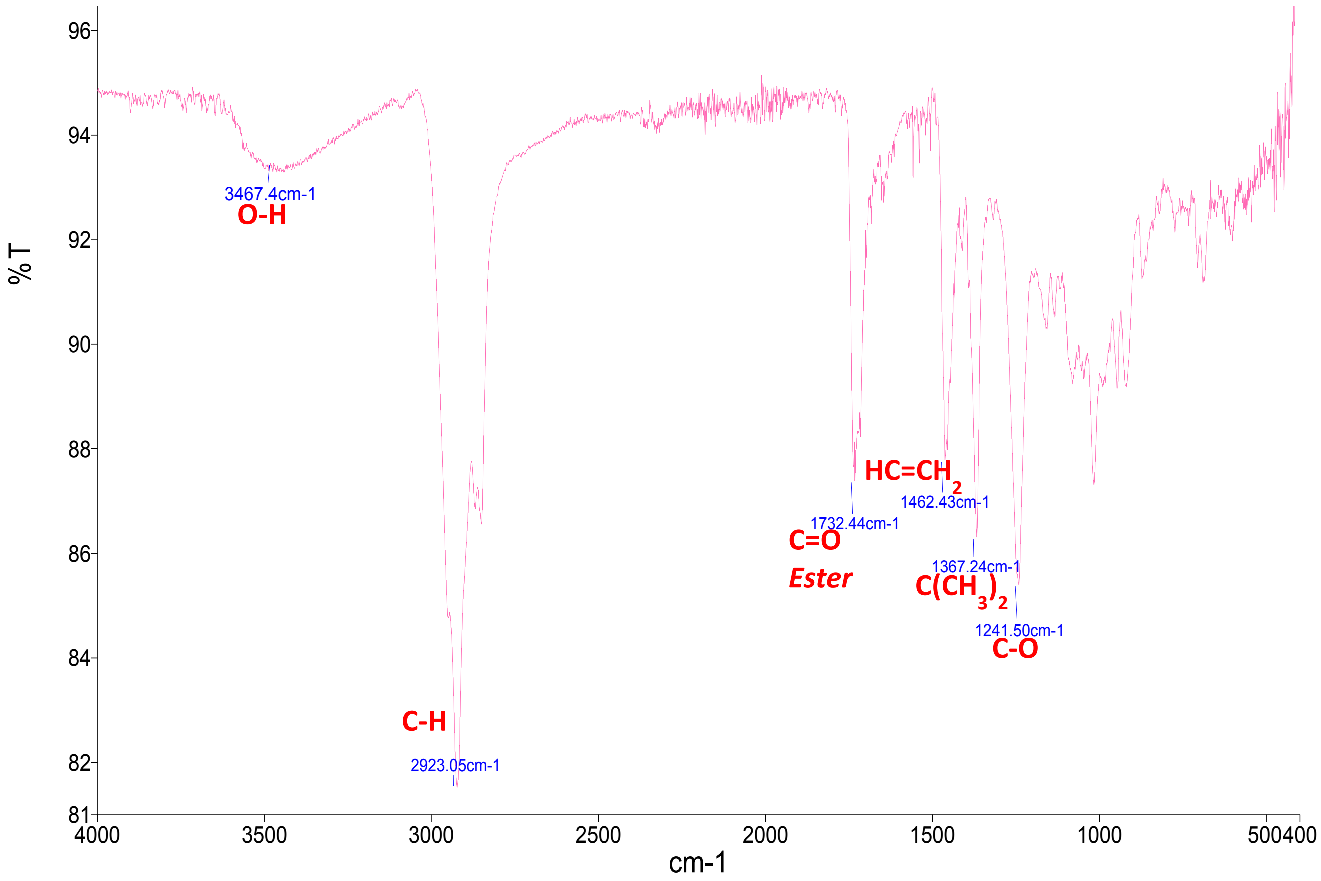

Figure S72. IR spectrum of compound **3**

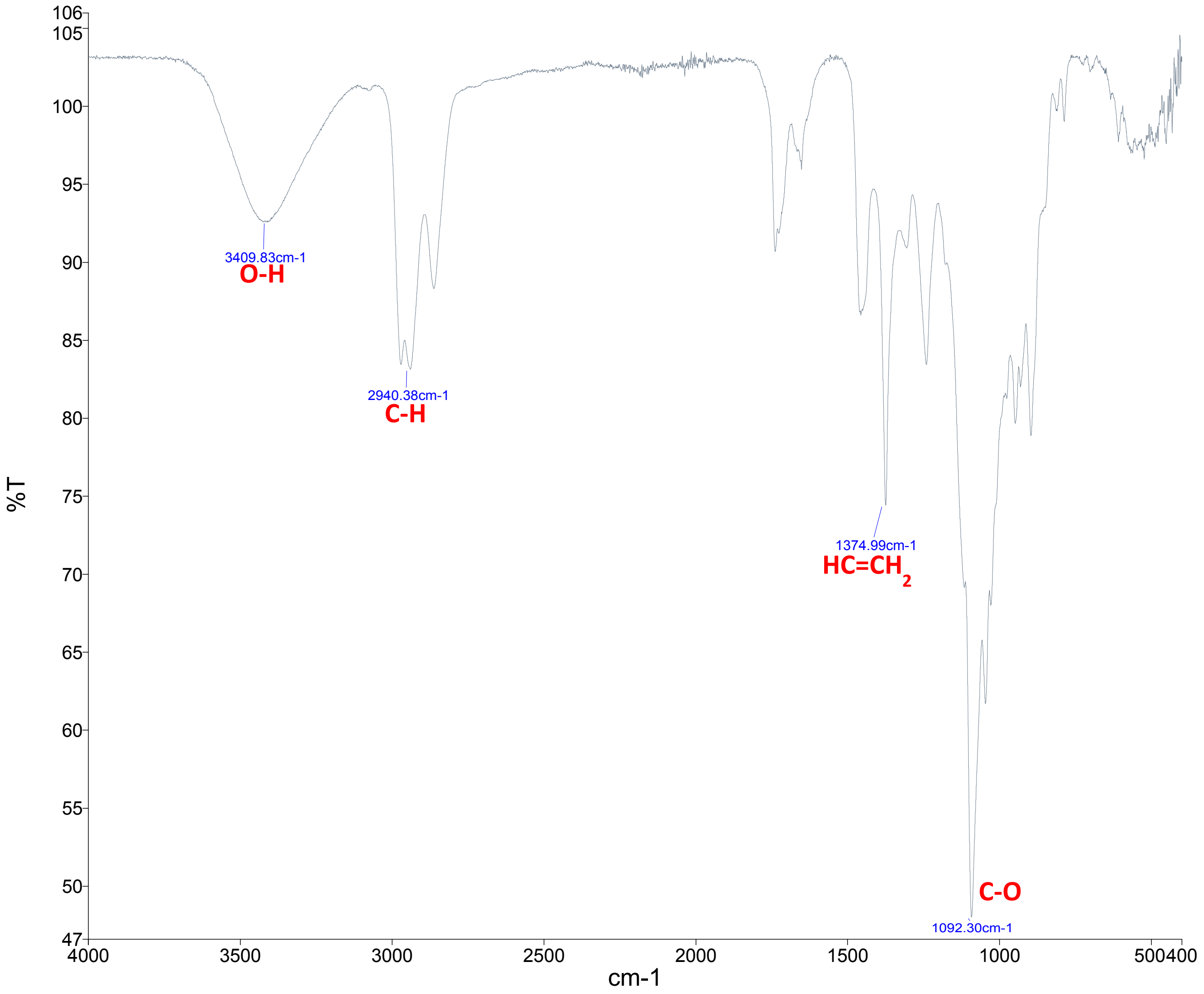

Figure S73. IR spectrum of compound **7**

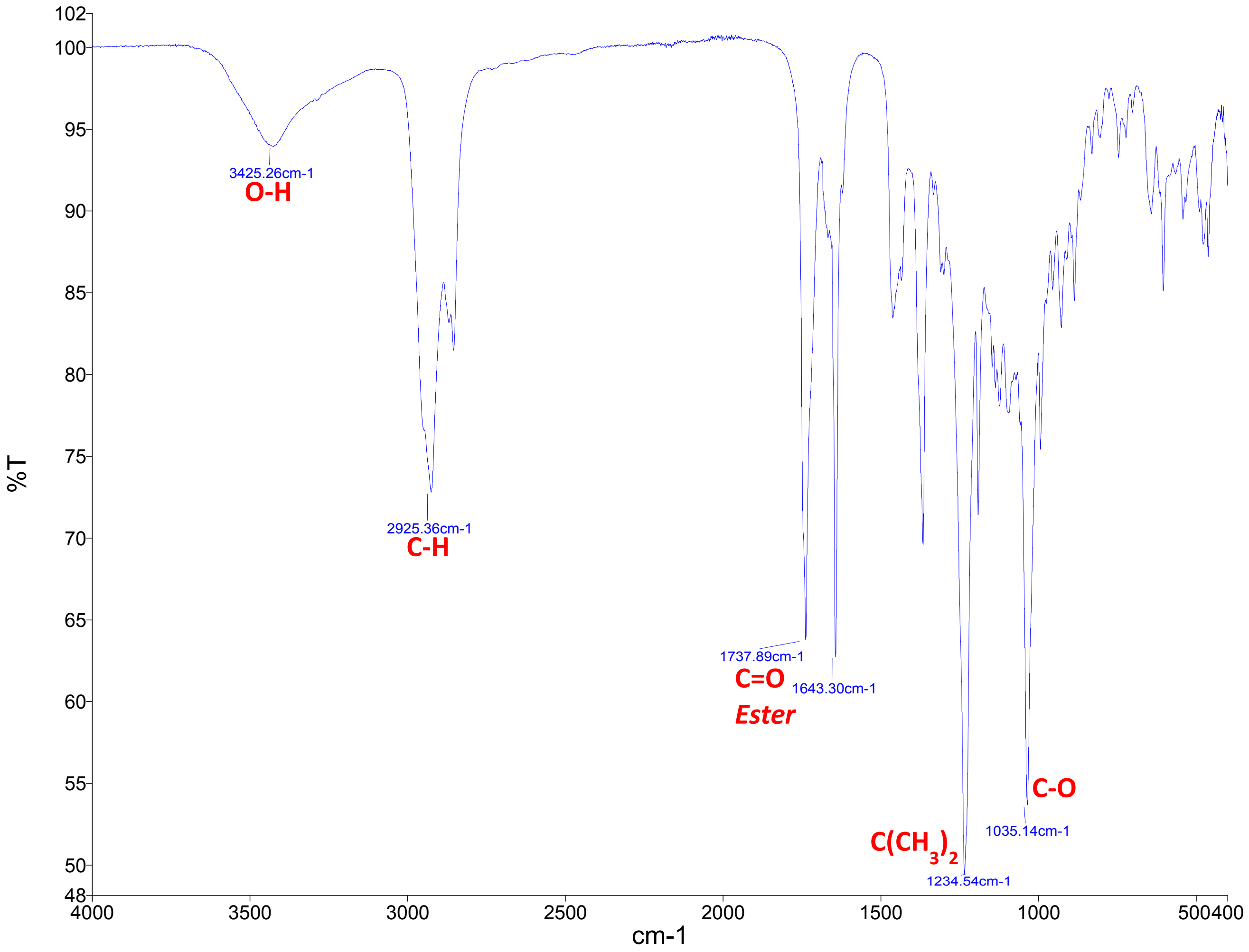

Figure S74. IR spectrum of compound **8**

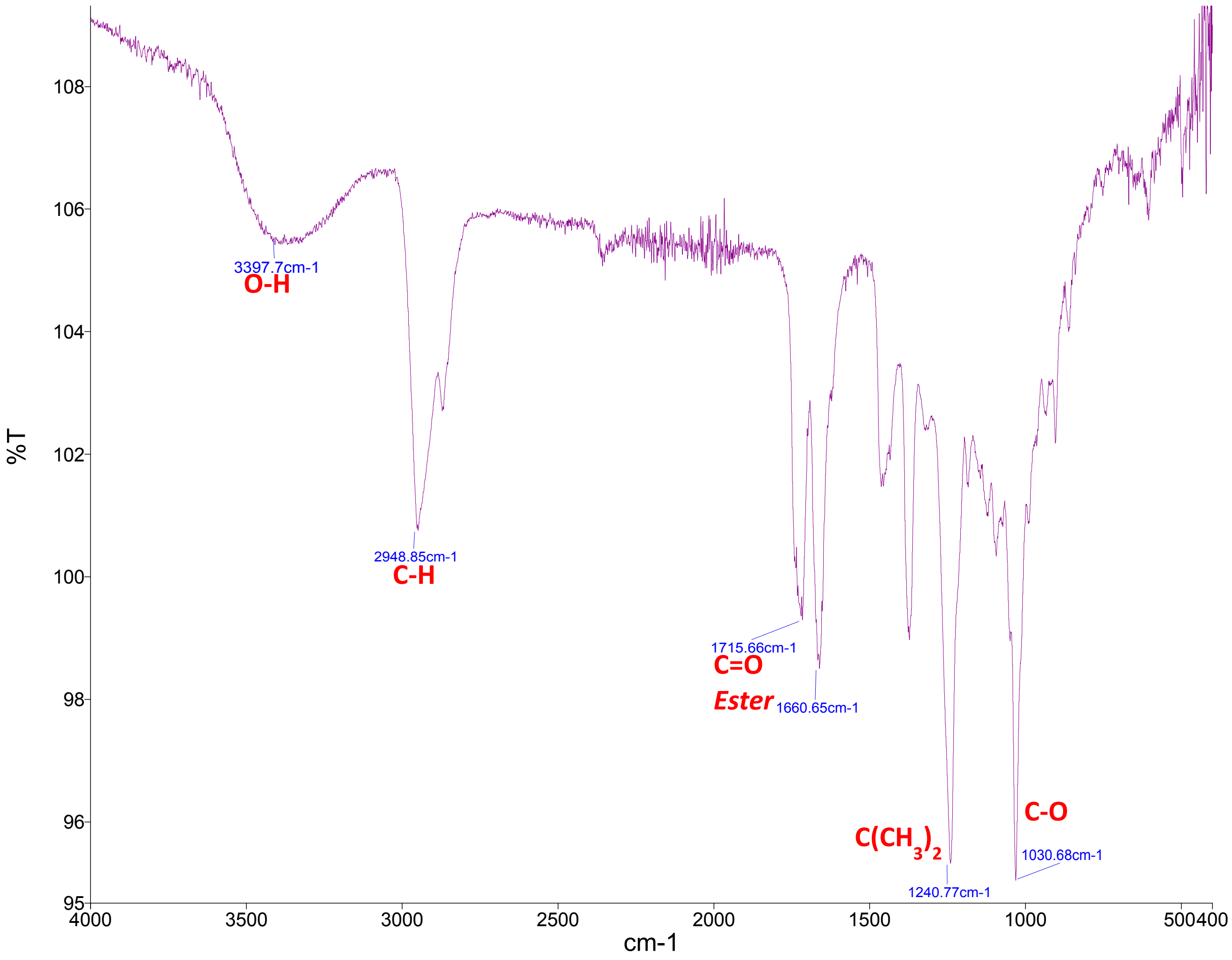

Figure S75. IR spectrum of compound **10**

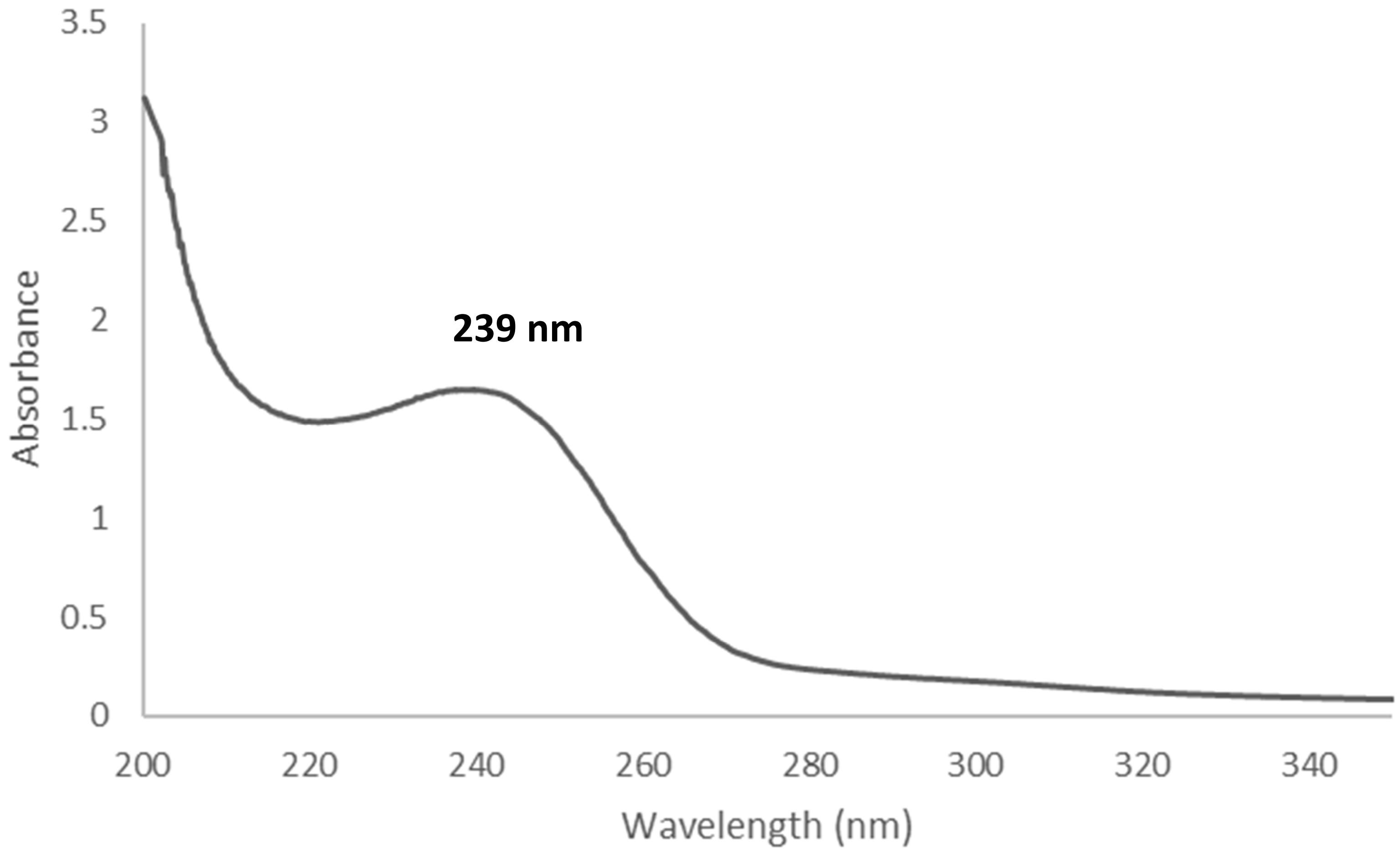

Figure S76. UV spectrum of compound **8**

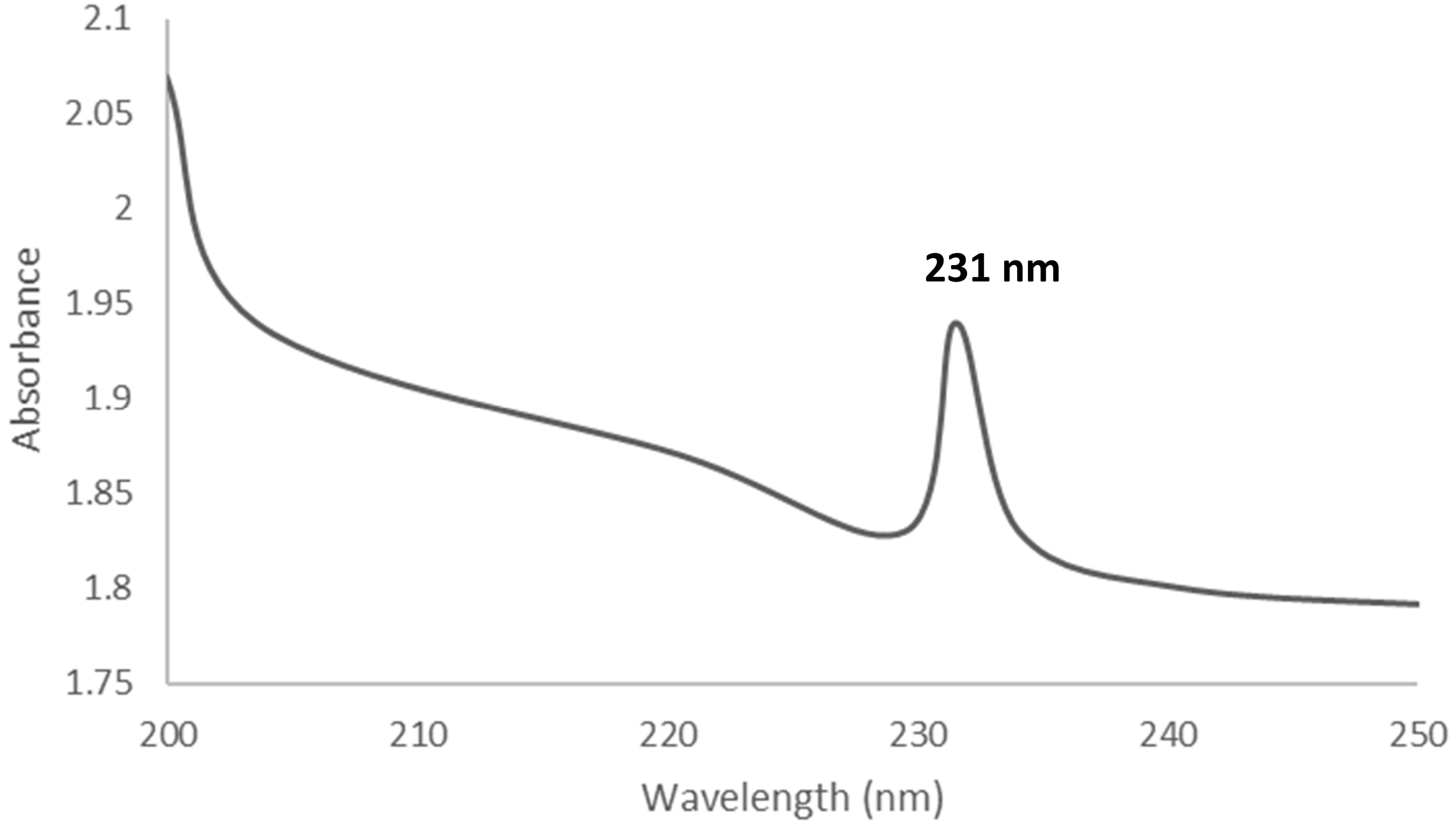

Figure S77. UV spectrum of compound **10**

Table S1. Cytotoxicity assay data for compounds **1 - 10**

	MDA-MB-231			HeLa		
**Compound**	**IC_{50} (μM)**	**SEM**	**R^2**	**IC_{50} (μM)**	**SEM**	**R^2**
**1**	26.3	1	0.9952	30.2	1.2	0.9309
**2**	53.7	1.2	0.8924	9.3	1.3	0.9215
**3**	58.5	1.3	0.9756	42	1.2	0.9346
**4**	44.4	1.1	0.9812	21.1	1.1	0.9584
**5 + 6**	13.5	1.1	0.9883	46.4	1.1	0.9822
**7**	8.8	5.6	0.9913	133.8	1.1	0.9716
**8**	27.6	1.1	0.9748	25.6	1.2	0.9304
**9**	15.8	1.1	0.9907	48.2	3.3	0.9732
**10**	21.6	1.1	0.9797	43.5	1.2	0.9213
